# Protein velocity and acceleration from single-cell multiomics experiments

**DOI:** 10.1186/s13059-020-1945-3

**Published:** 2020-02-18

**Authors:** Gennady Gorin, Valentine Svensson, Lior Pachter

**Affiliations:** 1 Division of Chemistry and Chemical Engineering, California Institute of Technology, Pasadena, USA; 2 Division of Biology and Biological Engineering & Department of Computing and Mathematical Sciences, California Institute of Technology, Pasadena, USA

**Keywords:** Protein acceleration, Protein velocity, RNA velocity, Transcriptomics, Multiomics, Bioinformatics, Computational biology

## Abstract

The simultaneous quantification of protein and RNA makes possible the inference of past, present, and future cell states from single experimental snapshots. To enable such temporal analysis from multimodal single-cell experiments, we introduce an extension of the RNA velocity method that leverages estimates of unprocessed transcript and protein abundances to extrapolate cell states. We apply the model to six datasets and demonstrate consistency among cell landscapes and phase portraits. The analysis software is available as the *protaccel* Python package.

## Background

Recent technological innovations that allow for assaying multiple modes of cell states at single-cell resolution are creating opportunities for more detailed biophysical modeling of the molecular biology of cells. Specifically, genome-wide probing of molecular states is revealing detailed information about the functional diversity of cells as determined by gene regulation, transcription, processing, and translation. The ability to probe cell states has been driven by improvements in single-cell RNA sequencing (scRNA-seq) methods [1] and advances in multiomics [2]. These methods allow researchers to quantify mRNA and protein expression levels in individual cells [3–5]. Furthermore, scRNA-seq can discriminate between nascent and processed transcripts. The recently described *RNA velocity* [6] method takes advantage of this feature of single-cell RNA-seq to fit a first-order system of ordinary differential equations describing gene-specific splicing [7] and to infer kinetic trajectories of single cells.

**Fig. 1:**
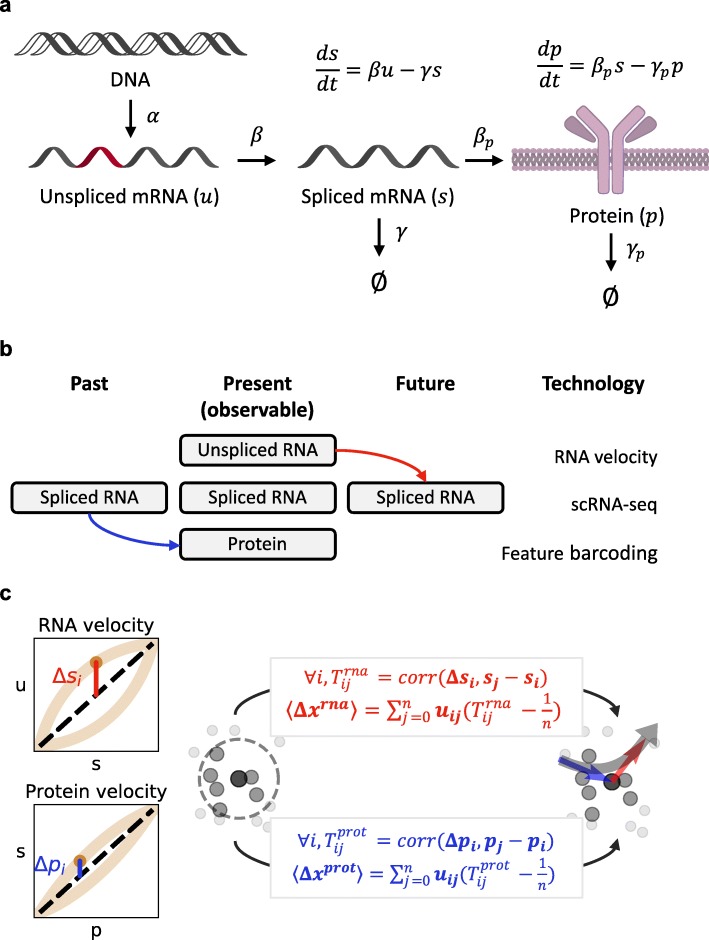
Model structure and parameter inference. **a** A single gene’s information transfer through transcription, splicing, and translation, and the ordinary differential equations governing the spliced mRNA and protein populations. **b** Conceptual framework for extrapolation from snapshot sequencing data. **c** Protein acceleration workflow: estimation of equilibrium states *u* = *γs* and *s* = *γ*_*p*_*p* (black dashed lines) from imputed gene-specific population data (light brown), gene-specific extrapolation to calculate Δ*s*_*i*_ and Δ*p*_*i*_, identification of nearest neighbors (dark gray: cell *i*, intermediate gray: *n* neighboring cells *j*, light gray: non-neighbor cells, circle: neighborhood), calculation of transition probabilities and embedded velocities (red: RNA velocity, blue: protein velocity, *T*_*ij*_: transition probability from cell *i* to neighbor *j*, *u*_*ij*_: unit vector from cell *i* to neighbor *j*), and visualization of acceleration (blue arrow: protein velocity, red arrow: RNA velocity, combined curvature: gray Bézier curve)

RNA velocity exploits the transfer of information in gene expression to extrapolate future cell states. In brief, the current population of unspliced transcripts is slated to be processed (Fig. 1a) and thus contains information regarding the future population of spliced transcripts. We extend this logic as follows [8]: the current population of proteins was translated from spliced RNA and thus contains information regarding the *past* population of spliced transcripts (Fig. 1b). We extend the RNA velocity model to protein translation, resulting in an analogous mathematical formulation for protein count extrapolation (Fig. 1a). We emphasize that unlike methods that require time-series measurements [9–11], our method estimates protein translation kinetics from a single time-point. To visualize the apparently disparate RNA and protein estimates in a single cell state representation, we adapt a method [6] for embedding dynamical information based on a distance metric in a high-dimensional space (Fig. 1c). We provide the Python package *protaccel* to facilitate analysis, and apply it to datasets to estimate their past and future cell states and identify trends in acceleration behavior.

## Results and discussion

We analyze six peripheral blood mononuclear cell (PBMC) datasets, collected using four different technologies. The dataset metadata is outlined in Table 1. The four technologies are CITE-seq [3], REAP-seq [4], ECCITE-seq [5] (with two datasets: “ctrl,” a healthy control, and “CTCL,” a cutaneous T cell lymphoma patient), and 10X Genomics feature barcoding (with two datasets: “1k” and “10k” cells).

**Table 1:** Protein acceleration datasets and parameters

Dataset	CITE-seq	REAP-seq	ECCITE-seq ctrl	ECCITE-seq CTCL	10X 1k	10X 10k
RNA data	GSM2695381	GSM2685238	GSM3596095	GSM3596100	See Methods	See Methods
Protein data	GSM2695382	GSM2685243	GSM3596096	GSM3596101	See Methods	See Methods
Alignment software	*Cell Ranger* 2.2	*Cell Ranger* 1.3	*Cell Ranger* 3.0	*Cell Ranger* 3.0	*kallisto* 0.46	*kallisto* 0.46
Counting software	*velocyto* 0.17	*velocyto* 0.17	*velocyto* 0.17	*velocyto* 0.17	*kallisto* 0.46	*kallisto* 0.46
Reference genome	GRCh38	hg19	hg19	hg19	GRCh38	GRCh38
Cell count	1780	3158	5084	5317	709	7855
Velocity genes	1172	1338	591	667	1114	920
Antibodies	10	41	49	49	17	17
Velocity proteins	7	16	11	12	7	8
Cell types found	5	4	4	3	5	5
Imputation *k*	400	800	800	800	50	50
Clustering method	MVP	RVP	RVP	MVP	MVP	MVP
Embedding	PC2/3 and t-SNE	t-SNE	t-SNE	t-SNE	t-SNE	t-SNE

*MVP* ModularityVertexPartition, *RVP* RBERVertexPartition, *PCA* principal component, *t-SNE* t-Stochastic Neighbor Embedding

The approximately linear spliced RNA/protein phase plots (Additional file 1: Figures S1-S6) are qualitatively consistent with the first-order and constant-parameter model of protein production, although we do observe some deviations by cell type. A subset of linear gene/protein pairs (Additional file 1: Table S1), manually selected from the phase plots according to concordance with the model, was used to estimate the gene-specific protein velocities. To calculate RNA velocity, we use a broad panel of genes with robust unspliced detection, high variation, and good agreement with the ODE model (sample genes and fits shown in Additional file 1: Figures S7-S12). We extrapolated the cell states, then embedded them in a projection calculated from the spliced mRNA space (Additional file 1: Supplementary Note).

The cell type-specific RNA velocities (Additional file 1: Figure S13-S18) depict a highly directional landscape. The corresponding protein velocities (Additional file 1: Figures S19-S24) are rather noisier as a result of sparser data collection (dozens of proteins vs. thousands of genes). We used a Gaussian kernel to determine the net velocities at regular grid points. The RNA and protein velocity fields (Additional file 1: Figures S25-S30) suggest that alignment between the two is strongly associated with cell type. The combination of RNA and protein velocities reveals the curvature of the cell state landscape. In a conceptual sense corresponding to Fig. 1b, the immediate protein velocity and the underlying RNA velocity yield a second-order estimate of *protein acceleration* driven by upstream unspliced mRNA modulation. We visualize cell movement in the embedding using a Bézier curve calculated from three points corresponding to past, present, and future cell states. A high-curvature Bézier curve corresponds to high acceleration.

**Fig. 2:**
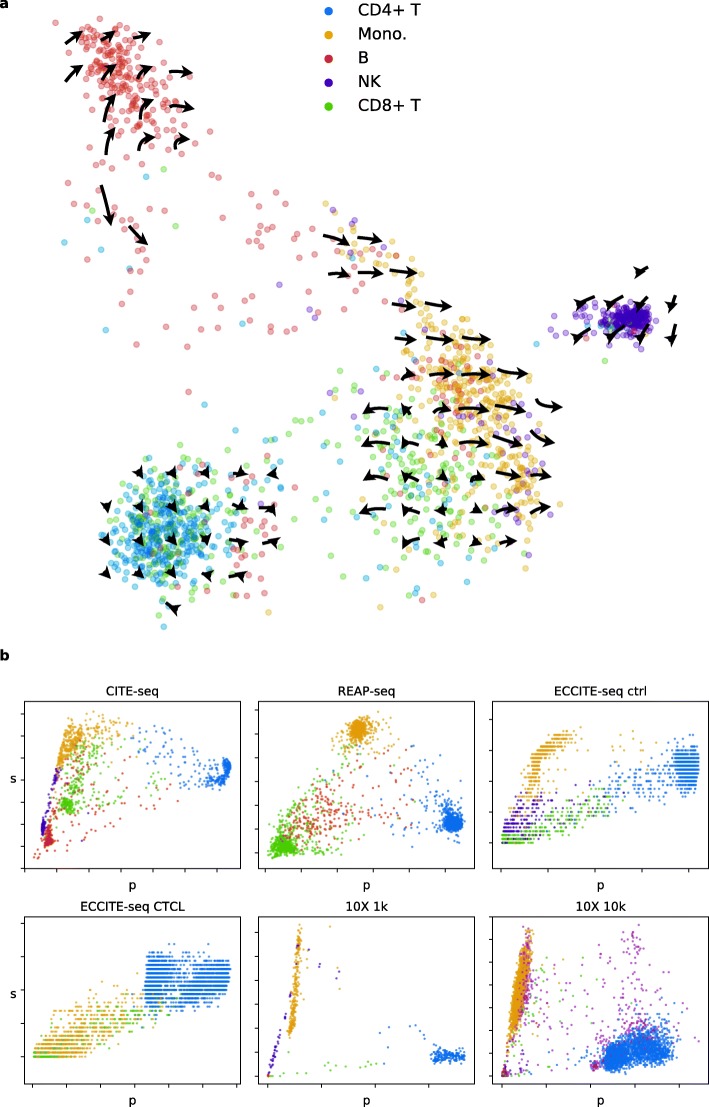
Protein acceleration visualization. **a** CITE-seq PBMC protein acceleration, visualized on a grid in principal component space. **b** Spliced RNA/protein phase portraits of CD4 in six PBMC datasets. Dot color identifies cell type (blue: CD4+ T, red: B, yellow: monocytes, green: CD8+ T, purple: natural killer, pink: not identifiable unambiguously)

The protein acceleration landscapes show a diversity of dynamics identifiable across datasets (Fig. 2a, Additional file 1: Figure S31-S36). A distinct set of B cells has high acceleration (CITE, REAP, 10X 1k, 10X 10k); another set, which forms a separate cluster in t-SNE, has low acceleration (CITE, REAP, 10X 10k). T lymphocytes tend to show low acceleration and low mobility in general (all datasets), although a mobile subset is occasionally seen and forms a cluster with mobile monocytes (CITE, REAP, possibly 10X 1k, possibly 10X 10k). Monocytes are mobile and exhibit a mixture of unidirectional and accelerated dynamics in a single cluster (CITE, REAP, ECCITE ctrl, 10X 1k, 10X 10k).

We found that subsets of B cells and of T lymphocytes exhibit strong protein acceleration. We hypothesize that the B cell partitioning corresponds to the differences between cell subtypes, e.g., mature B cells, which are resting [12] and require dedicated T cell activation [13], and plasma cells, which quickly respond to stimuli [14] and would be expected to have high acceleration on the relevant timescales. The T lymphocyte behavior may reflect recent findings that describe mRNA transcript “pile-up” due to heavily suppressed translation in naïve CD4+ T cells [15], or potential lymphocyte plasticity [16]. The monocyte behavior may correspond to the steady-state partitioning between monocyte subtypes [17], such as the transition from classical to non-classical circulating monocytes [18]. However, due to the imperfect separation of cell types in the embedding, we caution against over-interpretation of aggregated velocities.

The quality of data between the four different technologies is quite disparate. CITE-seq and 10X technologies appear to give strong velocity signals; inspection of raw phase portraits suggests that the results are fairly reliable (Additional file 1: Figures S1, S5-S7, S11-S12). REAP-seq yields lower RNA counts (Additional file 1: Figures S2, S8) and noisier dynamics. Finally, ECCITE-seq yields extremely sparse acceleration landscapes (Additional file 1: Figures S33-S34), which result from the very shallow sequencing of spliced transcripts: we confirmed that ECCITE-seq captures 1–2 orders of magnitude fewer RNA molecules per cell than CITE-seq or REAP-seq, which is consistent with Fig. 1b of Mimitou et al. [5] (Additional file 1: Figure S37). Comparison to unfiltered pseudoaligned 10X data shows that the ECCITE-seq RNA counts are roughly comparable to counts in empty droplets in the 10X feature barcoding technology. Overall, the CITE-seq and feature barcoding technologies appear to be by far the most reliable.

In addition to using genes with linear behavior to infer velocity, we qualitatively confirmed consistency between datasets for the gene CD4, which has a striking non-linear appearance (Fig. 2b). We hypothesize that the non-linear behavior corresponds to regulatory differences due to cell type; in the context of our model, the data seem to suggest a unique, low degradation rate in CD4+ T lymphocytes and a different, high degradation rate in all other blood cell types.

Our qualitative protein acceleration framework does not attempt to account for regulatory differences between cell types. Future work may involve more granular models to enable inference of *local* rather than global parameters, e.g., the determination of separate parameters for the CD4+ T lymphocytes and other cell types for the CD4 gene (Fig. 2b). Current protein quantification protocols are adapted for histological markers on the cell surface; technology that can quantify cytosolic protein could aid in more extensive studies of cell dynamics and open a broader range of investigations tractable by protein acceleration, as discussed in Additional file 1: Supplementary Note. In particular, we anticipate this method is naturally applicable to inferring and validating cell state vector fields [19]. Finally, the simultaneous quantification of mRNA and *regulatory* cytosolic proteins would greatly aid in the implementation of physically realistic models of gene expression which explicitly account for regulation by observed transcription factors.

## Methods

The key metadata, physiology, and parameters used for the six datasets are summarized in Table 1. GSM numbers correspond to Gene Expression Omnibus (GEO) samples. 10X Genomics PBMC datasets are available at the 10X Genomics website [20, 21]. Aligned sequence files released alongside each original publication were used whenever available. The *velocyto* 0.17 command-line interface was used to generate unspliced count matrices for the CITE-seq, REAP-seq, and ECCITE-seq datasets; *kallisto* 0.46 was used for the 10X datasets.

The velocity calculation and visualization processes are described in detail in Additional file 1: Supplementary Note. In brief, scRNA-seq and feature barcoding data were smoothed using a nearest-neighbor connectivity matrix, generated using the *scikit-learn* 0.20.0 Python package [22]. For each cell, unspliced RNA, spliced RNA, and protein counts were replaced with the mean value of *k* neighbor cells. For ease of visualization, Louvain clustering was performed using the *louvain* 0.6.1 Python package [23]. Cell types were manually assigned based on markers (Additional file 1: Table S2) reported in CITE-seq and REAP-seq publications [3, 4] (Additional file 1: Figures S38-S43).

We implemented the protein acceleration workflow as the *protaccel* Python package [24]. *protaccel* 0.2 was used for all analyses in this article, with the exception of Additional file 1: Figures S52-S54, which used *protaccel* 0.301. To calculate RNA velocities, we fit extreme quantiles of the imputed spliced/unspliced RNA phase plots, filtered to select “velocity genes” with phase plots described sufficiently well by the linear fit (*R*^2^ > 0.1), estimated the spliced RNA degradation rates, then calculated deviations from the equilibrium line. To calculate protein velocities, we followed the same process, albeit using protein/spliced RNA phase plots and manually selecting “velocity proteins” with qualitatively linear phase plot appearance.

To visualize the velocities, we generated low-dimensional embeddings for the cells, selected to be a set of principal components (PCs) for CITE-seq and a t-Stochastic Neighbor Embedding (t-SNE) based on the top 25 PCs for all other datasets, as well as CITE-seq in Additional file 1: Figure S31 [25]. The PC and t-SNE calculations were performed using the *scikit-learn* 0.20.0 Python package [22]. Consistently with the original RNA velocity publication [6], we assumed the net velocity direction can be represented on a low-dimensional embedding by calculating transition probabilities to an embedding neighborhood of 500 cells. We computed these transition probabilities by calculating the correlation between high-dimensional velocity and directions to the embedding neighbors, both processed with a variance-stabilizing square root transformation. The high-dimensional space of the RNA velocity workflow is the space of velocity genes; the corresponding high-dimensional space of the protein velocity workflow is the space of velocity proteins. Each embedding was partitioned into a 20 × 20 point grid, representing cell states at *t*_0_; grid arrows were generated by applying a Gaussian kernel (smoothing parameter *σ* = 0.5) to the cell-specific velocities of 200 cells nearest the grid point. The forward extrapolation of each grid point, corresponding to information about *t*_+1_ inferred from RNA velocity, was calculated by adding the aggregated RNA velocity vector to the grid point vector. The backward extrapolation of each grid point, corresponding to information about *t*_−1_ inferred from protein velocity, was calculated by subtracting the aggregated protein velocity vector from the grid point vector. We produced curved arrows corresponding to the entire trajectory by fitting a second-order Bézier curve to each grid point’s *t*_−1_, *t*_0_, and *t*_+1_ locations. The fit was performed using the *bezier* 0.9.0 Python package.

We performed all simulations using MathWorks MATLAB 2018a.

Scripts to reproduce the results of this paper are available at GitHub [26]. Raw datasets for protein acceleration analysis (*velocyto* loom files with mRNA counts and csv files with protein counts) are available on figshare [27–30].

## Supplementary information

**Additional file 1.** Supplementary Information for “Protein velocity and acceleration from single-cell multiomics experiments.” Supplementary note describing the theory and implementation of protein velocity, and including supplementary figures.

**Additional file 2.** Review history.

## Data Availability

CITE-seq RNA and protein data were acquired from Gene Expression Omnibus samples GSM2695381 and GSM2695382 [31]. REAP-seq RNA and protein data were acquired from GSM2685238 and GSM2685243 [32]. ECCITE-seq control protein data were acquired from GSM3596096 [33]. ECCITE-seq CTCL protein data were acquired from GSM3596101 [33]. Due to patient privacy concerns, raw ECCITE-seq RNA data (GSM3596095 and GSM3596100) were not available, and the gene count matrices generated by *velocyto* were acquired by personal request. 10X Genomics 1k and 10k PBMC datasets were acquired from the 10X Genomics website [20, 21]. The datasets generated during this study are available on figshare [27–30].The Jupyter scripts used to analyze them are available on GitHub [26]. The *protaccel* Python package is available for installation through PyPi [24], and may be acquired as a script from GitHub [26] or Zenodo [34] under the BSD-2-Clause license.
