# Supplementary material for: Protein velocity and acceleration from single-cell multiomics experiments
Source: Genome Biol. 2020 Feb 18;21:39. doi: 10.1186/s13059-020-1945-3 (PMC7029606; doi:10.1186/s13059-020-1945-3)
Supplement: Supplementary file 1 — Additional file 1. Supplementary Information for “Protein velocity and acceleration from single-cell multiomics experiments.” Supplementary note describing the theory and implementation of protein velocity, and including supplementary figures. [file 13059_2020_1945_MOESM1_ESM.docx]

Supplementary Information for “Protein velocity and acceleration from single-cell multiomics experiments”

Gennady Gorin, Valentine Svensson, and Lior Pachter

Contents

[Supplementary Note 2](#_Toc27582304)

[Theoretical background 2](#_Toc27582305)

[RNA velocity 2](#_Toc27582306)

[Protein velocity 2](#_Toc27582307)

[Velocity workflow 3](#_Toc27582308)

[Pre-processing 3](#_Toc27582309)

[Estimation of degradation parameters 3](#_Toc27582310)

[Velocity calculation 3](#_Toc27582311)

[Embedding process: RNA velocity 4](#_Toc27582312)

[Embedding process: Protein velocity 5](#_Toc27582313)

[Embedding process: Protein acceleration curves 5](#_Toc27582314)

[Quantitative considerations and assumptions 5](#_Toc27582315)

[Validation 7](#_Toc27582316)

[Model description and physiological parameter selection 8](#_Toc27582317)

[Determination of previously validated parameter regions 10](#_Toc27582318)

[Methodological directions, quantitative and qualitative 11](#_Toc27582319)

[Directions for applications 12](#_Toc27582320)

[References 13](#_Toc27582321)

[Supplementary Figures 18](#_Toc27582322)

[Protein velocity phase plots 18](#_Toc27582323)

[RNA velocity phase plots 23](#_Toc27582324)

[Cluster-specific RNA velocity 29](#_Toc27582325)

[Cluster-specific protein velocity 35](#_Toc27582326)

[Combined velocity visualizations 41](#_Toc27582327)

[Acceleration landscapes 47](#_Toc27582328)

[Sequencing quality comparison 53](#_Toc27582329)

[Cell type identification 54](#_Toc27582330)

[Validation 57](#_Toc27582331)

# Supplementary Note

## Theoretical background

### RNA velocity

We assume that a gene’s abundance of unspliced RNA $u$ and spliced RNA $s$ is determined by the following first-order ordinary differential equations (ODE):

$\frac{du}{dt}=\alpha\left( t \right)-\beta\left( t \right) u$;

$\frac{ds}{dt}=\beta\left( t \right) u-\gamma\left( t \right) s$.

In this general model, the transcription, splicing, and degradation parameters $\alpha, \beta,\gamma$ are gene- and time-dependent. Neglecting the time dependence produces a simplified system for each gene:

$\frac{du}{dt}=\alpha-\beta u$;

$\frac{ds}{dt}=\beta u-\gamma s$.

Under the assumptions underlying the RNA velocity framework (1), $\beta$ is a common, gene-independent splicing parameter. Dividing by $\beta$ yields:

$\frac{1}{\beta}\frac{du}{dt}=\frac{\alpha}{\beta}-u=\hat{\alpha}-u$;

$\frac{1}{\beta}\frac{ds}{dt}=u-\frac{\gamma s}{\beta}=u-\hat{\gamma}s$.

The equilibrium line is defined by $\frac{ds}{dt}=0=u-\hat{\gamma}s$, yielding $\hat{\gamma}=u/s$. Therefore, the rate of change in spliced counts scales with deviation from equilibrium.

## Protein velocity

Protein production and degradation are governed by the following equation:

$\frac{dp}{dt}=\beta_{p}\left( t \right) s- \gamma_{p}\left( t \right) p$.

Assuming constant parameters, this results in:

$\frac{dp}{dt}=\beta_{p}s- \gamma_{p} p$;

$\frac{1}{\beta_{p}}\frac{dp}{dt}=s-\frac{\gamma_{p} p}{\beta_{p}}=s-\hat{\gamma}_{p} p$.

Following the RNA velocity framework, we can use a similar approximation and assume the translation rate is gene-independent. The corresponding equilibrium line is defined by $\frac{dp}{dt}=0=s-\hat{\gamma}_{p}p$, yielding $\hat{\gamma}_{p}=s/p$. The rate of change in protein counts scales with deviation from this equilibrium.

## Velocity workflow

### Pre-processing

To improve the velocity estimation and ameliorate the discrete nature of the sequencing data, we pooled RNA and protein counts across neighboring cells, imputing each measurement with the mean of $k$ nearest neighbors. The authors of original RNA velocity publication performed the imputation based on neighborhood in the spliced mRNA count space, normalized to the total number of spliced mRNA in each cell and variously transformed. For a cell vector ${\bar{\boldsymbol{s}}}_{\boldsymbol{i}}$ in the normalized spliced space$,$ they identified the $k$ neighbors, indexed by $j$, with lowest Euclidian distance $\left\| {\bar{\boldsymbol{s}}}_{\boldsymbol{i}}\boldsymbol{-}{\bar{\boldsymbol{s}}}_{\boldsymbol{j}} \right\|$ (1). We found that considering the high-dimensional protein count vector $\boldsymbol{p}_{\boldsymbol{i}}$ produced more robust results. Thus, we normalize $\boldsymbol{p}_{\boldsymbol{i}}$ by the total number of proteins in each cell to produce the vector ${\bar{\boldsymbol{p}}}_{\boldsymbol{i}}$, and identify the $k$ neighbors with lowest Euclidian distance $\left\| {\bar{\boldsymbol{p}}}_{\boldsymbol{i}}\boldsymbol{-}{\bar{\boldsymbol{p}}}_{\boldsymbol{j}} \right\|$. Finally, we produce the per-cell imputed vectors ${\hat{\boldsymbol{p}}}_{i}=\frac{1}{k}\sum_{j=1}^{k} \boldsymbol{p}_{\boldsymbol{j}}$, ${\hat{\boldsymbol{s}}}_{i}=\frac{1}{k}\sum_{j=1}^{k} \boldsymbol{s}_{\boldsymbol{j}}$, and ${\hat{\boldsymbol{u}}}_{i}=\frac{1}{k}\sum_{j=1}^{k} \boldsymbol{u}_{\boldsymbol{j}}$.

### Estimation of degradation parameters

Per the ODE model, at steady state, $\hat{\gamma}=u/s$ and $\hat{\gamma}_{p}=s/p$ for each gene. Following the procedure used by La Manno et al. (1), we assume that the steady states lie at the diagonal extrema of the phase plots.

For the RNA velocity workflow, we estimate maximum $\hat{u}$ and $\hat{s}$ values for each gene by calculating the $99.9\%$ quantiles across all cells, yielding the values $\hat{u}_{max}$ and $\hat{s}_{max}$. We calculate normalized gene-specific vectors$\tilde{\boldsymbol{u}}=$ $\frac{\hat{\boldsymbol{u}}}{\hat{u}_{max}}$ and $\tilde{\boldsymbol{s}}=$ $\frac{\hat{\boldsymbol{s}}}{\hat{s}_{max}}$, and identify the extreme $2\%$ and $98\%$ quantiles $n_{low}$ and $n_{high}$ of the sum $\tilde{\boldsymbol{n}}=\tilde{\boldsymbol{u}}\boldsymbol{+}\tilde{\boldsymbol{s}}$. At this stage, cells with $\tilde{n}>n_{high}$ are classified as residing in the high-expression steady state, whereas cells with $\tilde{n}<n_{low}$ are classified as residing in the low-expression steady state. We estimate the RNA degradation rate $\hat{\gamma}$ as the slope of a line fit to these two populations of cells.

For the protein velocity workflow, we follow the same steps, albeit with $\hat{p}$ replacing $\hat{s}$ and $\hat{s}$ replacing $\hat{u}$. We estimate the protein degradation rate $\hat{\gamma}_{p}$ as the slope of a line fit to the two populations of cells at the extrema.

### Velocity calculation

Since not all genes are expected to express dynamics consistent with the simple ODE model, we filter the selection of genes used for the velocity calculations. For RNA velocity, we perform three rounds of filtering, omitting genes with a low coefficient of variability relative to mean, extremely low expression, and low $R^{2}$ of the phase plots relative to the linear fit produced to estimate $\hat{\gamma}_{s}$. Due to the low dimensionality of the protein data, we manually select spliced RNA-protein pairs for protein velocity; we omit gene/protein pairs that have phase portraits without a self-evident linear component, and choose only a single subunit for analysis (e.g. CD3D/CD3 rather than all 3 subunits of CD3). We report the resulting $R^{2}$ values along with the phase portraits.

We calculate the RNA velocity for each cell $i$ and each selected gene $g$ using the imputed state vectors $\hat{u}_{i,g}$ and $\hat{s}_{i,g}$, as well as the previously calculated gene-specific degradation rate $\hat{\gamma}_{g}$:

$$v_{i,g,rna}\boldsymbol{=}\hat{u}_{i,g}\boldsymbol{-}\hat{\gamma}_{g}\hat{s}_{i,g}$$

Finally, we calculate the protein velocity for each cell $i$ and each selected spliced RNA-protein pair $g$ using the imputed state vectors $\hat{s}_{i,g}$ and $\hat{p}_{i,g}$, as well as the previously calculated gene-specific degradation rate $\hat{\gamma}_{p,g}$:

$$v_{i,g,protein}\boldsymbol{=}\hat{s}_{i,g}\boldsymbol{-}\hat{\gamma}_{p,g}\hat{p}_{i,g}$$

Assuming unity splicing rates $\beta$, we can extrapolate for an arbitrary $\Delta t$, yielding $\boldsymbol{\Delta}\boldsymbol{s}_{\boldsymbol{i}}=\boldsymbol{v}_{\boldsymbol{i,rna}} \Delta t$ for each cell $i$. As the timescale is arbitrary, the *direction* is more meaningful than the magnitude of the extrapolated vector. For unity $\Delta t$, the expression reduces to $\boldsymbol{\Delta}\boldsymbol{s}_{\boldsymbol{i}}=\boldsymbol{v}_{\boldsymbol{i,rna}}$.

Analogously, assuming unity translation rates $\beta_{p}$, we can extrapolate for an arbitrary $\Delta t$, yielding $\boldsymbol{\Delta}\boldsymbol{p}_{\boldsymbol{i}}=\boldsymbol{v}_{\boldsymbol{i,protein}}\Delta t$ for each cell $i$. Again, the magnitude is arbitrary; for unity $\Delta t$, the expression reduces to $\boldsymbol{\Delta}\boldsymbol{p}_{\boldsymbol{i}}=\boldsymbol{v}_{\boldsymbol{i,protein}}$.

### Embedding process: RNA velocity

The estimation of the velocity directions in a low-dimensional embedding closely follows the procedure outlined in the original RNA velocity publication (1).

The process of RNA velocity embedding, adapted from the original description, proceeds as follows. For cell $i$ with high-dimensional imputed spliced mRNA vector ${\hat{\boldsymbol{s}}}_{\boldsymbol{i}}$ and low-dimensional embedding vector $\boldsymbol{x}_{\boldsymbol{i}}$, we identify $m$ nearest embedding neighbors, indexed by $j$, with lowest embedding Euclidian distance $\left\| \boldsymbol{x}_{\boldsymbol{j}}\boldsymbol{-}\boldsymbol{x}_{\boldsymbol{i}} \right\|$.

We postulate that the cell will transition in a direction representable by a weighted sum of directions to the $m$ neighbors. Further, we assume that the weights are interpretable as probabilities determined by the alignment between the RNA velocity vector and the directions to the nearest neighbors in high-dimensional space. Thus, for each $i$, we find a *transition* *probability* vector $\boldsymbol{T}_{\boldsymbol{i}}$ such that:

$$\boldsymbol{T}_{\boldsymbol{ij}}=\frac{\exp\left( corr\left( \boldsymbol{r}_{\boldsymbol{ij}},\boldsymbol{d}_{\boldsymbol{i}} \right)/\sigma\right)}{\sum_{j=1}^{m} \exp\left( corr\left( \boldsymbol{r}_{\boldsymbol{ij}},\boldsymbol{d}_{\boldsymbol{i}} \right)/\sigma\right)}$$

$\sigma$ is a constant smoothing parameter or width of the kernel. $\boldsymbol{d}_{\boldsymbol{i}}$ and $\boldsymbol{r}_{\boldsymbol{ij}}$ are vectors corresponding to variance-stabilizing transformations of the RNA velocity and the directions to nearest neighbors:

$$\boldsymbol{d}_{\boldsymbol{i}}=sgn\left( \boldsymbol{\Delta}\boldsymbol{s}_{\boldsymbol{i}} \right)\sqrt{\boldsymbol{\Delta}\boldsymbol{s}_{\boldsymbol{i}}}$$

$$\boldsymbol{r}_{\boldsymbol{ij}}= sgn\left( {\hat{\boldsymbol{s}}}_{\boldsymbol{j}}\boldsymbol{-}{\hat{\boldsymbol{s}}}_{\boldsymbol{i}} \right)\sqrt{{\hat{\boldsymbol{s}}}_{\boldsymbol{j}}\boldsymbol{-}{\hat{\boldsymbol{s}}}_{\boldsymbol{i}}}$$

Where $sgn(x)$ is the signum function and $\boldsymbol{\Delta}\boldsymbol{s}_{\boldsymbol{i}}$ is the extrapolated direction in imputed spliced mRNA space, calculated as above.

For each neighbor $j$, there is a unit direction vector $\boldsymbol{u}_{\boldsymbol{ij}}$ defined by:

$$\boldsymbol{u}_{\boldsymbol{ij}}=\frac{\boldsymbol{x}_{\boldsymbol{j}}\boldsymbol{-}\boldsymbol{x}_{\boldsymbol{i}}}{\left\| \boldsymbol{x}_{\boldsymbol{j}}\boldsymbol{-}\boldsymbol{x}_{\boldsymbol{i}} \right\|}$$

We calculate the net direction of the embedded velocity by weighing the directions to neighboring cells and correcting for density:

$$\boldsymbol{\Delta}\boldsymbol{x}_{\boldsymbol{i}}=\sum_{j=1}^{m} \left( \boldsymbol{T}_{\boldsymbol{ij}}-\frac{1}{m} \right)\boldsymbol{u}_{\boldsymbol{ij}}$$

To generate grid arrows, we aggregate the velocities from $l$ cells nearest each grid point. For each grid point $\boldsymbol{x}_{\boldsymbol{grid}}$, the $l$ nearest cells have the lowest embedding Euclidian distances $\left\| \boldsymbol{x}_{\boldsymbol{i}}\boldsymbol{-}\boldsymbol{x}_{\boldsymbol{grid}} \right\|$. To calculate the net velocity vector, we apply a Gaussian smoothing kernel:

$$\boldsymbol{\Delta}\boldsymbol{x}_{\boldsymbol{grid}}=\sum_{i=1}^{l} \boldsymbol{\Delta}\boldsymbol{x}_{\boldsymbol{i}}\exp\left( -\frac{\left\| \boldsymbol{x}_{\boldsymbol{i}}\boldsymbol{-}\boldsymbol{x}_{\boldsymbol{grid}} \right\|}{\sigma_{grid}} \right)$$

### Embedding process: Protein velocity

The protein velocity embedding process is identical save for one difference. The vectors $\boldsymbol{d}_{\boldsymbol{i}}$ and $\boldsymbol{r}_{\boldsymbol{ij}}$ correspond to variance-stabilizing transformations of the *protein* velocity and the directions to nearest neighbors in high-dimensional *protein* space:

$$\boldsymbol{d}_{\boldsymbol{i}}=sgn\left( \boldsymbol{\Delta}\boldsymbol{p}_{\boldsymbol{i}} \right)\sqrt{\boldsymbol{\Delta}\boldsymbol{p}_{\boldsymbol{i}}}$$

$$\boldsymbol{r}_{\boldsymbol{ij}}= sgn\left( {\hat{\boldsymbol{p}}}_{j}\boldsymbol{-}{\hat{\boldsymbol{p}}}_{i} \right)\sqrt{{\hat{\boldsymbol{p}}}_{j}\boldsymbol{-}{\hat{\boldsymbol{p}}}_{i}}$$

Where $\boldsymbol{\Delta}\boldsymbol{p}_{\boldsymbol{i}}$ is the extrapolated direction in imputed protein space, calculated as above, and ${\hat{\boldsymbol{p}}}_{\boldsymbol{i}}$ is the imputed protein cell vector. The cell-specific and grid arrows are calculated identically to the RNA velocity workflow.

### Embedding process: Protein acceleration curves

Given a grid point $\boldsymbol{x}_{\boldsymbol{grid}}$ and associated velocity vectors $\boldsymbol{\Delta}\boldsymbol{x}_{\boldsymbol{grid,rna}}$ and $\boldsymbol{\Delta}\boldsymbol{x}_{\boldsymbol{grid,protein}}$ generated from the RNA and protein velocity workflows respectively, we simultaneously plot the point and the vectors on the embedding. However, RNA velocity generates a *forward* direction estimate, whereas protein velocity generates a *backward* estimate. Therefore, we place the *tail* of the aggregated RNA velocity vector and the *head* of the protein velocity vector at the grid point.

Per our model, the *tail* of the protein velocity vector ($\boldsymbol{x}_{\boldsymbol{grid}}\boldsymbol{-}\boldsymbol{\Delta}\boldsymbol{x}_{\boldsymbol{grid,protein}}$) corresponds to the extrapolated past time $t_{-1}$, the grid point ($\boldsymbol{x}_{\boldsymbol{grid}}$) corresponds to the observed time $t$, and the *head* of the RNA velocity vector ($\boldsymbol{x}_{\boldsymbol{grid}}\boldsymbol{+\Delta}\boldsymbol{x}_{\boldsymbol{grid,rna}}$) corresponds to the extrapolated future time $t_{+1}$. All of these points lie within the embedding generated from normalized spliced mRNA counts. We qualitatively visualize the curvature of the cell’s direction in the embedding space by fitting a second-order Bézier curve to these three points in order from past to future, and place an arrow at $\boldsymbol{x}_{\boldsymbol{grid}}\boldsymbol{+\Delta}\boldsymbol{x}_{\boldsymbol{grid,rna}}$ to indicate the direction of traversal.

## Quantitative considerations and assumptions

The inference of protein velocity requires a number of assumptions, some novel and some inherited from the RNA velocity workflow. We outline these assumptions and discuss their relative quality in **Table S3**. The table is roughly divided to address assumptions about data quality, parameter values, the velocity calculation process, and the embedding process.

The assumptions regarding data quality largely concern the relationship between observed molecule counts and true molecule counts. Both methods assume that no substantial bias exists and the observed counts are reflective of true counts. The use of oligo-conjugated antibodies requires more detailed analysis of this assumption, as multiple antibodies may bind to the same protein. To our knowledge, antibody/antigen binding stoichiometries are not quantitatively characterized even for relatively common antibodies; we argue that this creates at most $O(1)$ error in protein velocity estimates, equivalent to the assumption of constant translation rates.

For $n \mathrm{ADT}:1 \mathrm{protein}$ binding stoichiometry, assuming saturation, the observed number of observed ADT for $p$ proteins is $A=np;p=A/n$. The protein velocity rate equation is:

$$\frac{dp}{dt}=\beta_{p}s-\gamma_{p}p$$

Plugging in the observable $A$:

$$\frac{d\left( \frac{A}{n} \right)}{dt}=\beta_{p}s-\gamma_{p}\left( \frac{A}{n} \right)=\frac{1}{n}\frac{dA}{dt}=\beta_{p}s-\frac{\gamma_{p}A}{n}$$

Expressing $t$ in units of $\frac{\beta_{p}}{n}$,

$$\frac{dA}{dt}=s-\hat{\gamma}_{p}A$$

$n>0, n \sim O\left( 1 \right)$, so this is an estimate no worse than the *a priori* that the translation rates are constant for all proteins. Crucially, the sign is preserved. The conclusions hold with no loss of generality if the binding is non-saturated, such that $A=\phi np$ with a saturation fraction of $\phi\in\left( 0,1 \right)$, as long as $\phi$ is not excessively small. By the same argument, we posit that *any* modest bias in the protein counts is insubstantial. For example, if fraction $\varphi$ of proteins is retained in the cell and thus not observable, $A=\varphi\phi np$ and $t$ must be expressed in units of $\frac{\beta_{p}}{\varphi\phi n}$. As long as $\varphi\phi n$ is $O(1)$, the error of this estimate is still $O(\beta_{p})$, no worse than the original estimate. For the purposes of our analysis, we assume $\varphi\phi n=1$.

Since both splicing (2) and translation (3) are very tightly regulated in eukaryotes, the assumption of constant rates across all cells and genes means the extrapolation step is qualitative. The quality of this assumption depends upon the underlying distribution of splicing and translation rates. Previous scholarship has found splicing timescales that vary over several orders of magnitude; estimates substantially depend on the methodology (1,4). We can use the translation initiation rate as an estimate of the translation rate in our first-order model.

Transcriptome-wide measurements of per-mRNA eukaryotic initiation rates are not readily available. However, they may be estimated from auxiliary data. Initiation rates are proportional to the ribosome density and inversely proportional to the speed of elongation (5). Since the elongation speed per codon is quite narrowly distributed (5), we use the ribosome density as a proxy for the distribution of initiation rates. Specifically, we calculate the differences in expression between ribosome-protected and control samples. The ribosome profiling expression per unit length is determined by underlying expression per unit length and the ribosome density; by normalizing the ribosome-protected expression by the total expression, we estimate the ribosome density. We follow Siwiak and Zielenkiewicz (5) in using HeLa ribosome profiling expression data from Guo et al. (6), and follow Guo et al. in the interpretation of normalized expression as a reflection of ribosome density.

The resulting estimates, shown in **Fig S44**, suggest that the expression change for the cluster of differentiation genes is distributed within approximately one order of magnitude (std of log fold change $= 0.251$), compared to three orders of magnitude (std of log fold change $= 0.428$) for the entire transcriptome. Therefore, assuming similar translation rates for the cell surface markers used in the feature barcoding technologies appears to be no more problematic than assuming similar splicing rates for several thousand genes in the transcriptome. We discuss the implications of these uncertainties, and outline potential directions for improving the embedding process and removing assumptions in the section “**Methodological directions, quantitative and qualitative**” below.

The low dimensionality of the protein data may amplify deviations. The two resulting ODEs are normalized to different parameters, but we do not weigh the extrapolation results differently when constructing the past and future estimates. For qualitative purposes, the timescales of translation and splicing are comparable. From mean estimates for human genes, we take the translation initiation time to be $7$ s and the elongation time per codon to be $87$ ms (5). For the seven proteins used for protein velocity estimation in the CITE-seq dataset (7), the expected average translation timescale is $0.5-1$ min. Sources are equivocal on the rate of splicing; recent estimates yield $0.5-5$ min (4), comparable to translation.

Violations of the assumption of unity $\beta$ and $\beta_{p}$ make quantitative comparisons of velocity between different genes problematic. However, crucially, we can predict the direction of each velocity, since both $\beta$ and $\beta_{p}$ are positive in the physiology model. We discuss potential routes to harnessing the direction directly in the section “**Methodological directions, quantitative and qualitative**.”

We do not *a priori* assume that protein degradation rates are constant. We adopt an approach analogous to RNA velocity. Gene/protein pairs that have phase portraits with an appearance apparently inconsistent with a single constant degradation rate (i.e. no evident linear component) are omitted from further analysis. The sole difference is that our selection process is manual; this is practical for the low-dimensional feature barcoding datasets available at this time.

The extrapolation process is likewise analogous; however, we perform smoothing using over nearest neighbors in the normalized protein space rather than the normalized spliced molecule space. Our approach appears to be more robust due to the lower dimensionality of the protein space. Neighbor determination requires cell distance calculation, which is problematic in high dimensions due to the “curse of dimensionality.”

Finally, the embedding process closely follows and extends the assumptions of RNA velocity. Crucially, we still assume cell-cell transitions only occur to embedding neighbors, and probability of transition is determined by the correlation between the velocity direction and the directions to the neighbors in the high-dimensional space. The extension we adopt is that the protein embedding correlation is calculated using values in the *protein* space.

## Validation

To validate the inference and extrapolation procedures, we used stochastic simulations to generate ground truth data for the physiology described in **Figure 1**. The simulation algorithm was adapted from the cornerstone work by Gillespie (8); the specific implementation accompanied a recent preprint (9). To simulate a single gene, we randomly chose a set of model parameters from a physiologically plausible range, simulated the system until a steady state was reached, calculated RNA and protein velocities, then compared them to ground truth information about cell states in the past and present. We repeated this procedure for $6,393$ sets of parameters to provide a qualitative description of regions in parameter space where the inference processes perform best. As a negative control, we also identified the regions in parameter space where a zeroth-order approximation of future and past cell states (i.e., no dynamics) is valid, and the information provided by the velocity estimation procedures is trivial.

For a sample gene that spends a relatively large fraction of its lifetime being transcribed, the estimation of the degradation rate is fairly facile and accurate, as shown in **Fig S45**. Further, the RNA velocity estimate is correlated with the true direction and magnitude of the forward difference in spliced counts, but cannot predict the past cell state. For such a gene, the estimation of the protein degradation rate is less accurate and rather biased toward higher values; however, the prediction quality is largely unaffected and the protein velocity estimate is highly correlated with the backward difference in protein counts. The roughly equal correlation with the forward difference is a consequence of the extremely long protein lifetimes.

For a sample gene that spends a relatively large fraction of its lifetime being transcribed, the estimation of the degradation rate is less accurate, as shown in **Fig S46**. The RNA count sparsity reduces the quality of the forward extrapolation. The estimation of the protein degradation rate shows a significantly higher positive bias. However, this bias still does not seem to significantly affect the correlation between the estimated and ground truth protein velocities. Qualitatively, even if the spliced mRNA counts are extremely sparse, the estimation procedure can nevertheless identify that cells with extremely high protein counts will undergo protein depletion in proportion to the amount of protein. We note that we expect the bias to be attenuated in the physiological dataset processing due to the k-nearest neighbor pooling process; we do not use pooling here due to the independence of randomly sampled genes.

The results of these analyses for a broad range of parameters are shown in **Fig S47**. The RNA velocity parameter estimation process does not appear to show a significant bias. The corresponding forward extrapolation process loses effectiveness as the burst sizes decrease due to low molecule counts. Further, in the domain of low burst sizes and high degradation rates, RNA velocity provides scant information over the zeroth-order approximation of constant spliced counts. On the other hand, protein velocity parameter estimation does show a positive bias at very low degradation rates, already described above. However, this bias does not seem to substantially impact the extrapolation quality at the low degradation rates. As may be expected, *high* degradation rates deplete molecule counts with information about past cell states and reduce the effectiveness of predictions. Finally, the region with joint high protein and RNA degradation rates exhibits static behavior.

Qualitatively, the physiologically realistic parameter space, denoted by a magenta box, exhibits fairly good extrapolation performance for both RNA and protein velocities. The distributions of correlations within each respective region are shown in **Fig S48**. The mean correlation between RNA velocity and the forward difference in spliced counts is $0.57$, with an empirical $90\%$ confidence interval of $(0.29, 0.83)$. The mean correlation between protein velocity and the backward difference in protein counts is $0.66$, with an empirical $90\%$ confidence interval of $(0.30, 0.94)$. This analysis demonstrates that the performance of protein velocity estimation is no worse, and often better, than that of RNA velocity within the space of parameters expected in living systems.

### Model description and physiological parameter selection

The parametrization of the model, as well as the range of parameter choices sampled, require dedicated discussion, as they differ somewhat from that adopted by La Manno et al. (1). Transcriptome-wide studies using a variety of technologies show that transcriptional kinetics are best described by a stochastic model with infrequent bursts of transcription (10–12). Therefore, instead of implementing a continuously varying transcription rate and performing validation on time-series data in the vein of the original RNA velocity publication (1), we simulate an *ensemble* of cells until they reach steady state, then perform inference from the resulting steady-state mRNA and protein counts.

The stochastic model is shown in **Fig S49**. In summary, a gene exists in one of two states, on or off. A gene turns on at the rate $k_{on}$ and turns off at the rate $k_{off}$. In the on state, it can produce unspliced mRNA transcripts ($u$) at a rate of $k_{ini}$. The unspliced transcripts isomerize to spliced transcripts ($s$) via a first-order reaction with a rate of $\beta u$. The spliced transcripts can be degraded by a first-order reaction with a rate of $\gamma s$ or translated into protein ($p$) by a first-order of reaction with a rate of $\beta_{p}s$. The proteins are degraded by a first-order reaction with a rate of $\gamma_{p}p$. This model is simple, but fairly standard throughout the gene expression analysis literature (13). We extend the existing models, which tend not to distinguish spliced and unspliced RNA, by incorporating the first-order splicing reaction. This mathematical formulation assumes that spliceosomes are abundant and splicing rates are strictly controlled by the amount of unspliced RNA in the system. This assumption is consistent with the other assumptions underpinning the first-order reaction schema, i.e. the abundance of ribosomes, ribonuclease, and protease. We do not model delays in transcription, splicing, or translation.

The simulation of a single gene requires the seven parameters $k_{on}, k_{off}, k_{ini}, \beta,\gamma,\beta_{p}, \gamma_{p}$ depicted in **Fig S49**. However, for the purposes of investigating steady-state behavior, the units of the timescale are not relevant, and we can express all parameters in terms of $\beta$ with no loss of generality. This normalization reduces the parameter space to six dimensions.

The physiologically plausible ranges of parameters, as well as ranges actually used in simulations, are provided in **Table S4**. The determination of all of these parameters from a single technology is infeasible; we aggregated a series of studies to establish a physiologically plausible range.

The RNA velocity study (1) estimated the RNA splicing and degradation rates across the transcriptome using 4-thiouridine labeling in human cells (HEK293 cell line). The range of RNA splicing rates ($\beta$) was found to be $0.5-64$ min^-1^ (from $832$ genes), with genes predominantly lying in the $\beta\in(2,4)$ min^-1^ range. For convenience, we set $\beta$ to $2$ min^-1^ for all genes. The range of observed RNA degradation rates ($\gamma)$ was $0.125-8$ min^-1^.

A compilation of translation initiation times in humans found that the mean transcript ribosome binding time $T_{\beta p}$ was $7$ s, with minimum and maximum observed times of $1$ and $1372$ s (from $7494$ genes) (5). We estimated the physiologically relevant range of times by finding the geometric means between the mean and extrema, yielding an interval of $2.65-98$ s or $0.0441-1.63$ min. Finally, we estimated the translation rate per molecule of spliced RNA as $\beta_{p}=1/T_{\beta p}$, yielding a $\beta_{p}$ range of $0.61-23$ min^-1^ spliced RNA^-1^.

A recent study quantified degradation rates for a broad panel of glycoproteins (14). We judged these proteins to be most relevant to the current investigation of cell surface protein kinetics. The $10\%-90\%$ quantile range for the subset of glycoproteins identified as clusters of differentiation (CD) was $0.0063-0.0393$ hr^-1^ (from $14$ proteins). As a control, we calculated the corresponding quantile range for the entire dataset; this range was broader at $0.0064-0.0294$ hr^-1^ (from $625$ proteins). We used the broader range calculated from the CD proteins, $\gamma_{p}\in(1.05, 6.55)\times{10}^{-4}$ min^-1^ protein^-1^.

We estimated gene expression parameters from a transcriptome-wide study (12). The burst sizes ${b=k}_{ini}/k_{off}$ quantified in the study were between $100$ and $300$ mRNA. The burst frequencies $k_{on}$ were in the range $0.003-0.01$ min^-1^. For consistency with the studies that reported bursty transcription in eukaryotes (8–10) , we assumed the gene-specific on fractions $P_{on}=k_{on}/(k_{on}+k_{off})$ were distributed between $0.01$ and $0.2$. For a given $P_{on}$, $k_{off}=\left( P_{on}^{-1}-1 \right)k_{on}$, giving a $k_{off}$ range of $0.003-0.99$ min^-1^. To estimate the $k_{ini}$ region, we calculated the range of $bk_{off}$, yielding $0.3-297$ min^-1^.

Given these parameter ranges in units of min^-1^ (**Table S4**, column 2), we normalized them to the chosen value of $\beta=2$ and calculated their base-10 logarithm, yielding **Table S4**, column 4. To initialize a simulation with a parameter set, we sampled from a uniform distribution on the parameter range in logarithmic space, albeit with several modifications to facilitate simulation and exploration of a broader parameter space. Firstly, we truncated the lower logarithmic bound for $k_{ini}$ to $-0.22$, as our primary interest lies in genes with relatively high expression per burst. Secondly, we relaxed the upper logarithmic bound for $\gamma_{p}$ to $-2.48$ to explore the failure modes of extrapolation at relatively high protein degradation rates. Finally, we did not sample $k_{off}$ from the entire distribution; for each simulated gene, we set the minimum $k_{off}$ to the value of $k_{on}$, giving a relaxed $P_{on}$ maximum of $0.5$.

We simulated the stochastic model for an ensemble of $500$ cells until steady state. The characteristic relaxation time to reach steady state was estimated as $T=5/\min(k)$, with $k$ being the vector of all kinetic parameters; thus, the slowest timescale determined the relaxation time. We stored cell observations ($s,u,p$) at three times, separated by a *characteristic forecast time* $\tau$. Since extrapolation is meaningful on timescales where the molecule population is retained, i.e. the respective degradation rates, we chose $\tau$ to be the smaller of $1/\gamma$ and $1/\gamma_{p}$. For the long-lived glycoproteins of interest in this study, $\tau$ was always $1/\gamma$.

In the interest of testing the applicability of the protein acceleration workflow to future datasets that may incorporate soluble proteins with lifetimes comparable to mRNA, we tested the range of synthetic parameters given in **Table S4**, column 5. Further, we relaxed the $k_{on}$ range. Typical performance for high $k_{on}$ and $\gamma_{p}$ is demonstrated in **Fig S50**. The striking characteristic phase portraits typically used for RNA velocity are fully recapitulated at high $k_{on}$. Further, a relatively high $\gamma_{p}$ allows the protein levels to fairly closely track the RNA levels, while still retaining information about the past cell state. Simulations across a large parameter region with $k_{on}$ and $\gamma_{p}$ show fairly high performance throughout, as shown in **Fig S51**. As expected, excessively high $\gamma_{p}$ precludes any extrapolation due to the rapid loss of information from proteins. Further, consistently with the other simulations, the joint high $\gamma$ and $\gamma_{p}$ region exhibits very sparse dynamics. This investigation suggests that the oval phase portraits of RNA velocity are characteristic of high $k_{on}$. However, accurate extrapolation is possible far outside of that domain, as long as the degradation rates are not excessively high. This robustness makes us confident that protein acceleration will be applicable to future feature barcoding datasets with the capability to quantify short-lived proteins.

### Determination of previously validated parameter regions

The model differences between the deterministic transcriptional modulation used by La Manno et al. and stochastic gene switching used here make a direct comparison to the validation performed in the RNA velocity publication challenging (1). The deterministic model includes a transcriptional ramp-up, plateau, and ramp-down. Slow ramp-up represents a region of poor performance for parameter determination, as the gene equilibrates before unsteady dynamics can be observed. Instantaneous discrete switching on is the limiting case of very fast ramp-up. Ramp-down duration is fairly short compared to plateau width; its limiting case reduces to discrete switching off. Therefore, our stochastic model is consistent with the regime that experiencing high-quality performance in the continuous model.

From Fig. 5 of SI2 (1), the $90\%$ interval of plateau width $\tau_{on}=1/k_{off}$ is roughly $2-12$. The interval for plateau scale $\alpha$ is $0.5-7.5$. Minimum burst size is $\alpha_{min}\tau_{min}=1$. Maximum burst size is $\alpha_{max}\tau_{max}=90$. Therefore, the range of burst sizes explored in the original validation is roughly $1-2$ orders of magnitude below the physiologically observed range. The scale of $\gamma$ appears to have a $90\%$ interval of $0.1-2$. The corresponding scale of $\beta$ is distributed in $0.4-1.9$. The normalized degradation rate is distributed between $\frac{\gamma_{min}}{\beta_{max}}=0.0526$ and $\frac{\gamma_{max}}{\beta_{min}}=5$. These ranges are fairly consistent with physiologically plausible ranges. The comparison between the physiologically relevant parameter domain (magenta box) and the previously explored parameter domain (black dotted box) is visualized in **Fig S47**.

## Methodological directions, quantitative and qualitative

To identify viable improvements to the biological relevance of RNA and protein velocities, it is necessary to distinguish independent components of the workflow. The velocity estimation front-end requires *biophysical extrapolation* and *embedding*. The former process consists of the estimation of $\gamma$ and the consequent calculation of $\frac{1}{\beta}\frac{ds}{dt}$; in broad strokes, it is consistent with the methodology of previous single-cell biophysical scholarship (15). The latter process consists of the calculation of a transition matrix and the determination of a low-dimensional embedding of cell dynamics. Ubiquitous embeddings are designed to optimize global or local metrics measuring the concordance between data and low-dimensional representation. This objective may not necessarily match the requirements of dynamics embedding. In fact, the original publication describing RNA velocity found that the procedure has significant biases (e.g. (1), SI2, Figs. 19-20).

We anticipate that the assumptions of identical splicing rates and identical translation rates across all genes may contribute to error in the embedding step. Previous scholarship has found splicing timescales that vary over several orders of magnitude; estimates substantially depend on the methodology (1,4). In the first-order model of translation we use in the current method, the total synthesis rate of a protein linearly scales with the amount of protein-coding mRNA. We estimated the distribution of underlying per-mRNA translation rates for CD glycoproteins by leveraging ribosome profiling data (6). We found that the expression changes due to ribosome protection, proxy measurements for ribosome densities and thus translation initiation rates, were distributed within about an order of magnitude for this subset of proteins.

These deviations from unity translation rates are comparable to the deviations from unity splicing rates, but both are potentially problematic. In an intuitive sense, the rates serve as weights for the vector entries used to calculate velocity-displacement correlations in the transition matrix. The assumption of unity rates corresponds to an unweighted calculation. There are essentially three methods for eliminating this assumption and introducing relative timescales in spite of the intrinsically atemporal nature of the scRNA-seq data:

1. Weighing the velocity vector by orthogonally or independently determined estimates of rates.
2. Eliminating the quantitative correlation altogether and operating on a *binary* measure of concordance between velocity and direction.
3. Adding prior information about relevant developmental timescales and globally estimating gene-specific rates.

The first approach requires estimates of splicing and translation rates from an orthogonal technology. For example, thiol labeling can determine timescales for splicing over the course of a single-cell sequencing experiment. Although currently available datasets do not have translation rates for the protein targets accessible by feature barcoding technologies – for example, the CD proteins quantified in recent studies (14,16) have essentially no overlap with the surface proteins investigated by CITE-seq, REAP-seq, ECCITE-seq, or 10X Feature Barcoding – we anticipate that the future compilation of estimates for these rates will improve the embedding process. To facilitate this approach, the *protaccel* package can utilize user-defined relative splicing and translation rates to compute the embedding.

The second approach does not require any external information, and simply uses the *signs* of velocities and displacements to estimate the transition matrix. The fraction of concordant signs yields a distance metric that is directly used to compute the transition kernel. Since the reaction rates are positive, this metric is invariant with their scaling. We have found that the RNA velocity landscapes are fairly well reproduced through this method (**Fig S52**, cf. La Manno 2018, Fig. 4a). Our preliminary investigations have been able to recapitulate significant qualitative features of the protein acceleration landscape (**Fig S53**, cf. **Fig S54**). However, the sparsity of available data – at most thousands of cells with at most dozens of protein markers – makes this method rather sensitive. Further, we expect that the lack of quantitative agreement is related to the edge effects characterized by the developers of RNA velocity (e.g. (1), SI2, Fig. 19). We anticipate that future methods with larger sample sizes and proteomic panels will improve this embedding process. The further development of binary correlation metrics is particularly valuable because of its ability to sidestep the $O(1)$ corrections necessary to quantitatively account for stoichiometry, localization, and binding saturation. To facilitate the study of this approach, the *protaccel* package can use a binary metric to compute the embedding.

The third approach, used by the recent *scvelo* package (17), performs Bayesian inference to estimate all rate parameters simultaneously, using prior information about the developmental timescale of the differentiation process under investigation to inform the rate estimates. However, this approach is inapplicable if a well-founded timescale is unavailable, if the system is stiff with multiple competing timescales, or if the cells are not expected to undergo differentiation.

### Directions for applications

Despite the fact that both RNA and protein velocity methods are largely qualitative, there is a number of unique motivations for specifically performing protein acceleration analysis on feature barcoding data.

Firstly, the significantly longer timescale of protein degradation allows extrapolation across longer timescales than permitted by RNA velocity. As evident from simulated data in **Fig S45**, **Fig S46**, and **Fig S50**, extrapolation on the timescale of $O(\beta)$ yields fair results for both RNA and protein velocity. However, extending the timescale substantially degrades the quality of RNA velocity extrapolation, whereas protein velocity remains effective, as shown in **Fig S55**. Further, even in the short-time scenario, backward extrapolation based on RNA velocity performs poorly. We anticipate that these precision and quantitative value of these methods will be refined by the development of more physically realistic models and the collection of rate data. However, even in the current state, protein velocity is the sole method suited to hour-scale and backward extrapolation from single-time point data without chemical labeling. Protein velocity invites natural comparisons to chemical labeling data with multi-hour sampling or treatment times. A recent preprint by Cao et al. (18), investigating cell cycle dynamics by thiol labeling, observed fairly substantial differences between trajectories inferred from RNA velocity and from the experiment timing. We expect that part of this discrepancy is attributable to RNA velocity reflecting short-time horizon, “high-frequency” dynamics and the labeling reflecting long-time horizon, “low-frequency” dynamics, and suggest that analysis based on *protein* abundance may be more comparable. More broadly, the extended timescale may be useful for datasets with a sparsely observed cellular landscape, such that the high-frequency RNA velocity and low-frequency neighborhood cease to correlate.

Secondly, in the short-time limit, the availability of a single observed and two extrapolated points lends itself to the description of non-linear behavior. RNA velocity corresponds to a single extrapolation ray, and can most effectively describe linear traversal of a manifold. We postulate that the additional "curvature" information can provide a route toward description and inference of more sophisticated regulation mechanisms and developmental landscapes. This approach has, for example, been adopted by Qiu et al. in a recent preprint using metabolic labeling for cell state vector field reconstruction (19). Qiu et al. use dynamic information from single cells to learn vector fields underlying differentiation processes, and we anticipate that protein acceleration will provide a useful data source for such methodologies. The applications range from quantitative, e.g., directly using the protein velocity information to reconstruct vector fields, to qualitative, e.g., using the sign of the protein velocity to validate findings from RNA-based reconstruction methods.

# References

1. La Manno G, Soldatov R, Zeisel A, Braun E, Hochgerner H, Petukhov V, et al. RNA velocity of single cells. Nature. 2018 Aug;560(7719):494–8.

2. Wang Z, Burge CB. Splicing regulation: From a parts list of regulatory elements to an integrated splicing code. RNA. 2008 Mar 27;14(5):802–13.

3. Kozak M. Regulation of translation via mRNA structure in prokaryotes and eukaryotes. Gene. 2005 Nov;361:13–37.

4. Alpert T, Herzel L, Neugebauer KM. Perfect timing: splicing and transcription rates in living cells: Splicing and transcription rates in living cells. WIREs RNA. 2017 Mar;8(2):e1401.

5. Siwiak M, Zielenkiewicz P. Transimulation - Protein Biosynthesis Web Service. Levy YK, editor. PLoS ONE. 2013 Sep 5;8(9):e73943.

6. Guo H, Ingolia NT, Weissman JS, Bartel DP. Mammalian microRNAs predominantly act to decrease target mRNA levels. Nature. 2010 Aug;466(7308):835–40.

7. Stoeckius M, Hafemeister C, Stephenson W, Houck-Loomis B, Chattopadhyay PK, Swerdlow H, et al. Simultaneous epitope and transcriptome measurement in single cells. Nat Methods. 2017 Sep;14(9):865–8.

8. Gillespie DT. A general method for numerically simulating the stochastic time evolution of coupled chemical reactions. Journal of Computational Physics. 1976 Dec;22(4):403–34.

9. Gorin G, Wang M, Golding I, Xu H. Stochastic simulation platform for visualization and estimation of transcriptional kinetics [Internet]. Biophysics; 2019 Nov [cited 2019 Dec 3]. Available from: http://biorxiv.org/lookup/doi/10.1101/825869

10. Grün D, Kester L, van Oudenaarden A. Validation of noise models for single-cell transcriptomics. Nat Methods. 2014 Jun;11(6):637–40.

11. Erhard F, Baptista MAP, Krammer T, Hennig T, Lange M, Arampatzi P, et al. scSLAM-seq reveals core features of transcription dynamics in single cells. Nature. 2019 Jul;571(7765):419–23.

12. Dar RD, Razooky BS, Singh A, Trimeloni TV, McCollum JM, Cox CD, et al. Transcriptional burst frequency and burst size are equally modulated across the human genome. Proceedings of the National Academy of Sciences. 2012 Oct 23;109(43):17454–9.

13. Shahrezaei V, Swain PS. Analytical distributions for stochastic gene expression. Proceedings of the National Academy of Sciences. 2008 Nov 11;105(45):17256–61.

14. Xiao H, Wu R. Simultaneous Quantitation of Glycoprotein Degradation and Synthesis Rates by Integrating Isotope Labeling, Chemical Enrichment, and Multiplexed Proteomics. Anal Chem. 2017 Oct 3;89(19):10361–7.

15. Munsky B, Li G, Fox ZR, Shepherd DP, Neuert G. Distribution shapes govern the discovery of predictive models for gene regulation. Proc Natl Acad Sci USA. 2018;115(29):7533–8.

16. Li JJ, Bickel PJ, Biggin MD. System wide analyses have underestimated protein abundances and the importance of transcription in mammals. PeerJ. 2014 Feb 27;2:e270.

17. Bergen V, Lange M, Peidli S, Wolf FA, Theis FJ. Generalizing RNA velocity to transient cell states through dynamical modeling [Internet]. Bioinformatics; 2019 Oct [cited 2019 Dec 3]. Available from: http://biorxiv.org/lookup/doi/10.1101/820936

18. Cao J, Zhou W, Steemers F, Trapnell C, Shendure J. Characterizing the temporal dynamics of gene expression in single cells with sci-fate [Internet]. Genomics; 2019 Jun [cited 2019 Aug 23]. Available from: http://biorxiv.org/lookup/doi/10.1101/666081

19. Qiu X, Zhang Y, Yang D, Hosseinzadeh S, Wang L, Yuan R, et al. Mapping Vector Field of Single Cells. bioRxiv. 2019 Jul 9;696724.

20. Peterson VM, Zhang KX, Kumar N, Wong J, Li L, Wilson DC, et al. Multiplexed quantification of proteins and transcripts in single cells. Nat Biotechnol. 2017 Oct;35(10):936–9.

**Supplementary Tables**

| CITE-seq | REAP-seq | ECCITE-seq ctrl | ECCITE-seq CTCL | 10X 1k | 10X 10k |
| --- | --- | --- | --- | --- | --- |
| CD3D | ITGAM | ITGAX | CD4 | CD3D | CD3D |
| CD8A | HLA-DRA | CD2 | CD2 | CD8A | CD8A |
| FCGR3A | CD8A | CD27 | DPP4 | CD14 | NCAM1 |
| CD14 | CD8B | CD28 | CD28 | IL2RA | CD14 |
| ITGAX | IL7R | CD3E | HLA-A | IL7R | IL2RA |
| CD19 | PTPRC | CD5 | CD3E | CD19 | IL7R |
| CD2 | CD28 | SELL | CD5 | TIGIT | CD19 |
|  | PDCD1 | CD7 | SELL |  | TIGIT |
|  | CD27 | CD8A | CD7 |  |  |
|  | CD9 | IL7R | CD8A |  |  |
|  | CD19 | HLA-DRA | IL7R |  |  |
|  | CD40 |  | HLA-DRA |  |  |
|  | CD3D |  |  |  |  |
|  | MS4A1 |  |  |  |  |
|  | CD14 |  |  |  |  |
|  | ICOS |  |  |  |  |

**Table S1**. Genes used for protein velocity estimation.

| CD4+ T | B | Monocytes | NK | CD8+ T |
| --- | --- | --- | --- | --- |
| CD4 | CD45RA | CD11b | CD56 | CD8 |
| CD3 | CD19 | CD11c | CD16 | CD3 |
|  | HLA-DR | CD14 |  |  |
|  |  | HLA-DR |  |  |

**Table S2.** Canonical cell surface markers used for cell type identification (7,20).

| RNA velocity | Protein velocity | Comparison of assumption quality |
| --- | --- | --- |
| Gene-specific capture biases do not occur | ADT-specific capture biases do not occur | **Better** because only one polyA capture site is exposed per ADT; multiple or no polyA capture sites may be viable in unspliced molecules |
| Gene-specific identification biases do not occur | ADT-specific identification biases do not occur | **Better** because the designed barcode alignment problem does not face the challenges of intron identification from incomplete gene annotations and spliced/unspliced ambiguity for exon reads |
| N/A | Constant binding stoichiometry | **Equivalent** to splicing rate assumption; effect of binding stoichiometry is $O(1)$ in translation rate |
| Unity splicing rate | Unity translation rate | **Similar**, as discussed in **Methodological directions, quantitative and qualitative** |
| Constant mRNA degradation rate | Constant protein degradation rate | **Equivalent** due to the identifiability of proteins with degradation rates inconsistent with constant values from protein phase plots |
| Imputation based on distance in S space preserves relevant information | Imputation based on distance in P space  preserves relevant information | **Better** due to higher dynamic range of P (thousands rather than tens of molecules) and lessened curse of dimensionality (tens rather than thousands of genes) |
| Isoforms do not substantially affect analysis | Protein subunits do not substantially affect analysis | **Better** because we observe manually select a pair that shows expected behavior, whereas isoform analysis has not yet been incorporated into RNA velocity |
| RNA velocity can be inferred from subset of genes that exhibit expected behavior | Protein velocity can be inferred from subset of gene/protein pairs that exhibit expected behavior | **Equivalent** |
| Transitions occur to kNN in embedding | Transitions occur to kNN in embedding | **Equivalent** |
| Transition probability scales with alignment of RNA velocity and direction to kNN in S space | Transition probability scales with alignment of protein velocity and direction to kNN in P space | **Equivalent** |
| Embedded RNA velocity arrow direction is more meaningfully interpretable than its magnitude | Embedded protein velocity arrow direction is more meaningfully interpretable than its magnitude | **Equivalent**; we make the additional claim that misalignment between the two reveals additional dynamics. |

**Table S3.** Model assumptions and heuristics used in RNA velocity and protein velocity inference processes.

| **Rate** | **Range / min^-1^** | **Source** | $\boldsymbol{lo}\boldsymbol{g}_{\boldsymbol{10}}$ **range** | $\boldsymbol{lo}\boldsymbol{g}_{\boldsymbol{10}}$ **range used (if different)** | $\boldsymbol{lo}\boldsymbol{g}_{\boldsymbol{10}}$ **range used for short-lived proteins** |
| --- | --- | --- | --- | --- | --- |
| $\boldsymbol{\beta}$ | $(0.5, 64)$ | La Manno (1) | $0$ |  | $0$ |
| $\boldsymbol{P}_{\boldsymbol{on}}$ | $(0.01, 0.2)$ |  |  |  |  |
| $\boldsymbol{k}_{\boldsymbol{ini}}$ | $(0.3, 297)$ | Dar (12) | $(-0.82, 2.17)$ | $(-0.22, 2.17)$ | $(0.5, 2)$ |
| $\boldsymbol{k}_{\boldsymbol{on}}$ | $(3 \times{10}^{-3},{10}^{-2})$ | Dar (12) | $(-2.82,-2.30)$ |  | $(-2, -0.5)$ |
| $\boldsymbol{k}_{\boldsymbol{off}}$ | $(0.3, 0.99)$ | Dar (12) | $(-2.82,-0.30)$ | $(k_{on},-0.30)$ | $(k_{on}, -0.5)$ |
| $\boldsymbol{\gamma}$ | $(0.125, 8)$ | La Manno (1) | $(-1.20, 0.60)$ |  | $(-1, 1)$ |
| $\boldsymbol{\beta}_{\boldsymbol{p}}$ | $(0.612, 23.1)$ | Siwiak (5) | $(-0.51, 1.06)$ |  | $(-1, 2)$ |
| $\boldsymbol{\gamma}_{\boldsymbol{p}}$ | $(1.05, 6.55)\times{10}^{-4}$ | Xiao (14) | $(-4.28,-3.48)$ | $(-4.28,-2.48)$ | $(-2, 1)$ |

**Table S4.** Range of model parameters used in validation.

# Supplementary Figures

## Protein velocity phase plots


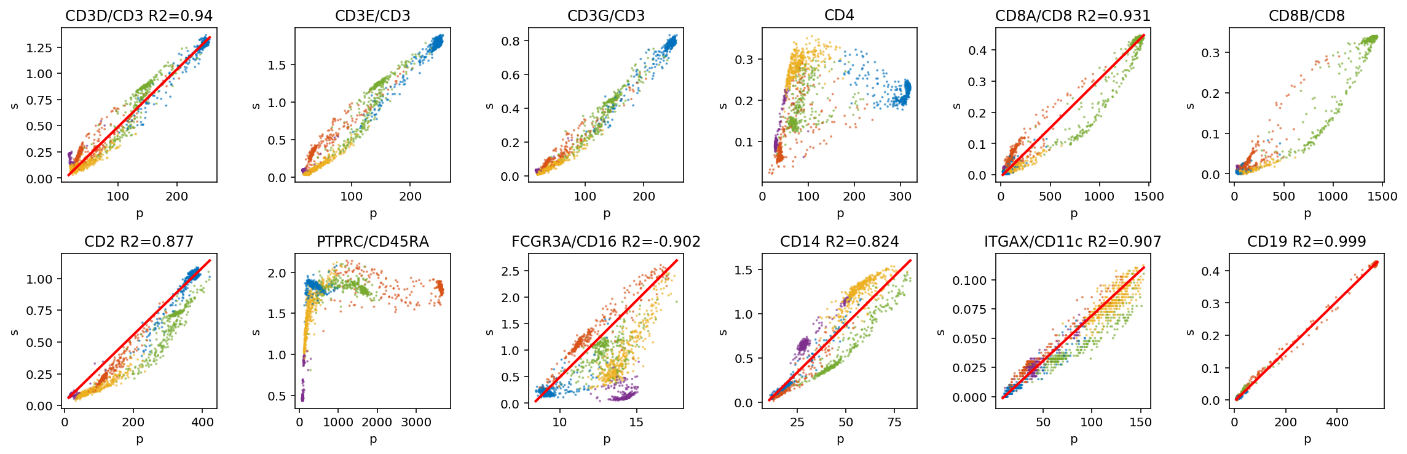


**Fig S1.** CITE-seq imputed RNA/protein phase plots. Pairs used for protein velocity estimation include linear $\gamma_{p}$ fit (red line) and fit $R^{2}$ in title. Color identifies cell type (blue: CD4+ T, red: B, yellow: monocytes, green: CD8+ T, purple: natural killer).


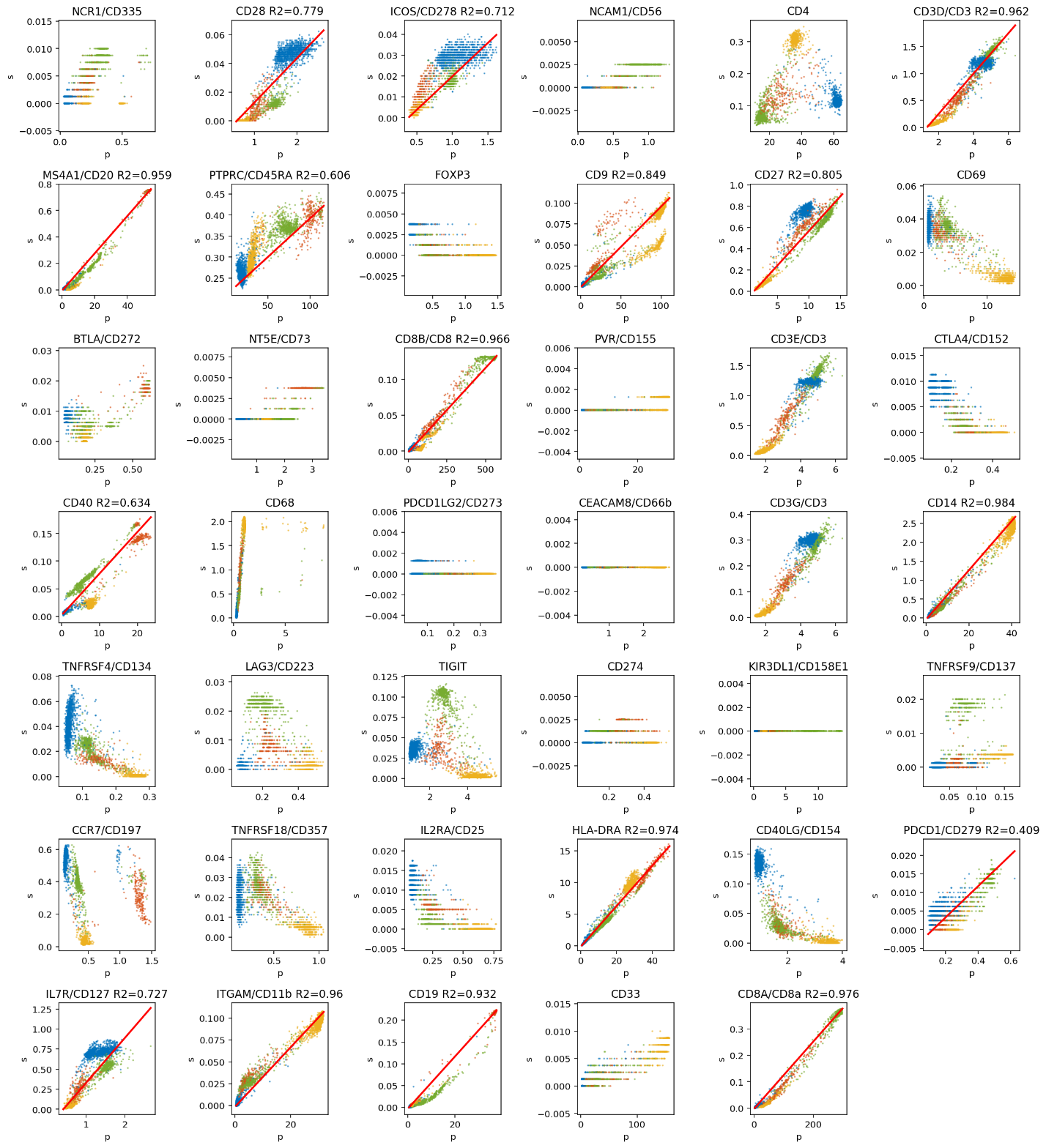


**Fig S2.** REAP-seq imputed RNA/protein phase plots. Pairs used for protein velocity estimation include linear $\gamma_{p}$ fit (red line) and fit $R^{2}$ in title. Color identifies cell type (blue: CD4+ T, red: B, yellow: monocytes, green: CD8+ T).


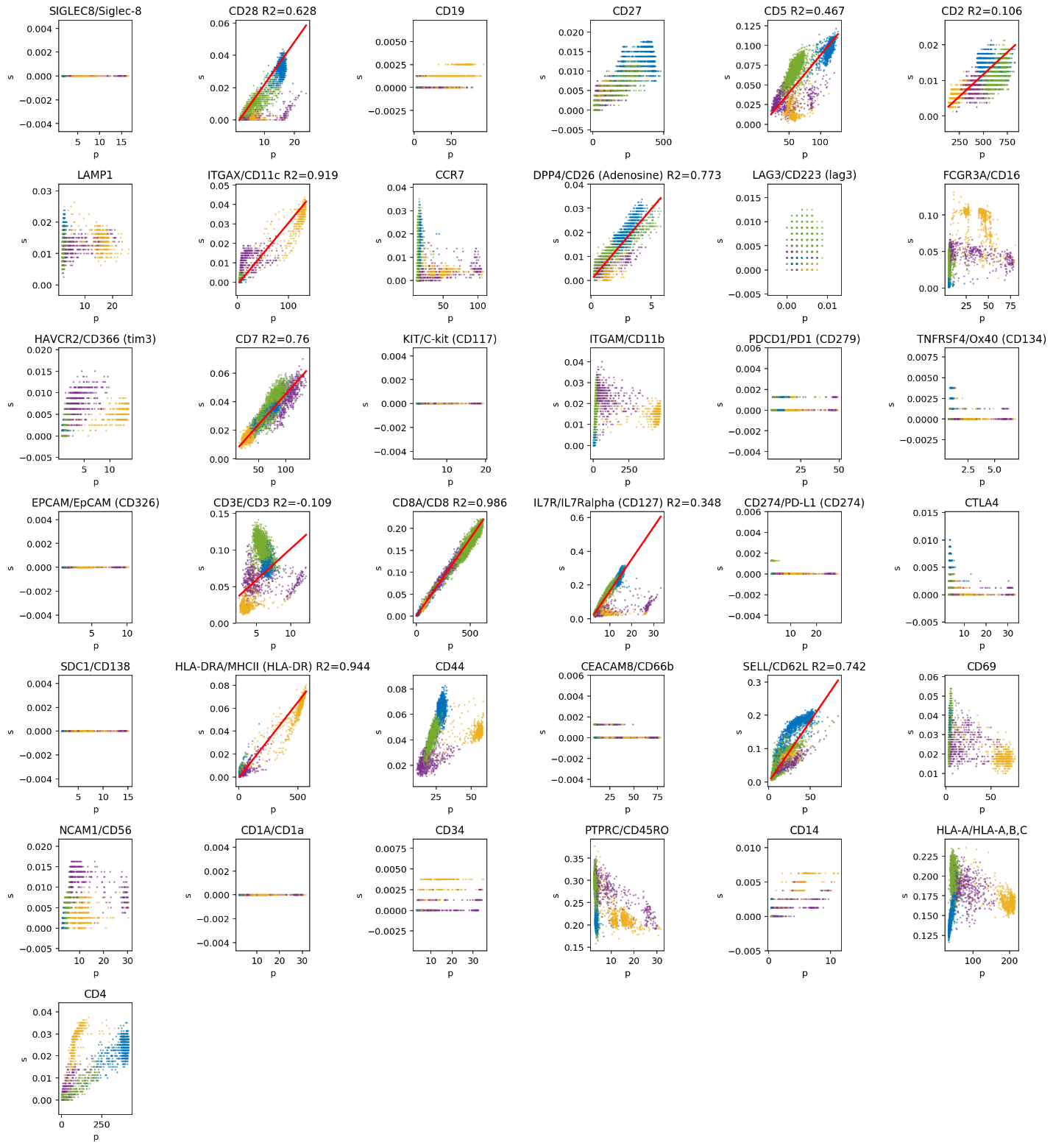


**Fig S3.** ECCITE-seq ctrl imputed RNA/protein phase plots. Pairs used for protein velocity estimation include linear $\gamma_{p}$ fit (red line) and fit $R^{2}$ in title. Color identifies cell type (blue: CD4+ T, yellow: monocytes, green: CD8+ T, purple: natural killer).


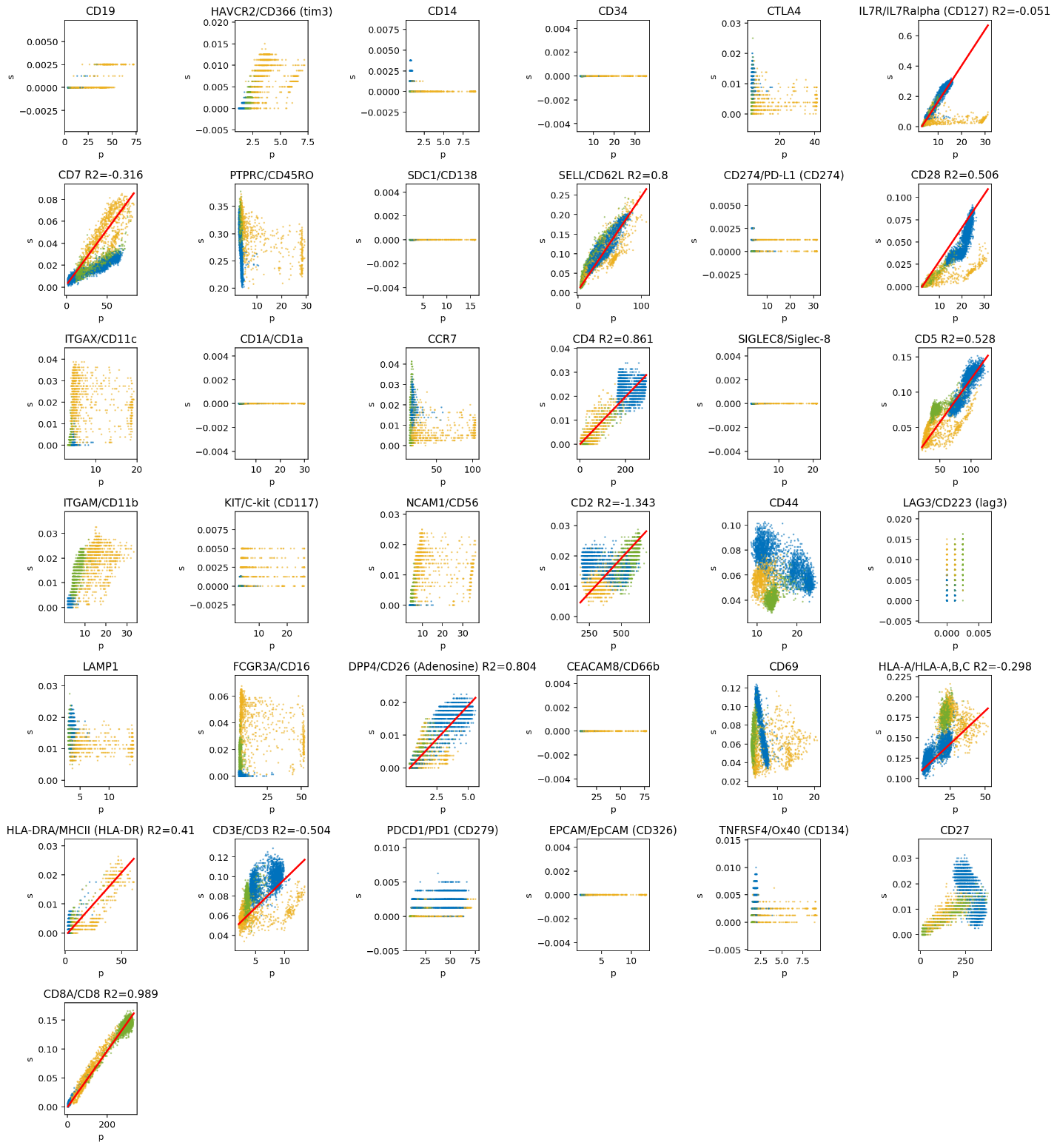


**Fig S4.** ECCITE-seq CTCL imputed RNA/protein phase plots. Pairs used for protein velocity estimation include linear $\gamma_{p}$ fit (red line) and fit $R^{2}$ in title. Color identifies cell type (blue: CD4+ T, yellow: monocytes, green: CD8+ T).


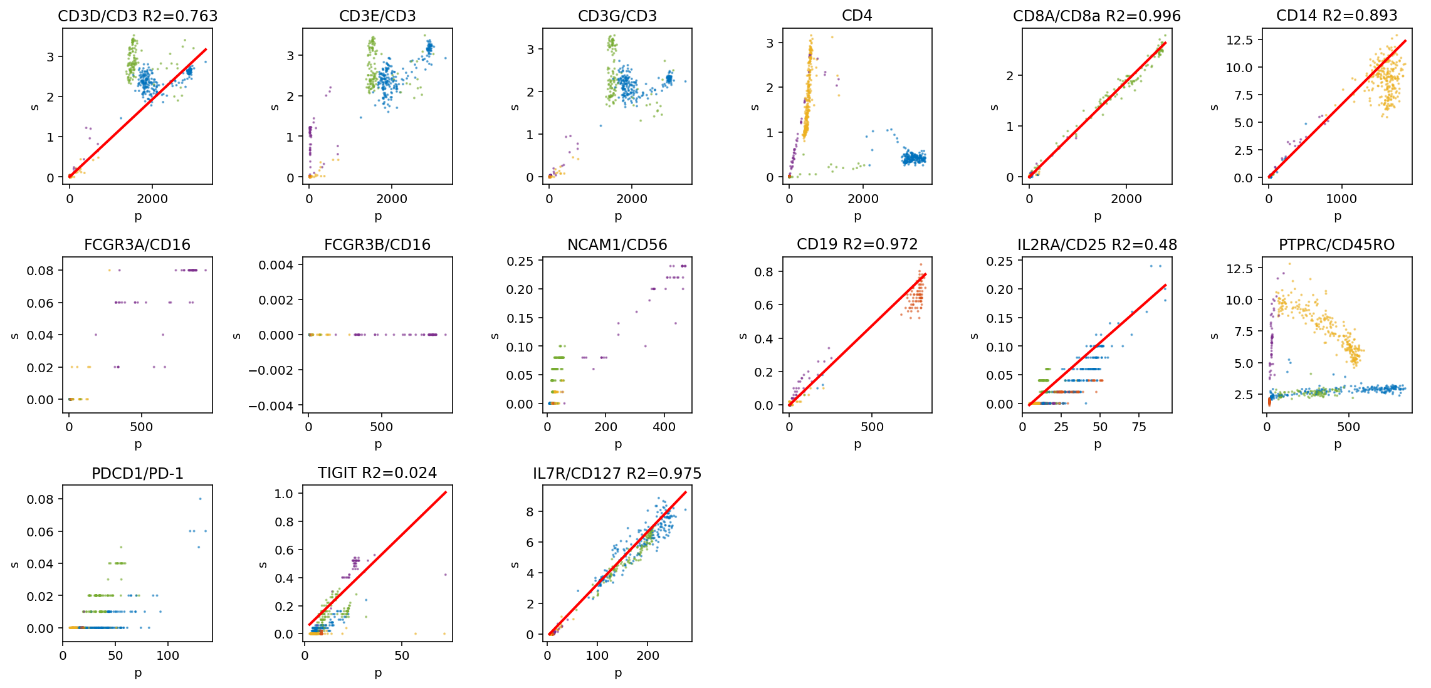


**Fig S5.** 10X 1k imputed RNA/protein phase plots. Pairs used for protein velocity estimation include linear $\gamma_{p}$ fit (red line) and fit $R^{2}$ in title. Color identifies cell type (blue: CD4+ T, yellow: monocytes, green: CD8+ T).


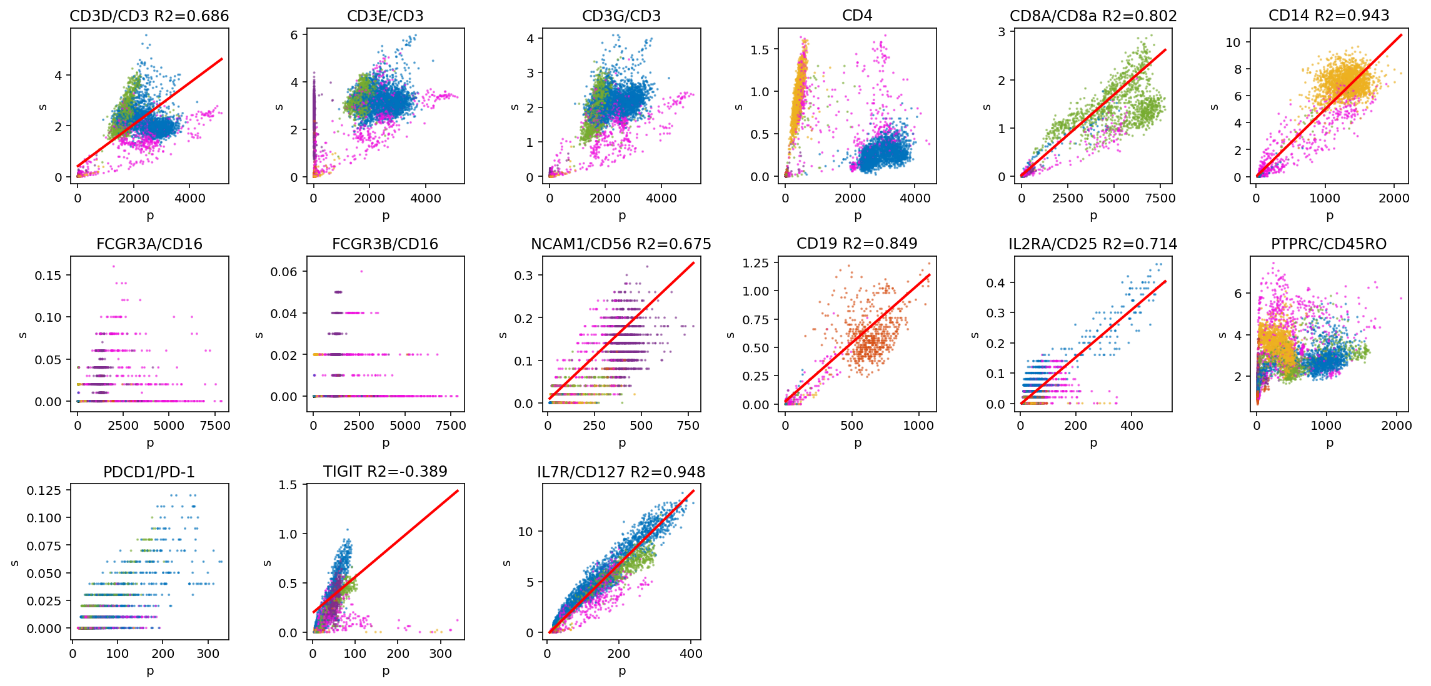


**Fig S6.** 10X 10k imputed RNA/protein phase plots. Pairs used for protein velocity estimation include linear $\gamma_{p}$ fit (red line) and fit $R^{2}$ in title. Color identifies cell type (blue: CD4+ T, red: B, yellow: monocytes, green: CD8+ T, pink: not identifiable unambiguously).

## RNA velocity phase plots


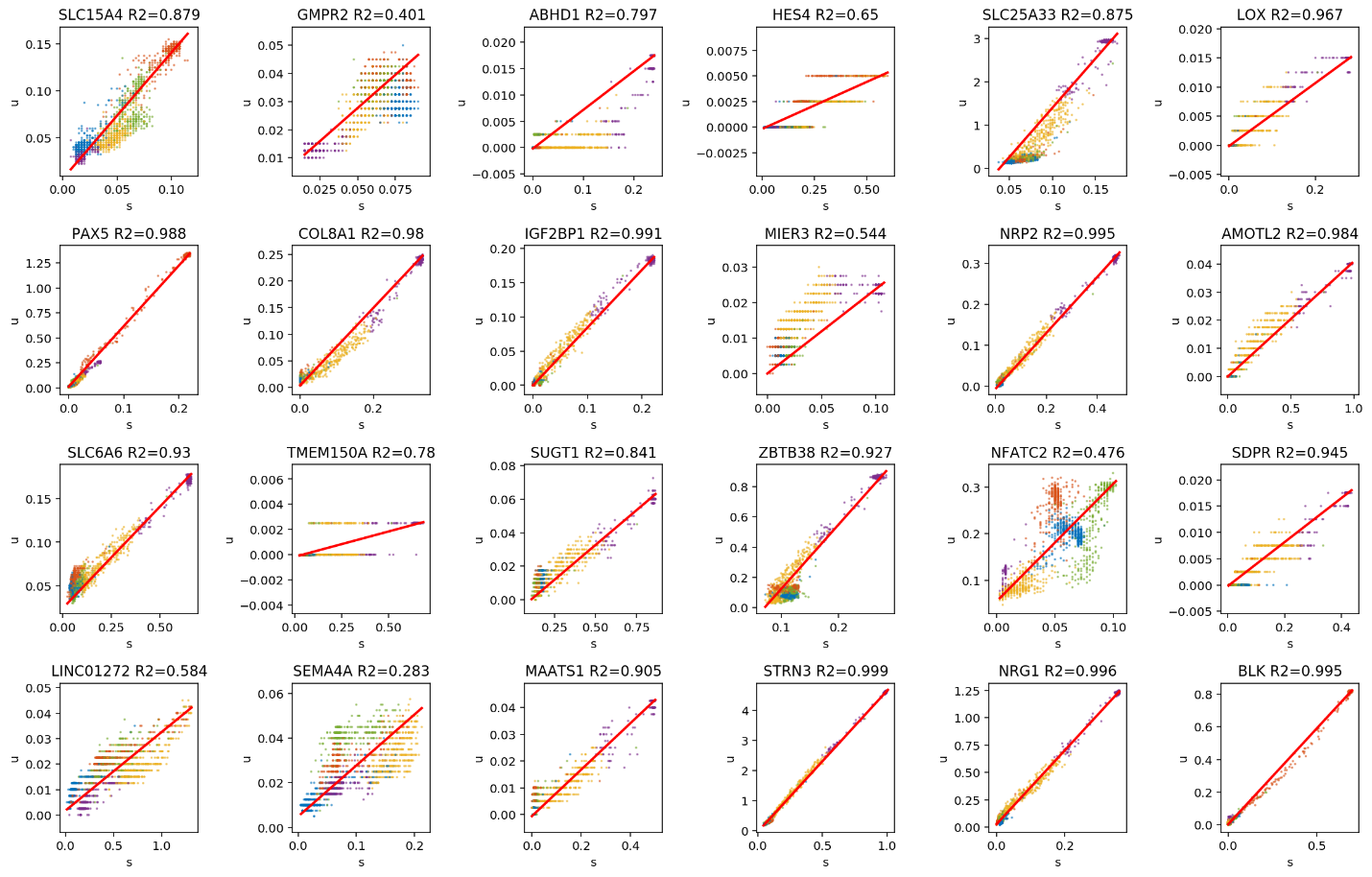


**Fig S7.** CITE-seq imputed spliced/unspliced RNA phase plots. 24 random phase plots out of 1172 genes used to calculate RNA velocity are shown. Linear $\gamma$ fit (red line) is plotted and fit $R^{2}$ is given in title. Color identifies cell type (blue: CD4+ T, red: B, yellow: monocytes, green: CD8+ T, purple: natural killer).


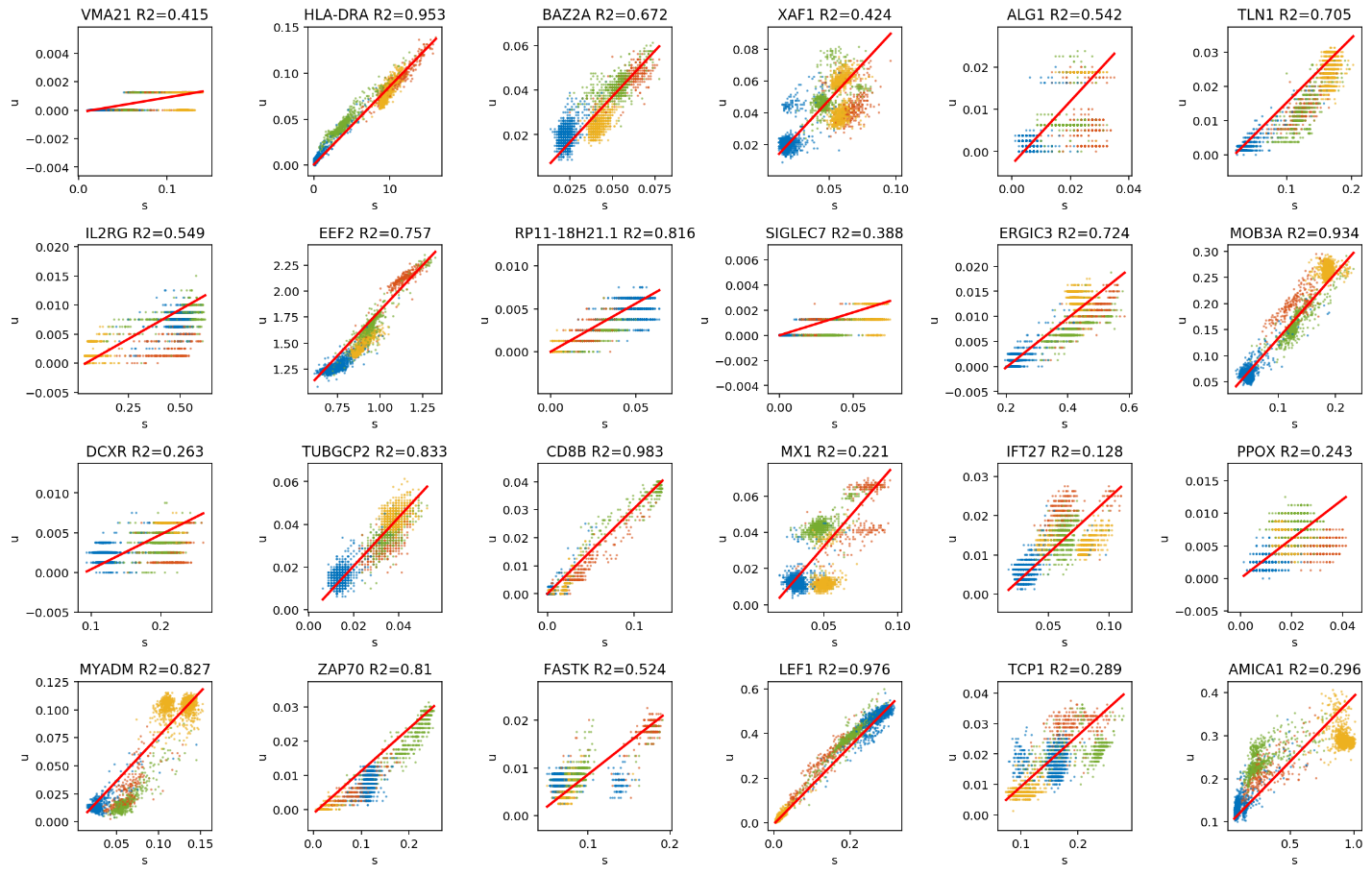


**Fig S8.** REAP-seq imputed spliced/unspliced RNA phase plots. 24 random phase plots out of 1338 genes used to calculate RNA velocity are shown. Linear $\gamma$ fit (red line) is plotted and fit $R^{2}$ is given in title. Color identifies cell type (blue: CD4+ T, red: B, yellow: monocytes, green: CD8+ T).


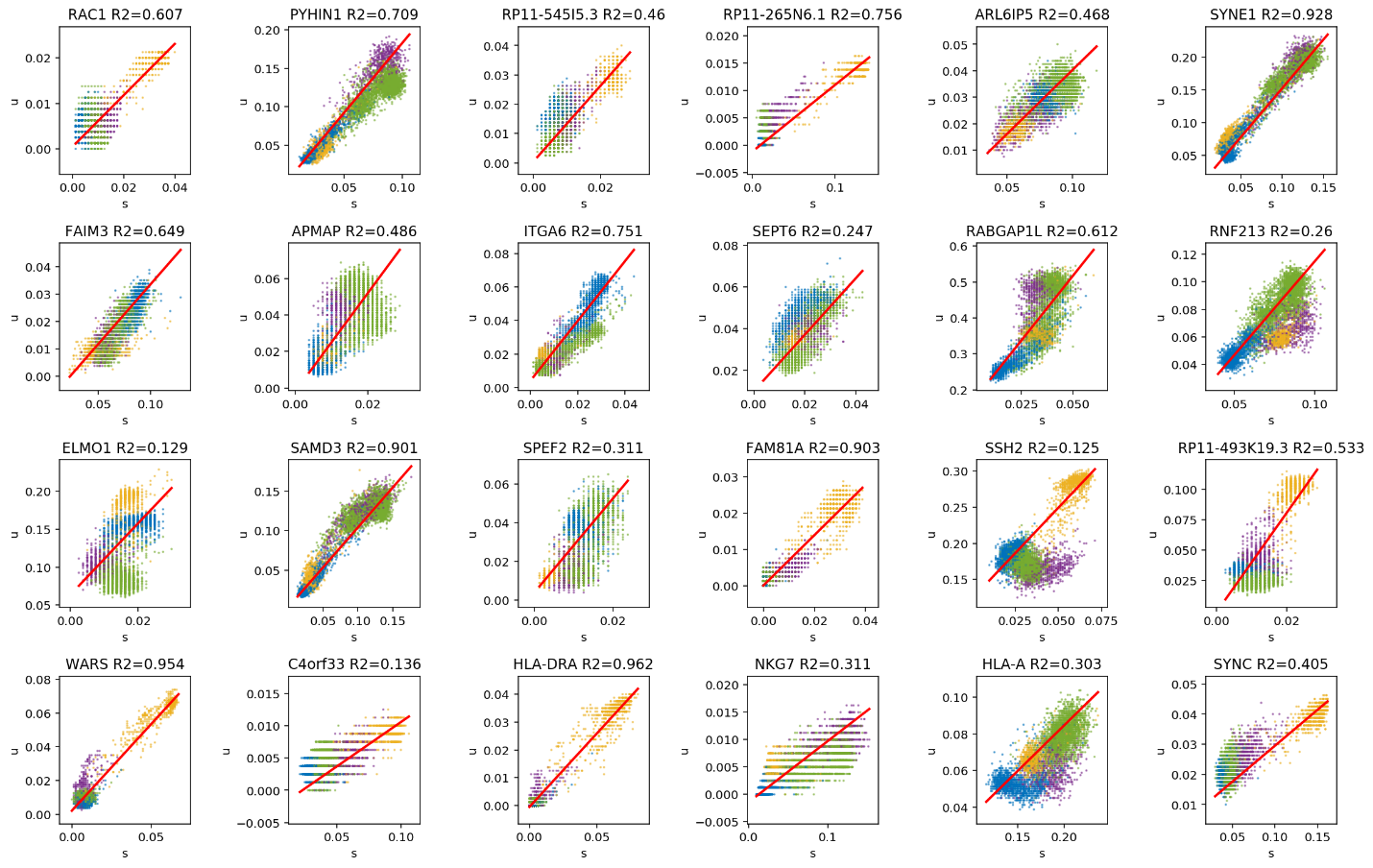


**Fig S9.** ECCITE-seq ctrl imputed spliced/unspliced RNA phase plots. 24 random phase plots out of 591 genes used to calculate RNA velocity are shown. Linear $\gamma$ fit (red line) is plotted and fit $R^{2}$ is given in title. Color identifies cell type (blue: CD4+ T, yellow: monocytes, green: CD8+ T, purple: natural killer).


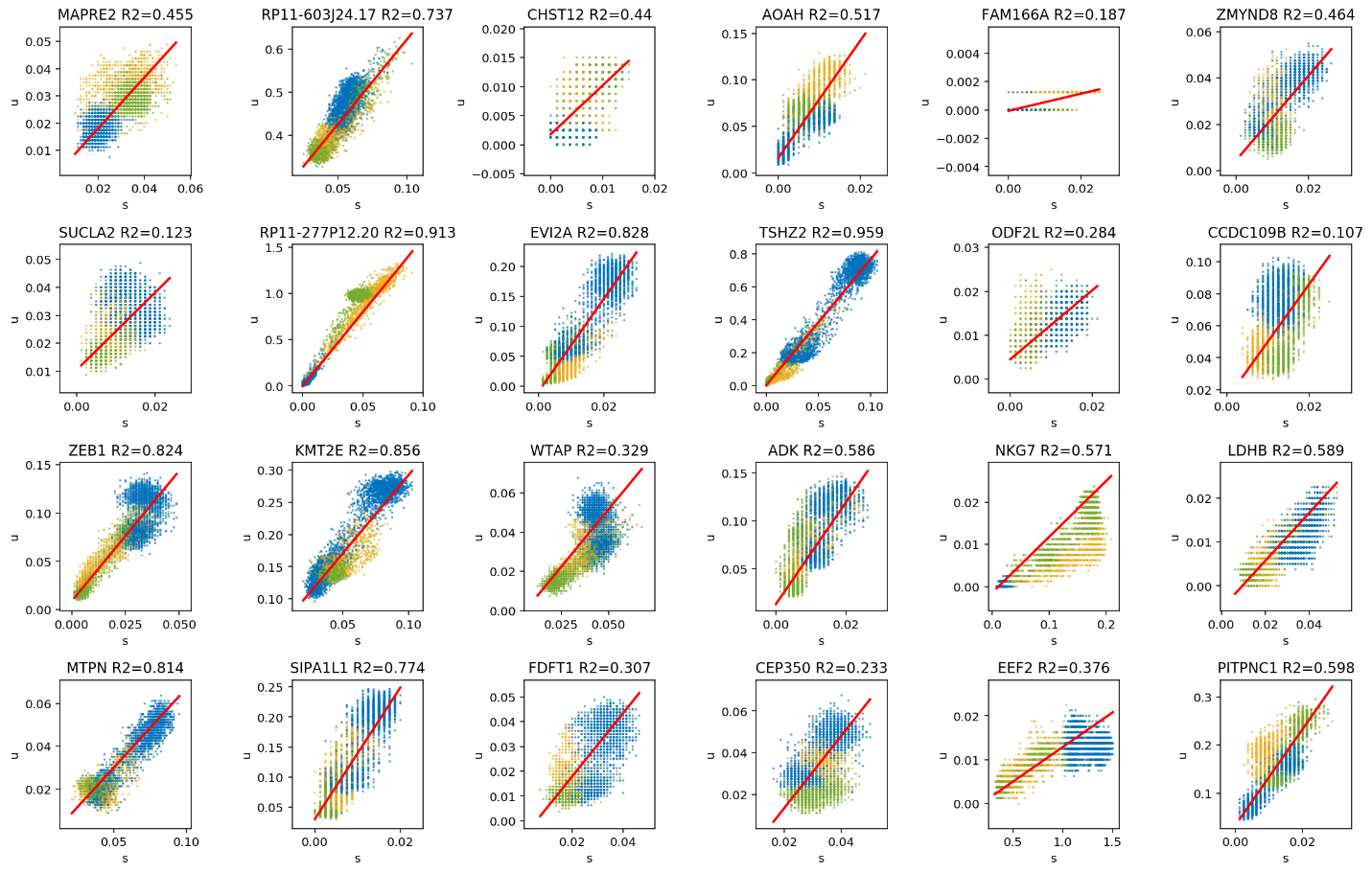


**Fig S10.** ECCITE-seq CTCL imputed spliced/unspliced RNA phase plots. 24 random phase plots out of 667 genes used to calculate RNA velocity are shown. Linear $\gamma$ fit (red line) is plotted and fit $R^{2}$ is given in title. Color identifies cell type (blue: CD4+ T, yellow: monocytes, green: CD8+ T).


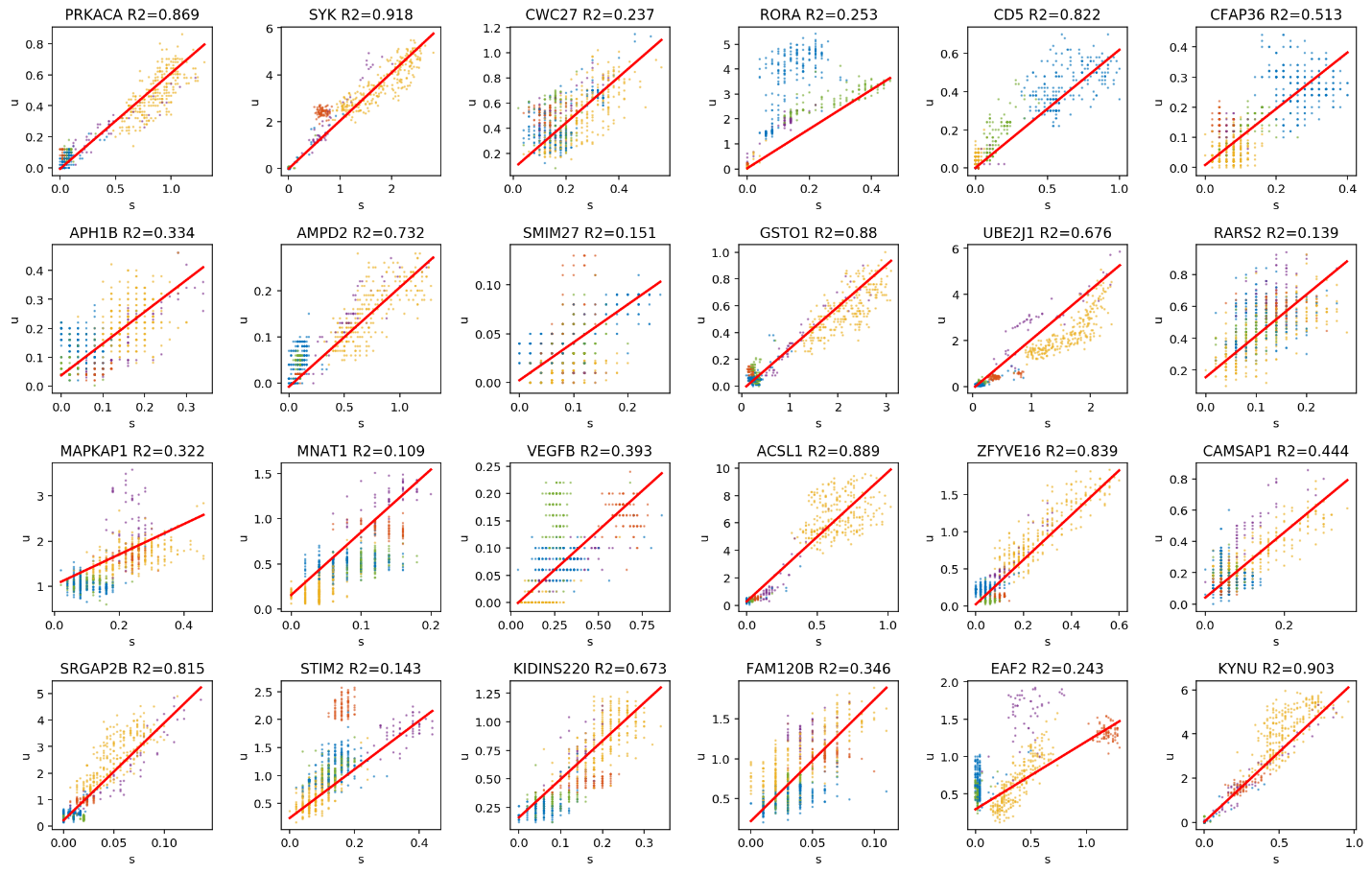


**Fig S11**. 10X 1k imputed spliced/unspliced RNA phase plots. 24 random phase plots out of 1114 genes used to calculate RNA velocity are shown. Linear $\gamma$ fit (red line) is plotted and fit $R^{2}$ is given in title. Color identifies cell type (blue: CD4+ T, red: B, yellow: monocytes, green: CD8+ T, purple: natural killer).


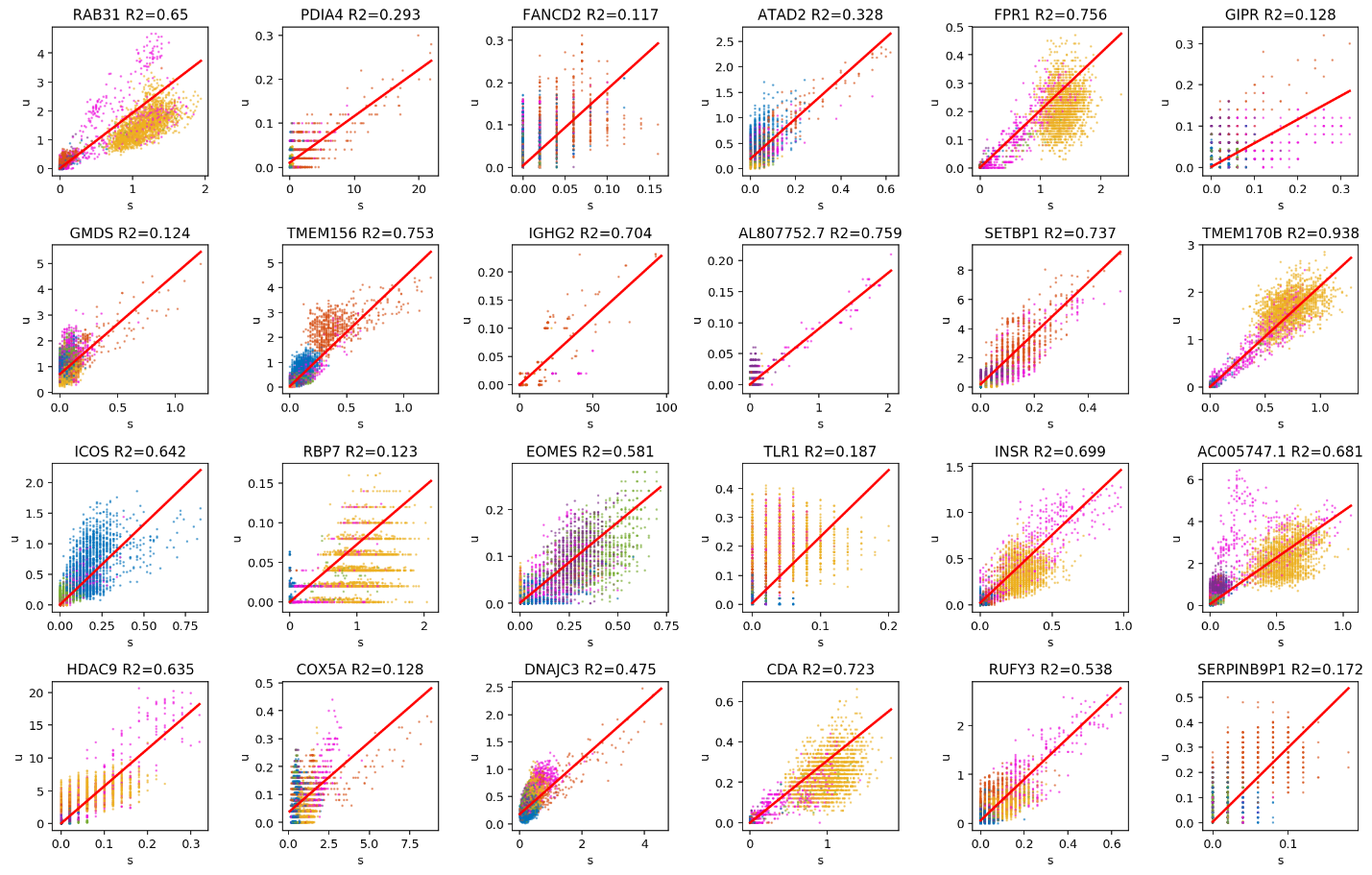


**Fig S12.** 10X 10k imputed spliced/unspliced RNA phase plots. 24 random phase plots out of 920 genes used to calculate RNA velocity are shown. Linear $\gamma$ fit (red line) is plotted and fit $R^{2}$ is given in title. Color identifies cell type (blue: CD4+ T, red: B, yellow: monocytes, green: CD8+ T, pink: not identifiable unambiguously).

## Cluster-specific RNA velocity


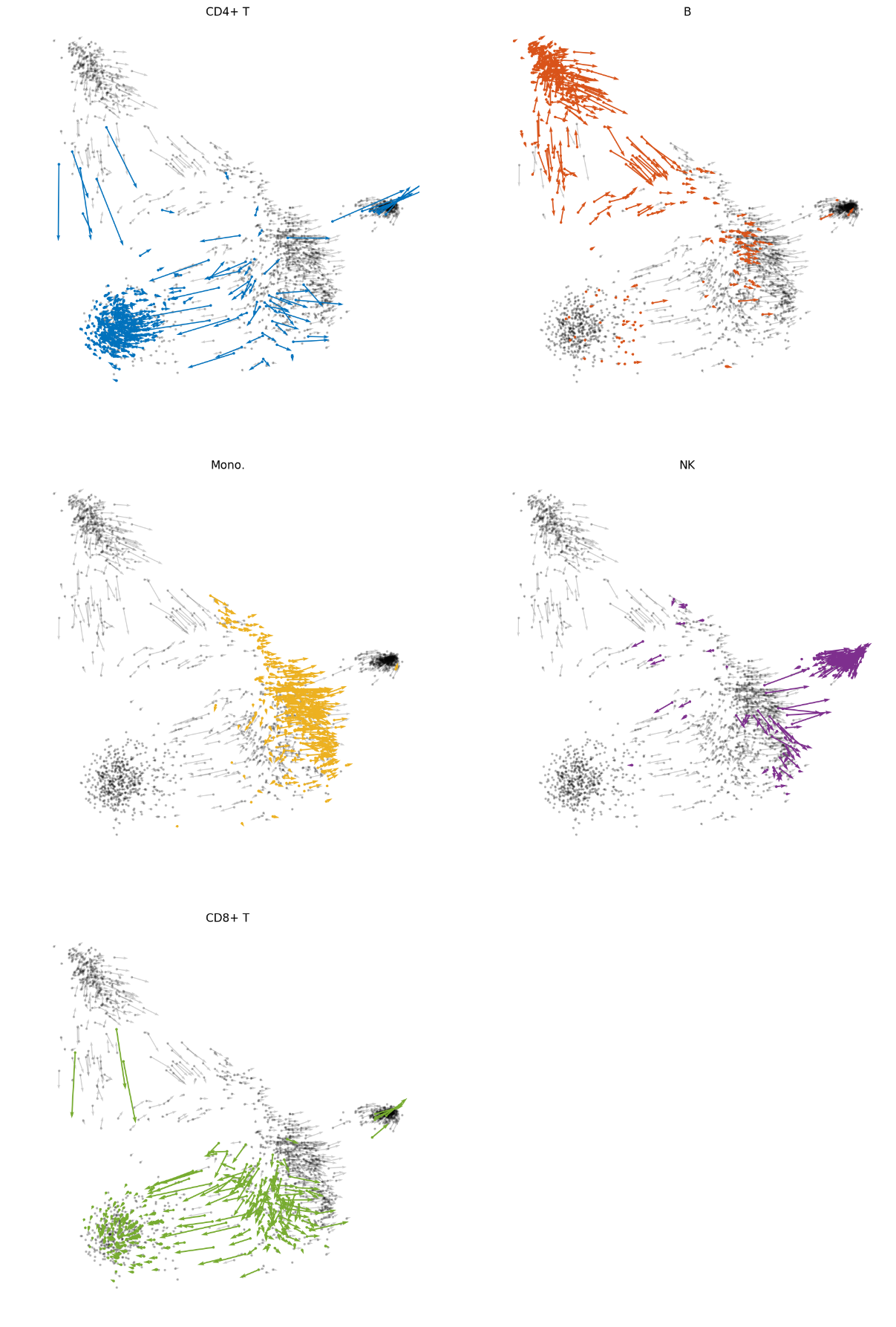


**Fig S13.** CITE-seq cell RNA velocities, distinguished by cell type. Color identifies cell type (blue: CD4+ T, red: B, yellow: monocytes, green: CD8+ T, purple: natural killer). Embedding: PC2/3.


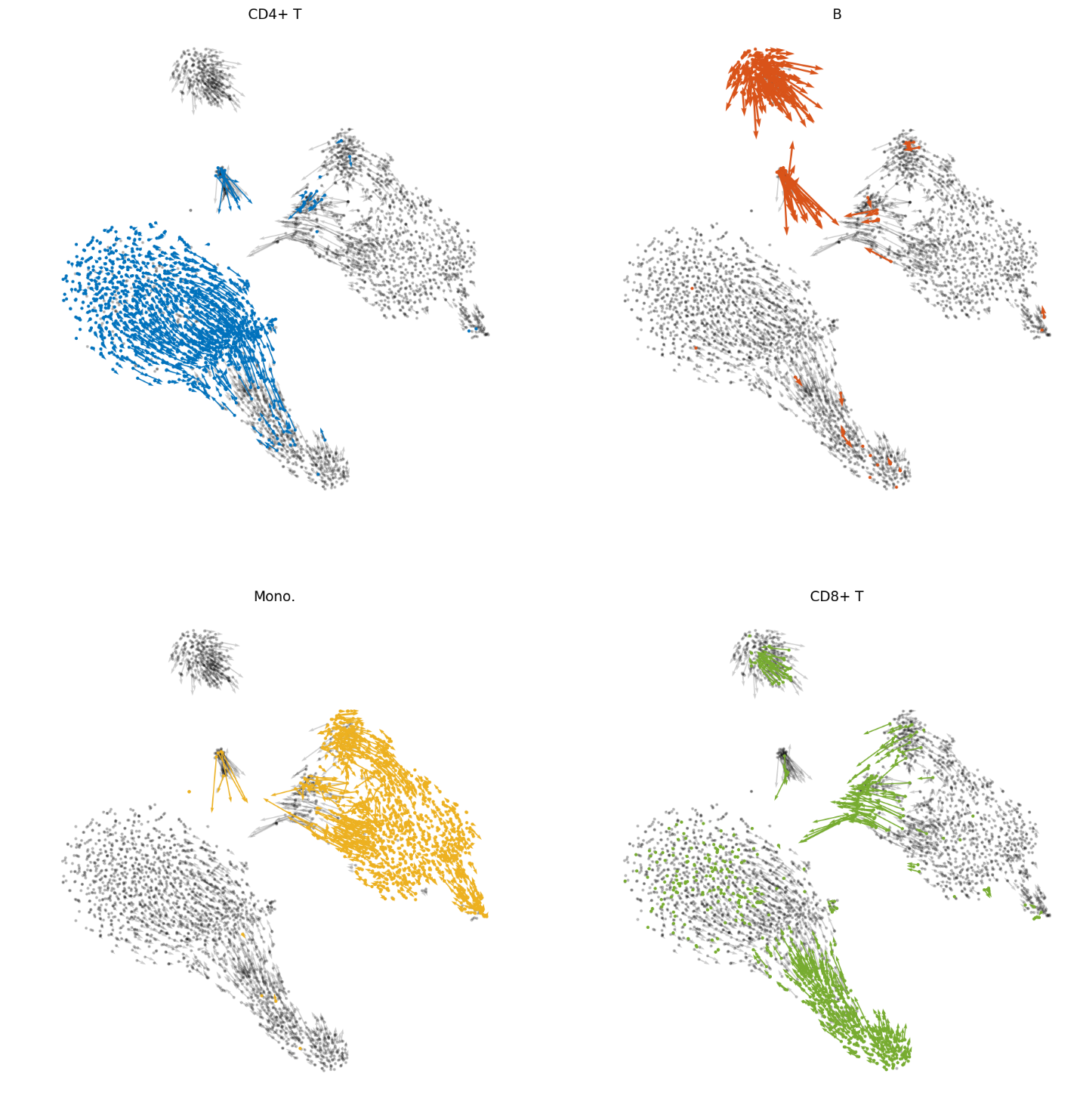


**Fig S14.** REAP-seq cell RNA velocities, distinguished by cell type. Color identifies cell type (blue: CD4+ T, red: B, yellow: monocytes, green: CD8+ T). Embedding: t-SNE.


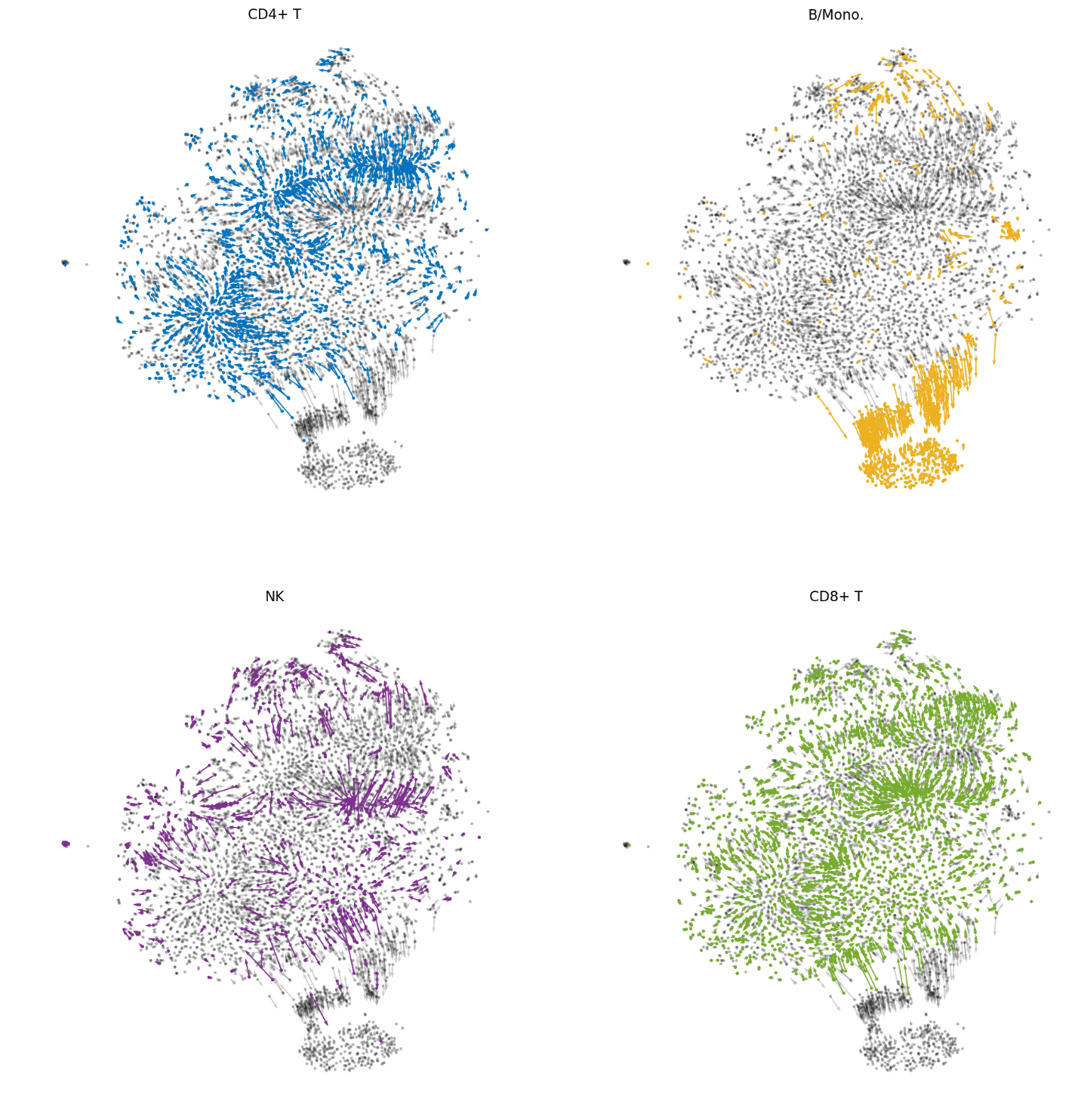


**Fig S15.** ECCITE-seq ctrl cell RNA velocities, distinguished by cell type. Color identifies cell type (blue: CD4+ T, yellow: monocytes, green: CD8+ T, purple: natural killer). Embedding: t-SNE.


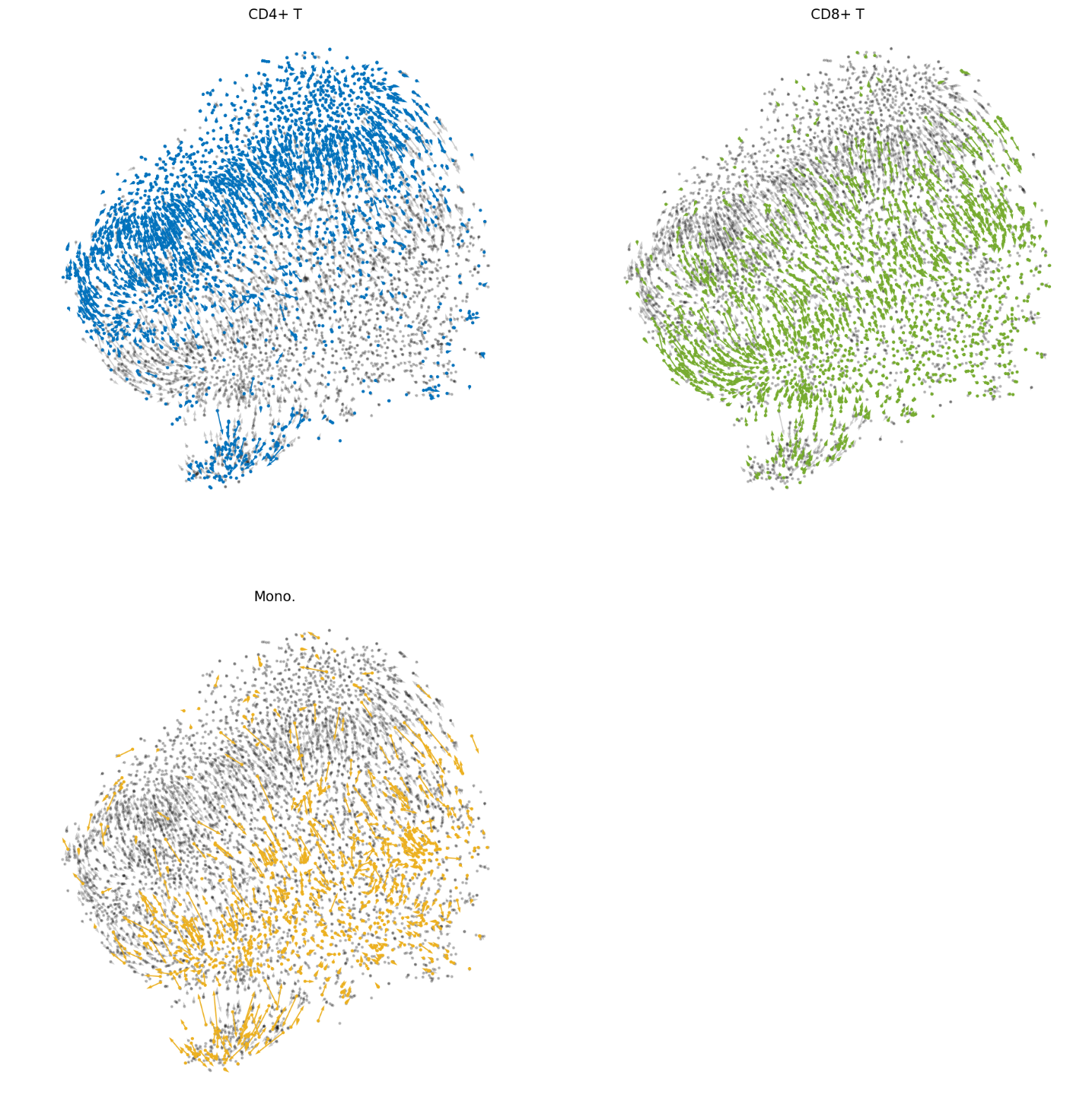


**Fig S16.** ECCITE-seq CTCL cell RNA velocities, distinguished by cell type. Color identifies cell type (blue: CD4+ T, yellow: monocytes, green: CD8+ T). Embedding: t-SNE.

**
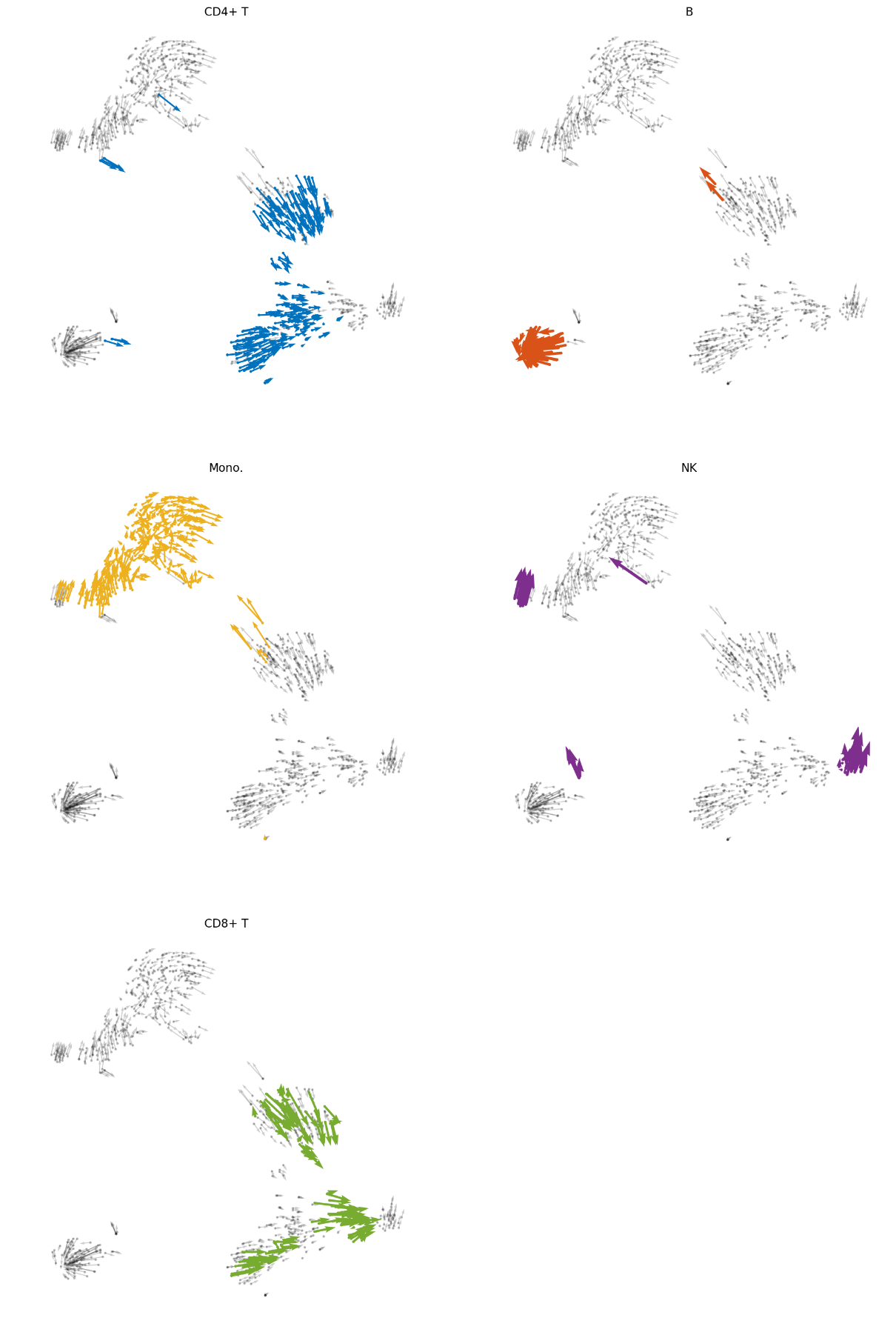
**

**Fig S17.** 10X 1k RNA velocities, distinguished by cell type. Color identifies cell type (blue: CD4+ T, red: B, yellow: monocytes, green: CD8+ T, purple: natural killer). Embedding: t-SNE.


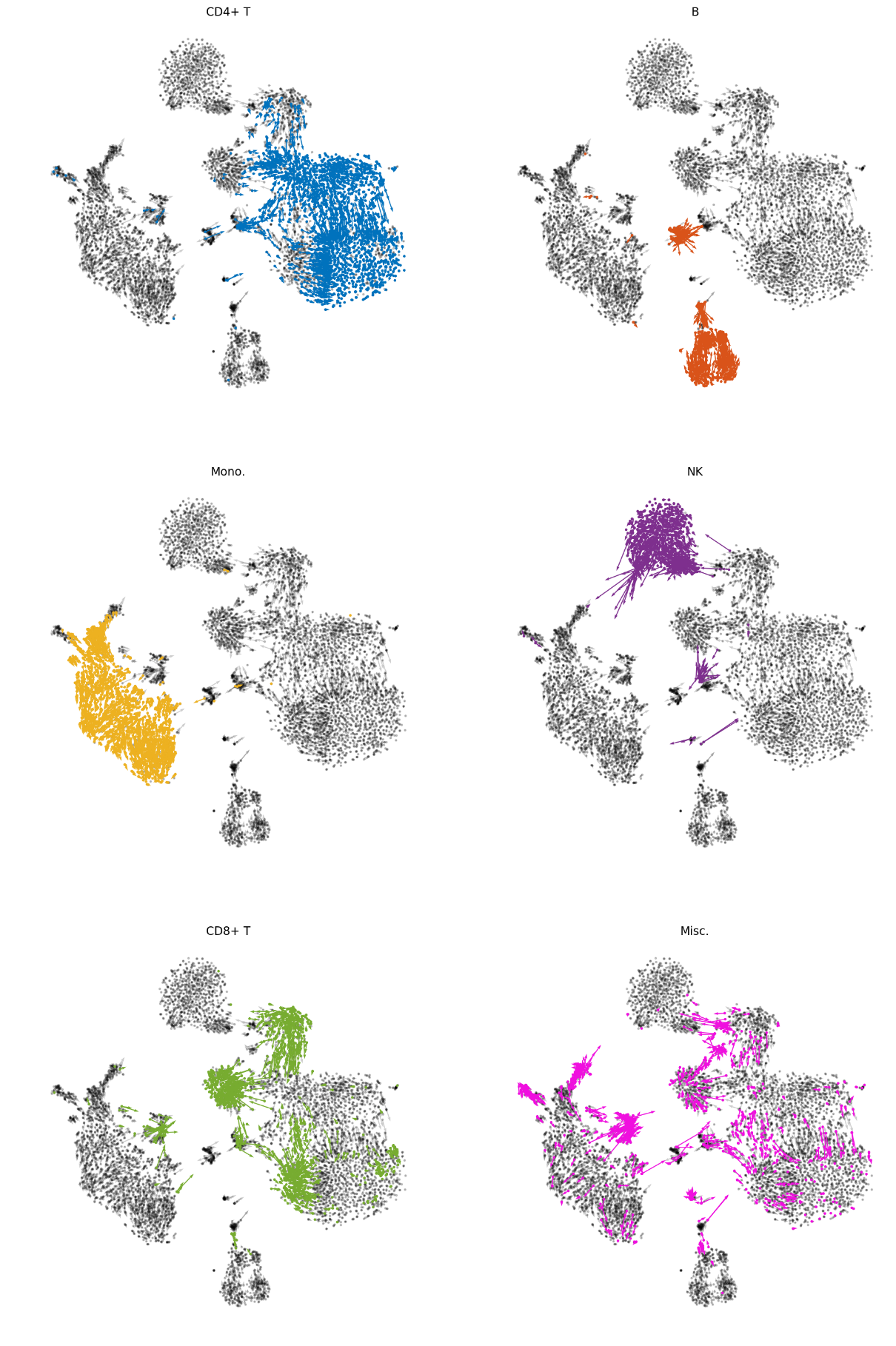


**Fig S18.** 10X 10k RNA velocities, distinguished by cell type. Color identifies cell type (blue: CD4+ T, red: B, yellow: monocytes, green: CD8+ T, pink: not identifiable unambiguously). Embedding: t-SNE.

## Cluster-specific protein velocity


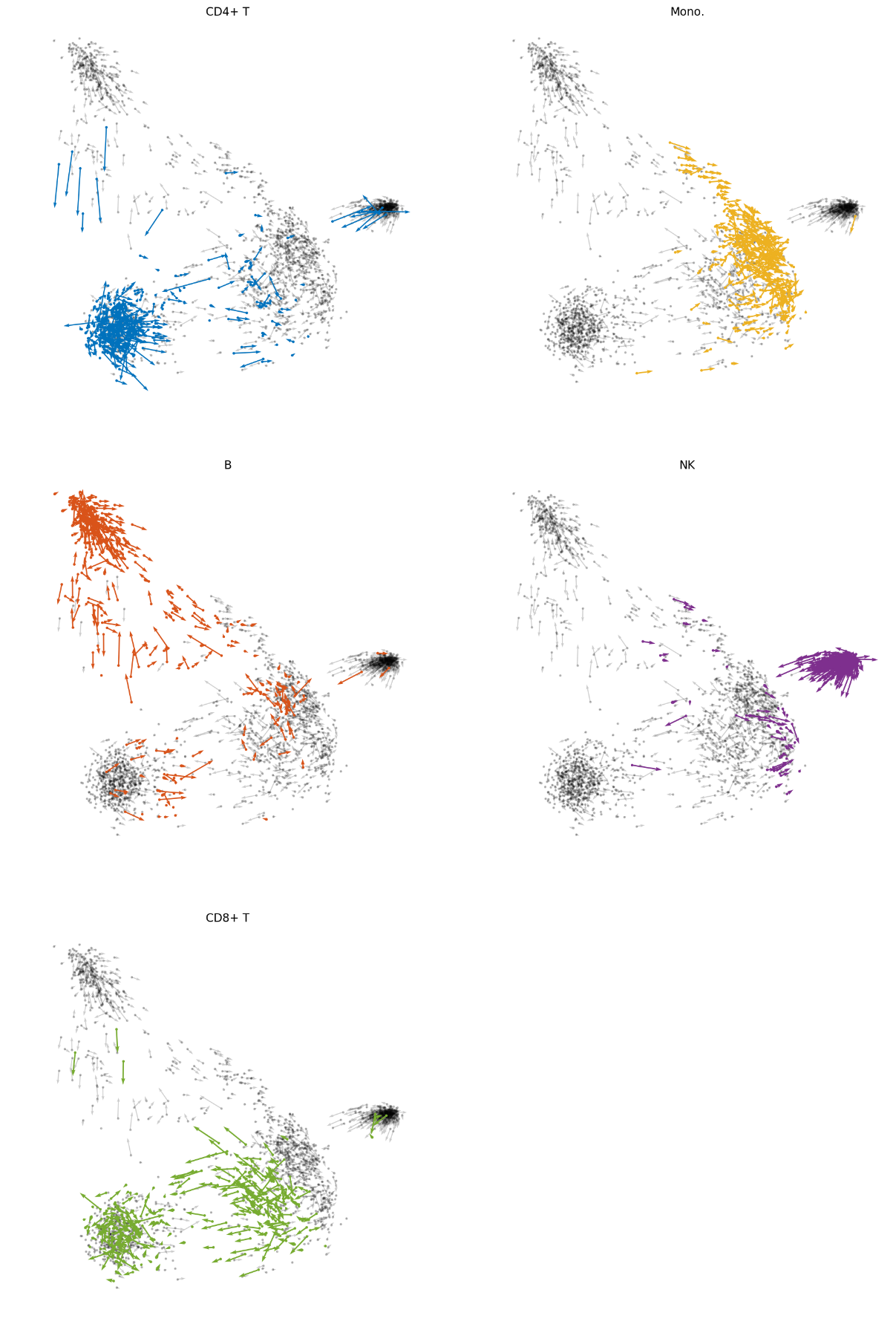


**Fig S19.** CITE-seq cell protein velocities, distinguished by cell type. Color identifies cell type (blue: CD4+ T, red: B, yellow: monocytes, green: CD8+ T, purple: natural killer). Embedding: PC2/3.


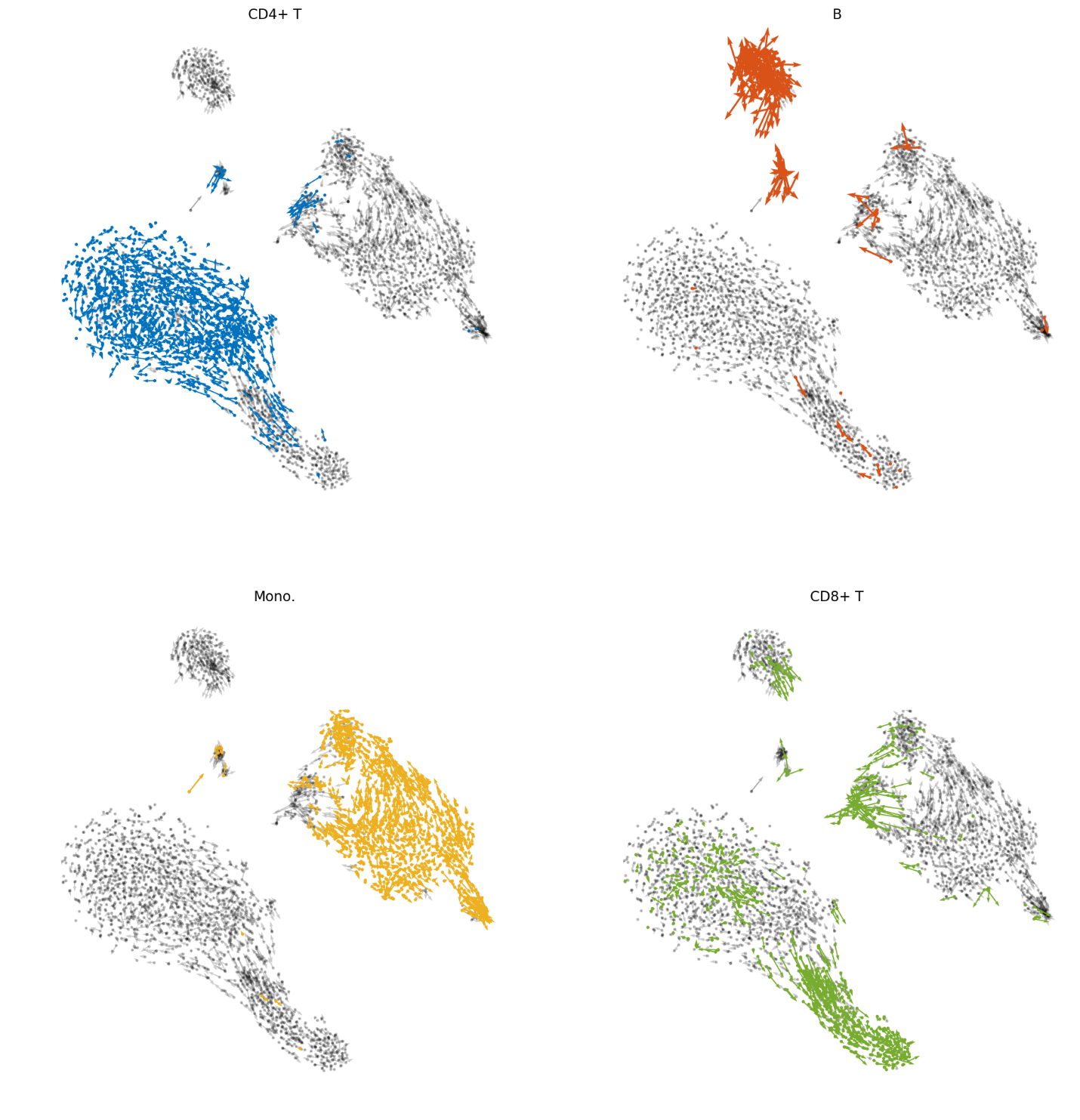


**Fig S20.** REAP-seq cell protein velocities, distinguished by cell type. Color identifies cell type (blue: CD4+ T, red: B, yellow: monocytes, green: CD8+ T). Embedding: t-SNE.


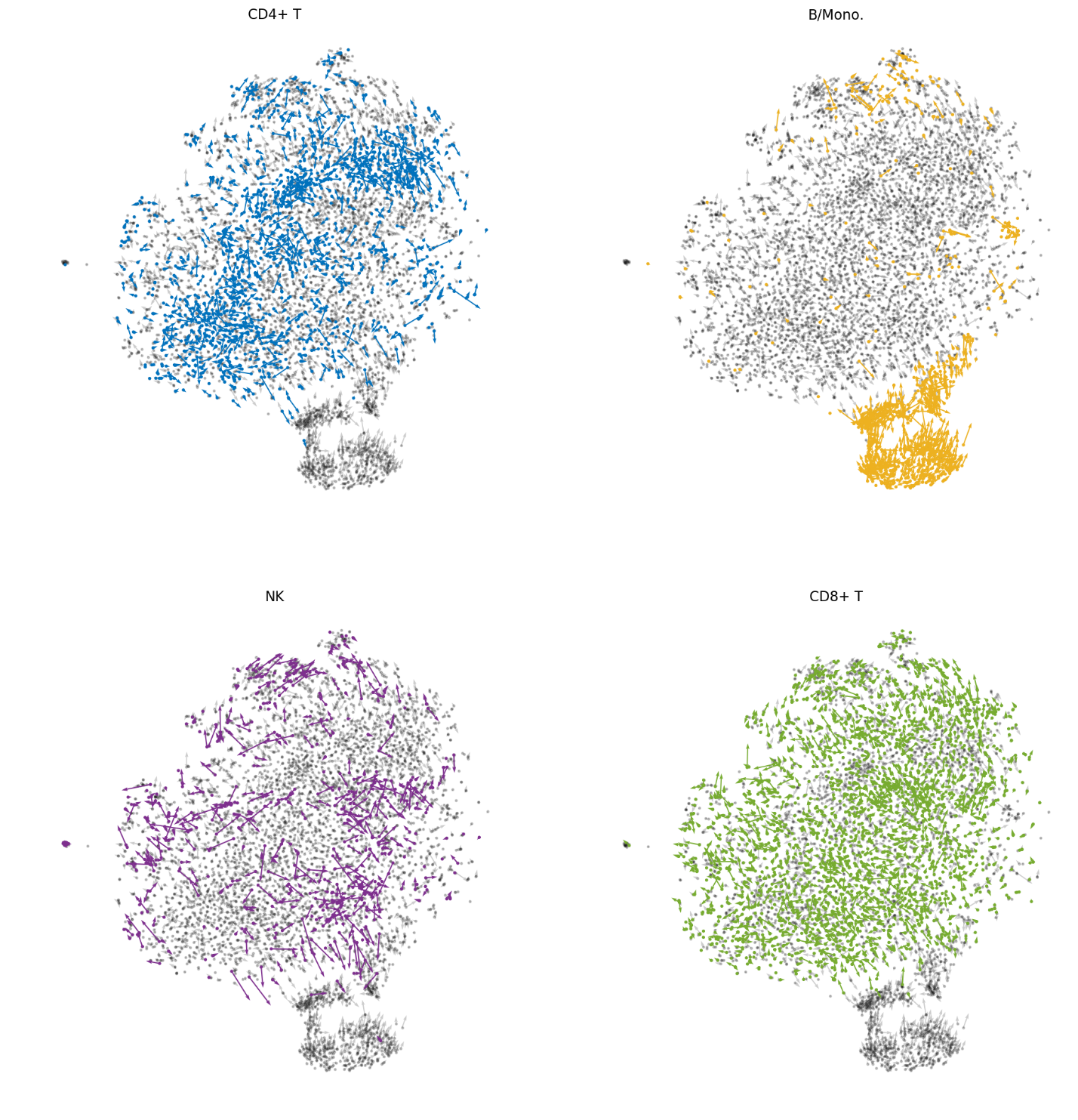


**Fig S21.** ECCITE-seq ctrl cell protein velocities, distinguished by cell type. Color identifies cell type (blue: CD4+ T, yellow: monocytes, green: CD8+ T, purple: natural killer). Embedding: t-SNE.


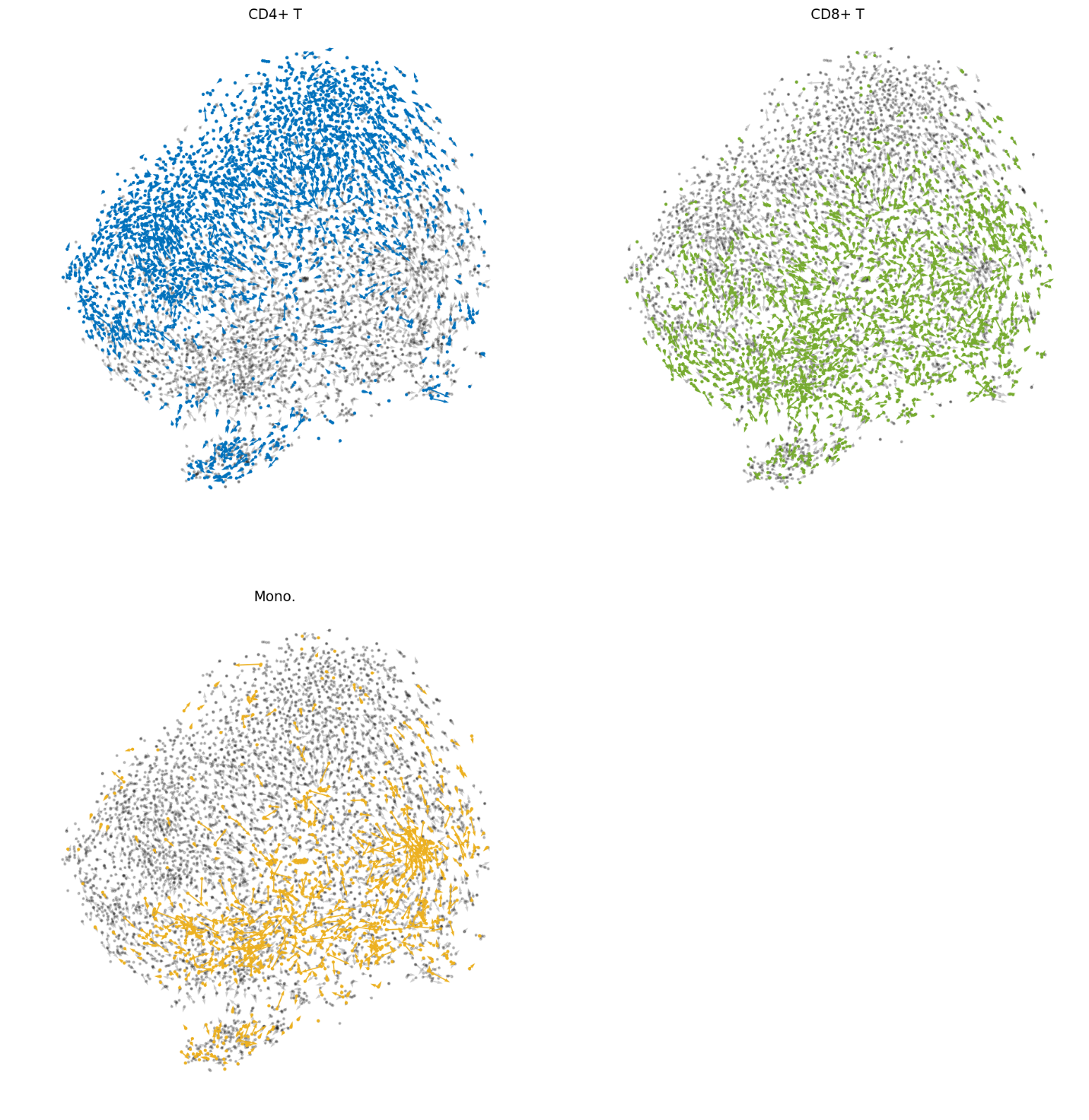


**Fig S22.** ECCITE-seq CTCL cell protein velocities, distinguished by cell type. Color identifies cell type (blue: CD4+ T, yellow: monocytes, green: CD8+ T). Embedding: t-SNE.


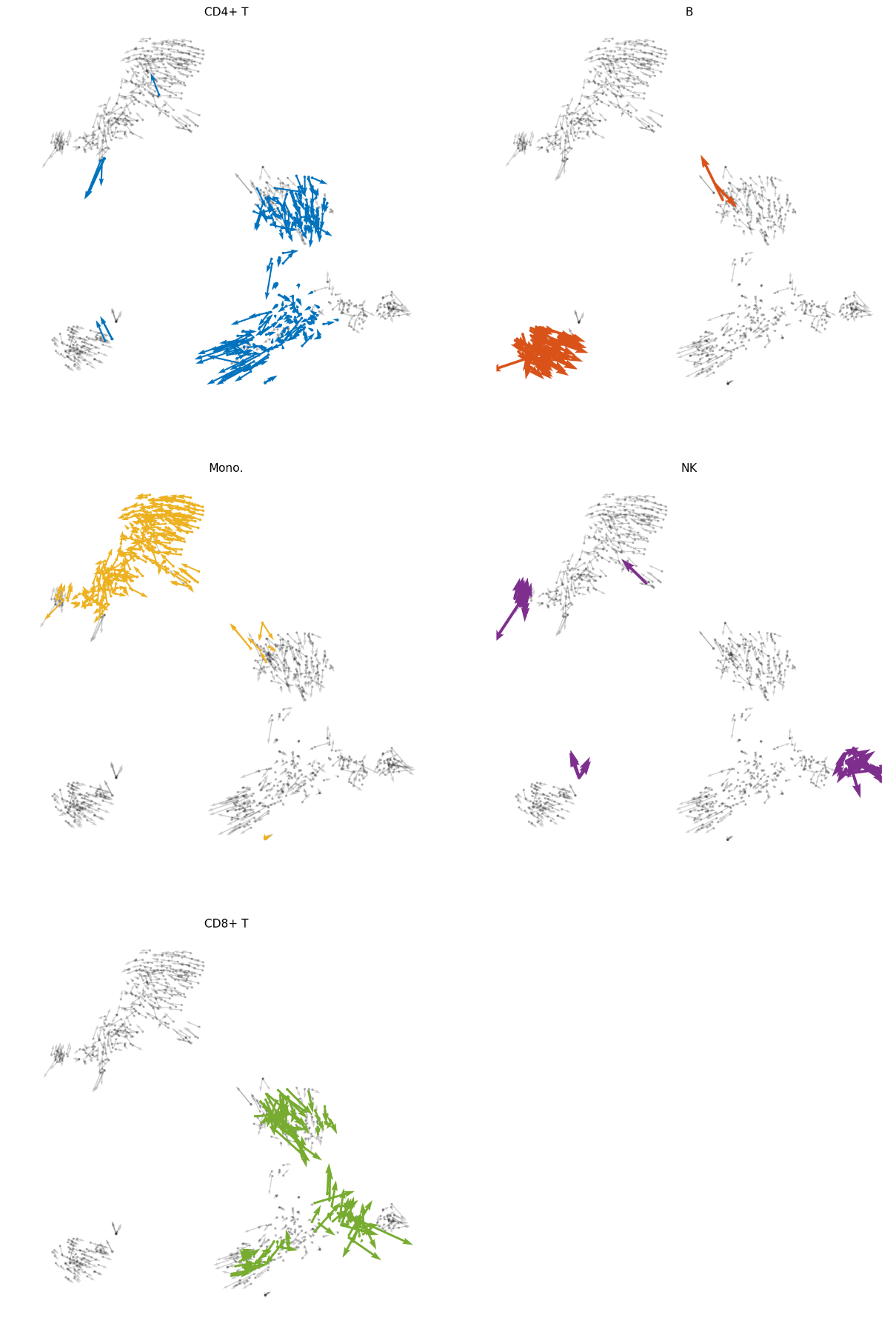


**Fig S23.** 10X 1k protein velocities, distinguished by cell type. Color identifies cell type (blue: CD4+ T, red: B, yellow: monocytes, green: CD8+ T). Embedding: t-SNE.


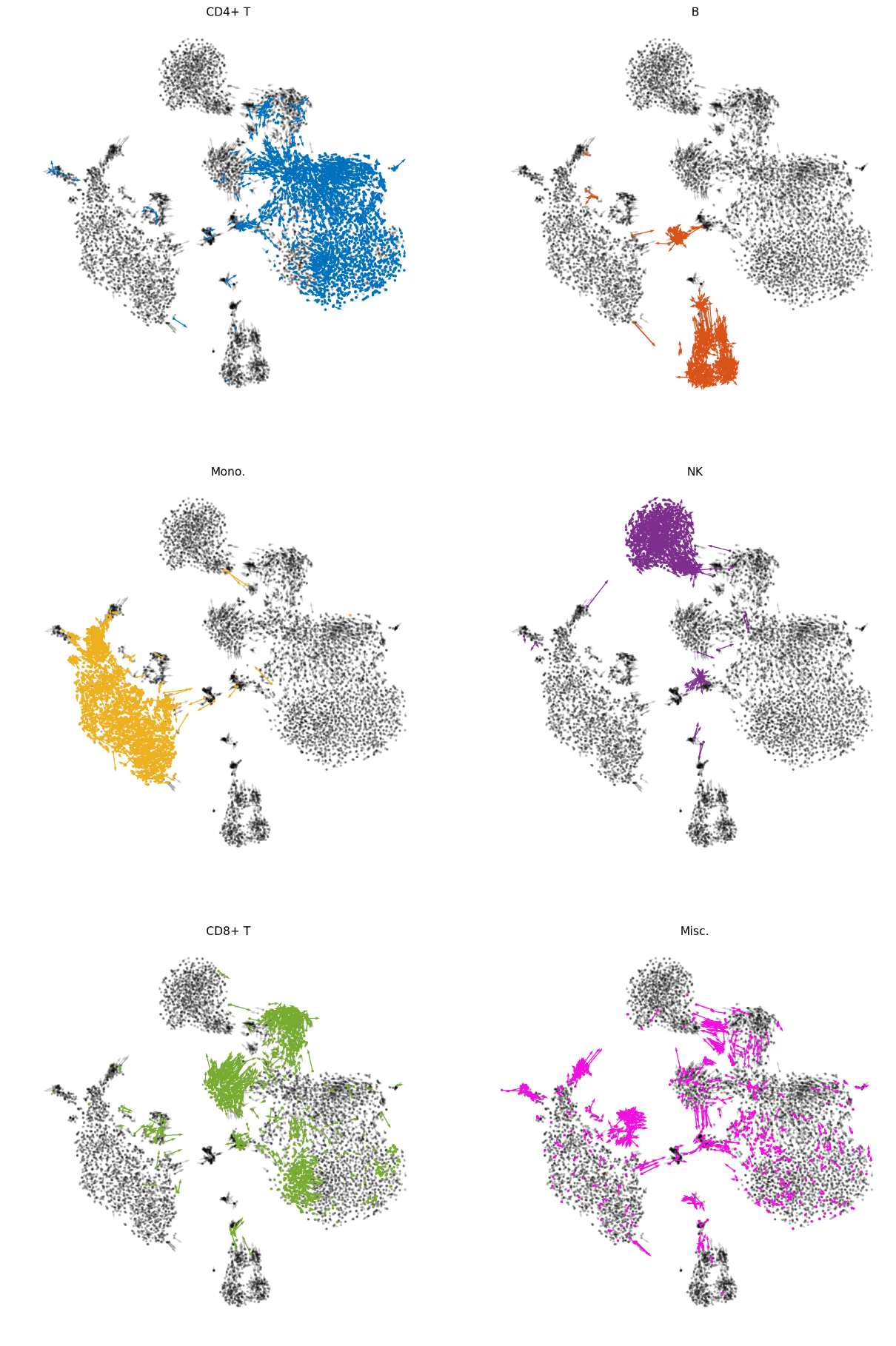


**Fig S24.** 10X 10k protein velocities, distinguished by cell type. Color identifies cell type (blue: CD4+ T, red: B, yellow: monocytes, green: CD8+ T, pink: not identifiable unambiguously). Embedding: t-SNE.

## Combined velocity visualizations


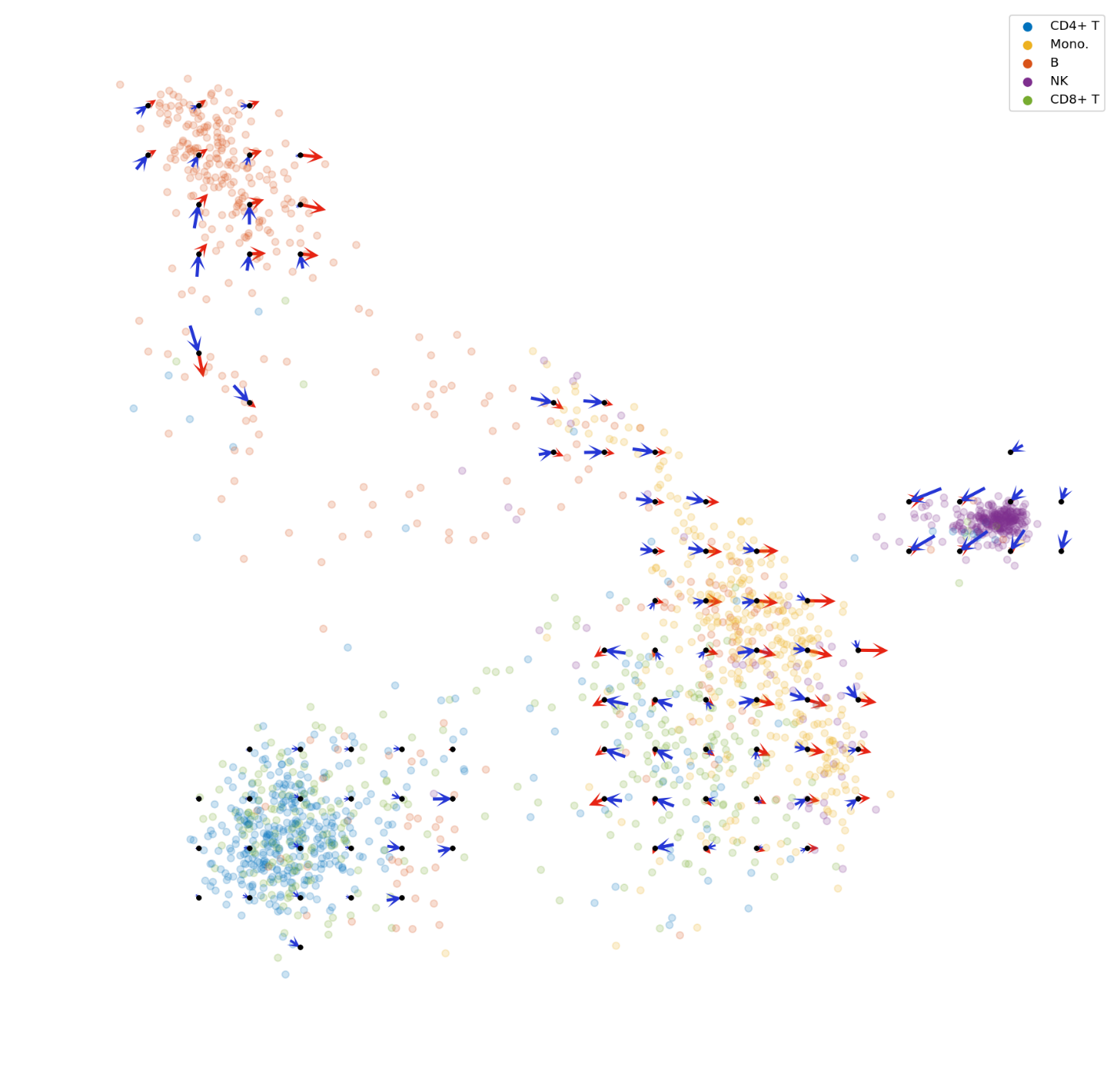


**Fig S25.** CITE-seq velocity fields visualized on a grid. Arrow color identifies velocity estimate (RNA: red, protein: blue). Dot color identifies cell type (blue: CD4+ T, red: B, yellow: monocytes, green: CD8+ T, purple: natural killer). Embedding: PC2/3.


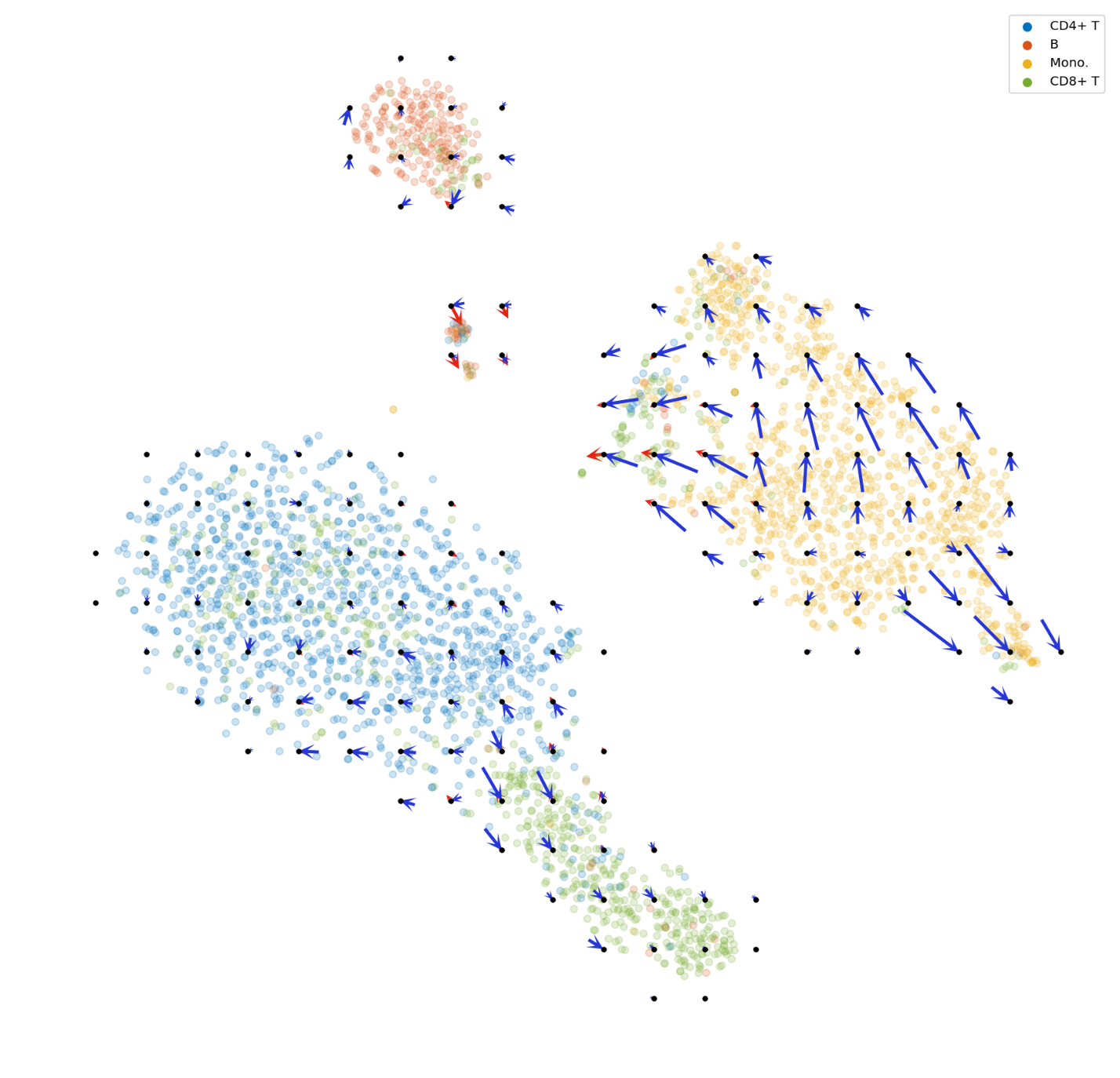


**Fig S26.** REAP-seq velocity fields visualized on a grid. Arrow color identifies velocity estimate (RNA: red, protein: blue). Dot color identifies cell type (blue: CD4+ T, red: B, yellow: monocytes, green: CD8+ T). Embedding: t-SNE.


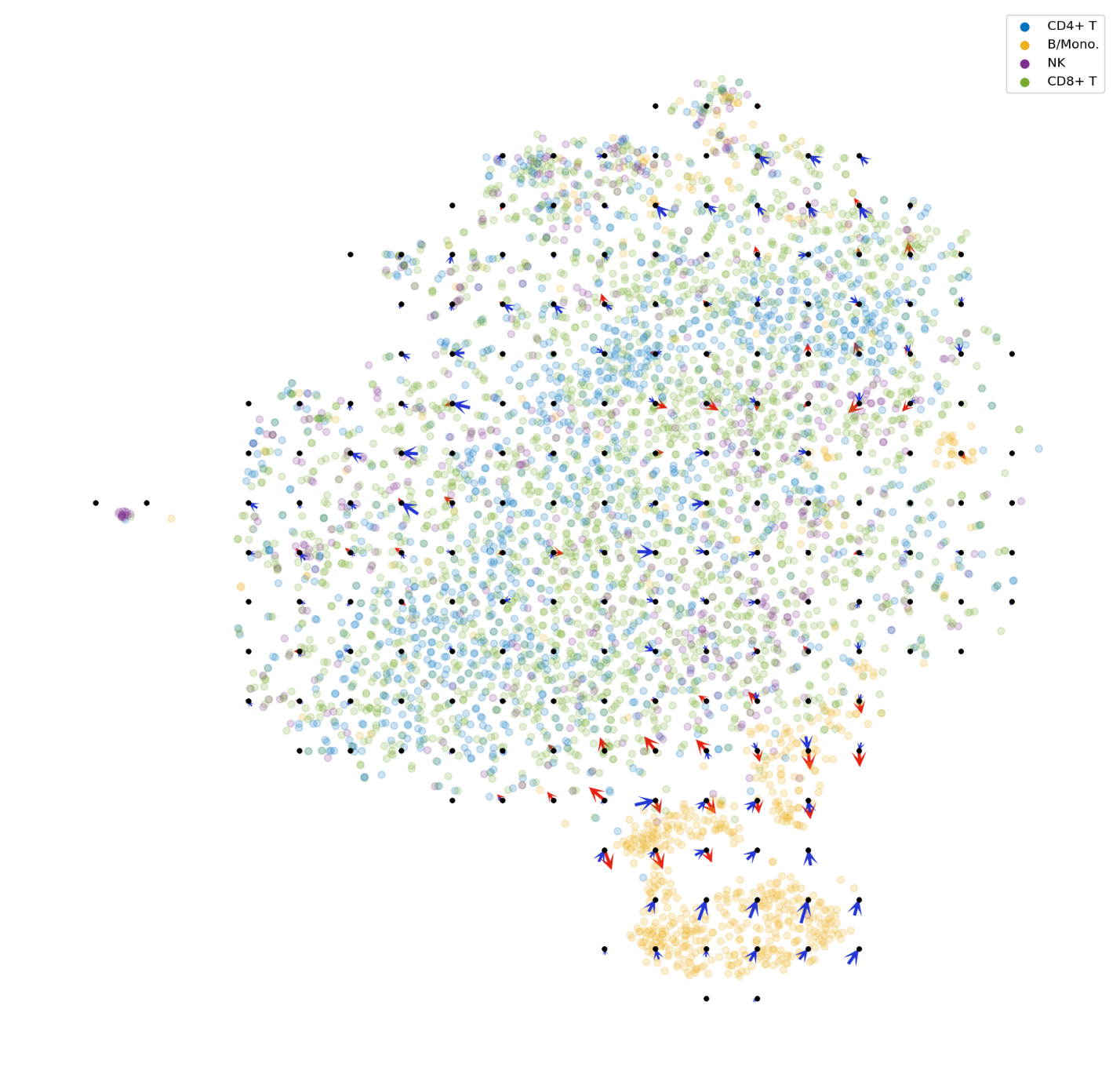


**Fig S27.** ECCITE-seq ctrl velocity fields visualized on a grid. Arrow color identifies velocity estimate (RNA: red, protein: blue). Dot color identifies cell type (blue: CD4+ T, yellow: monocytes, green: CD8+ T, purple: natural killer). Embedding: t-SNE.


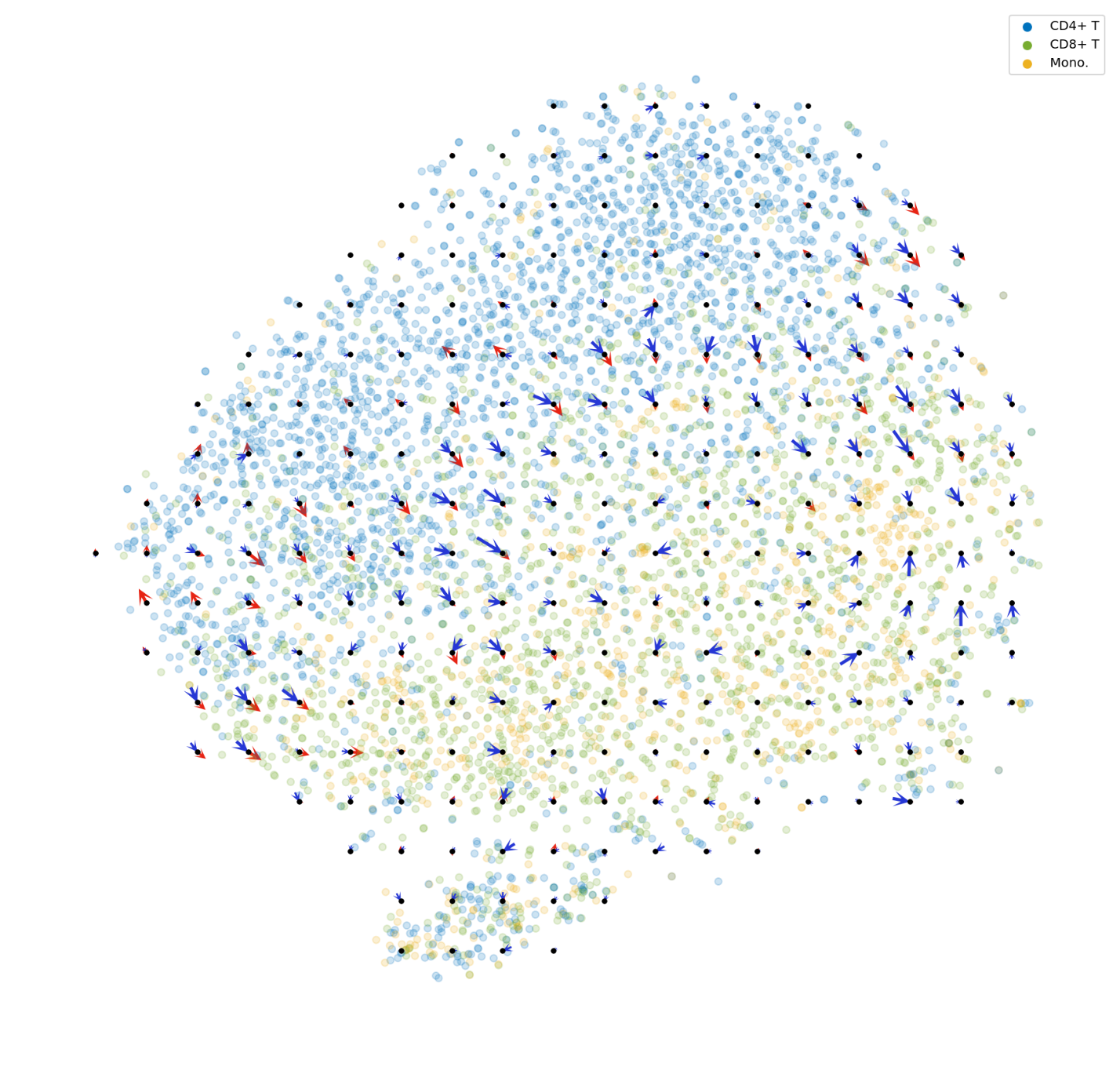


**Fig S28.** ECCITE-seq CTCL velocity fields visualized on a grid. Arrow color identifies velocity estimate (RNA: red, protein: blue). Dot color identifies cell type (blue: CD4+ T, yellow: monocytes, green: CD8+ T). Embedding: t-SNE.


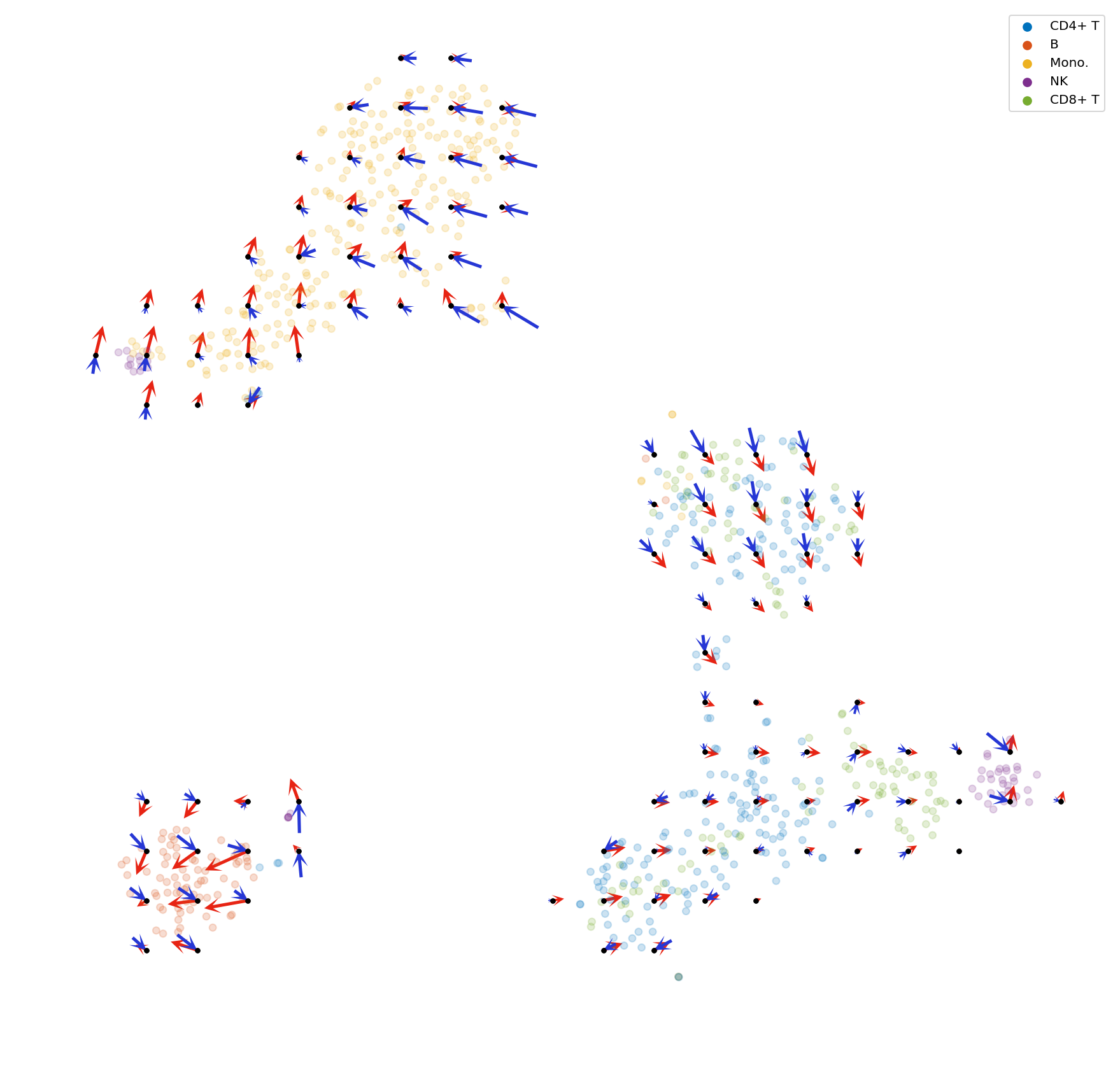


**Fig S29.** 10X 1k velocity fields visualized on a grid. Arrow color identifies velocity estimate (RNA: red, protein: blue). Dot color identifies cell type (blue: CD4+ T, red: B, yellow: monocytes, green: CD8+ T, purple: natural killer). Embedding: t-SNE.


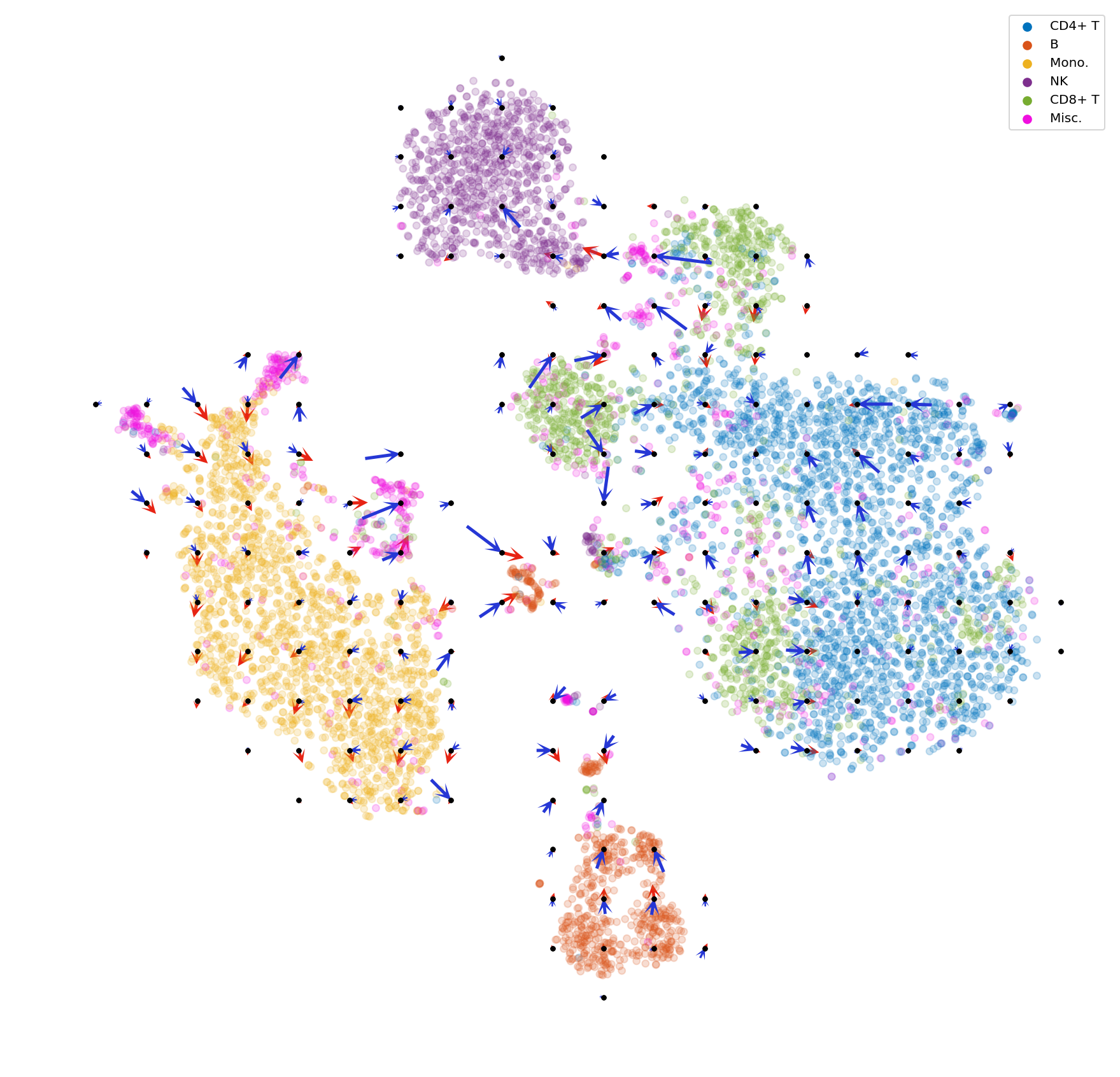


**Fig S30.** 10X 10k velocity fields visualized on a grid. Arrow color identifies velocity estimate (RNA: red, protein: blue). Dot color identifies cell type (blue: CD4+ T, red: B, yellow: monocytes, green: CD8+ T, purple: natural killer, pink: not identifiable unambiguously). Embedding: t-SNE.

## Acceleration landscapes


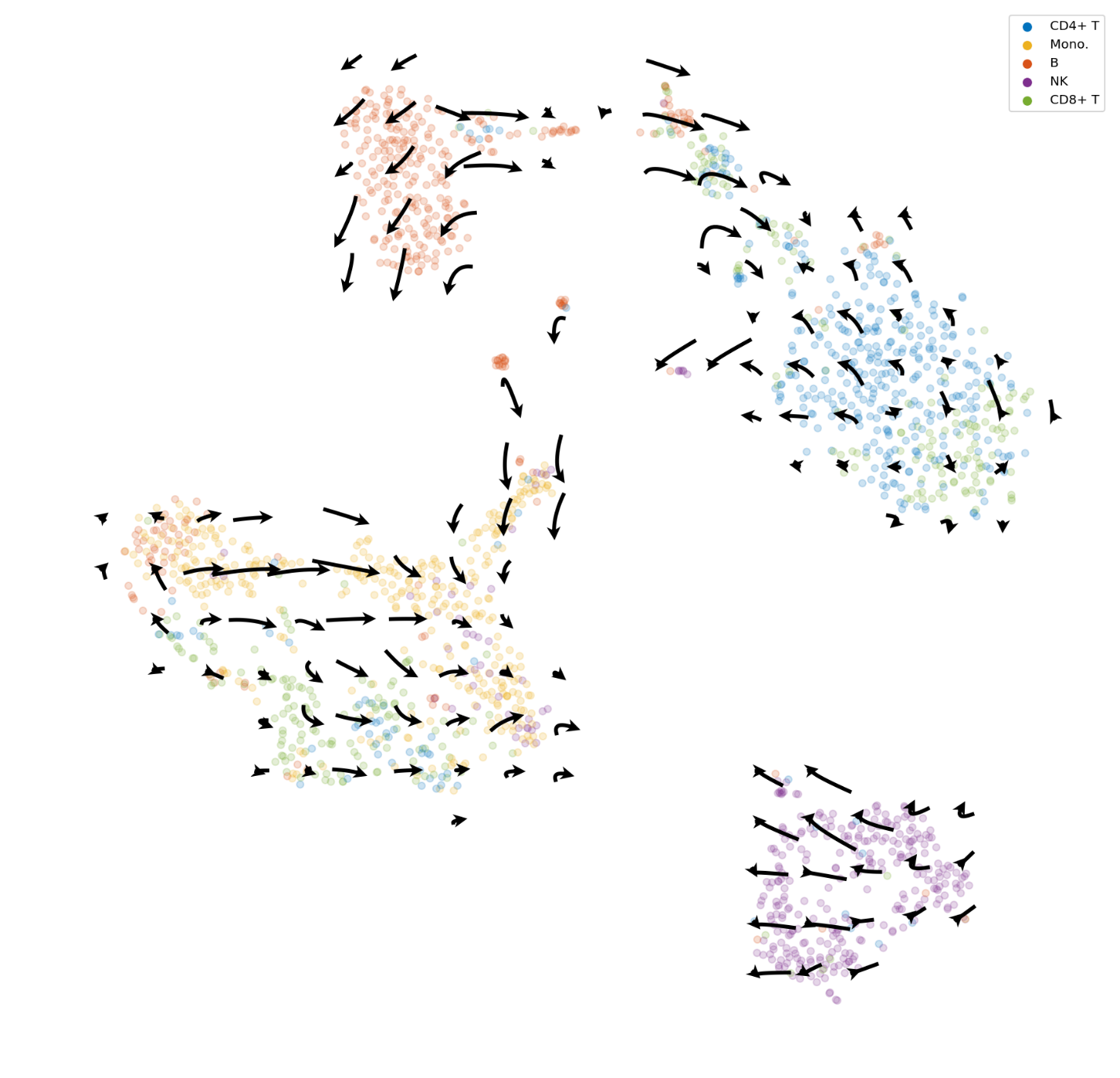


**Fig S31.** CITE-seq acceleration landscape. Dot color identifies cell type (blue: CD4+ T, red: B, yellow: monocytes, green: CD8+ T, purple: natural killer). Embedding: t-SNE.


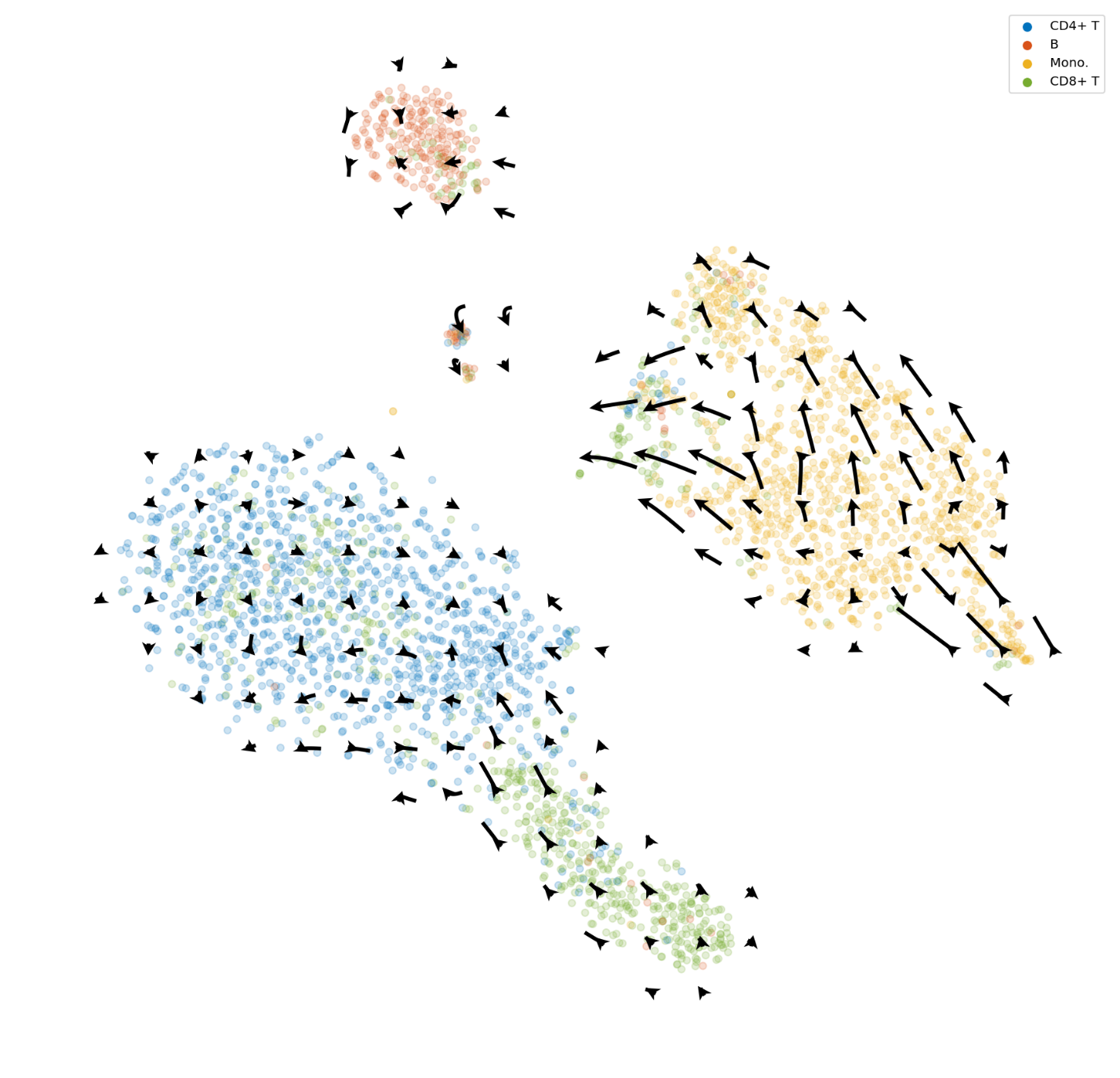


**Fig S32.** REAP-seq acceleration landscape. Dot color identifies cell type (blue: CD4+ T, red: B, yellow: monocytes, green: CD8+ T). Embedding: t-SNE.


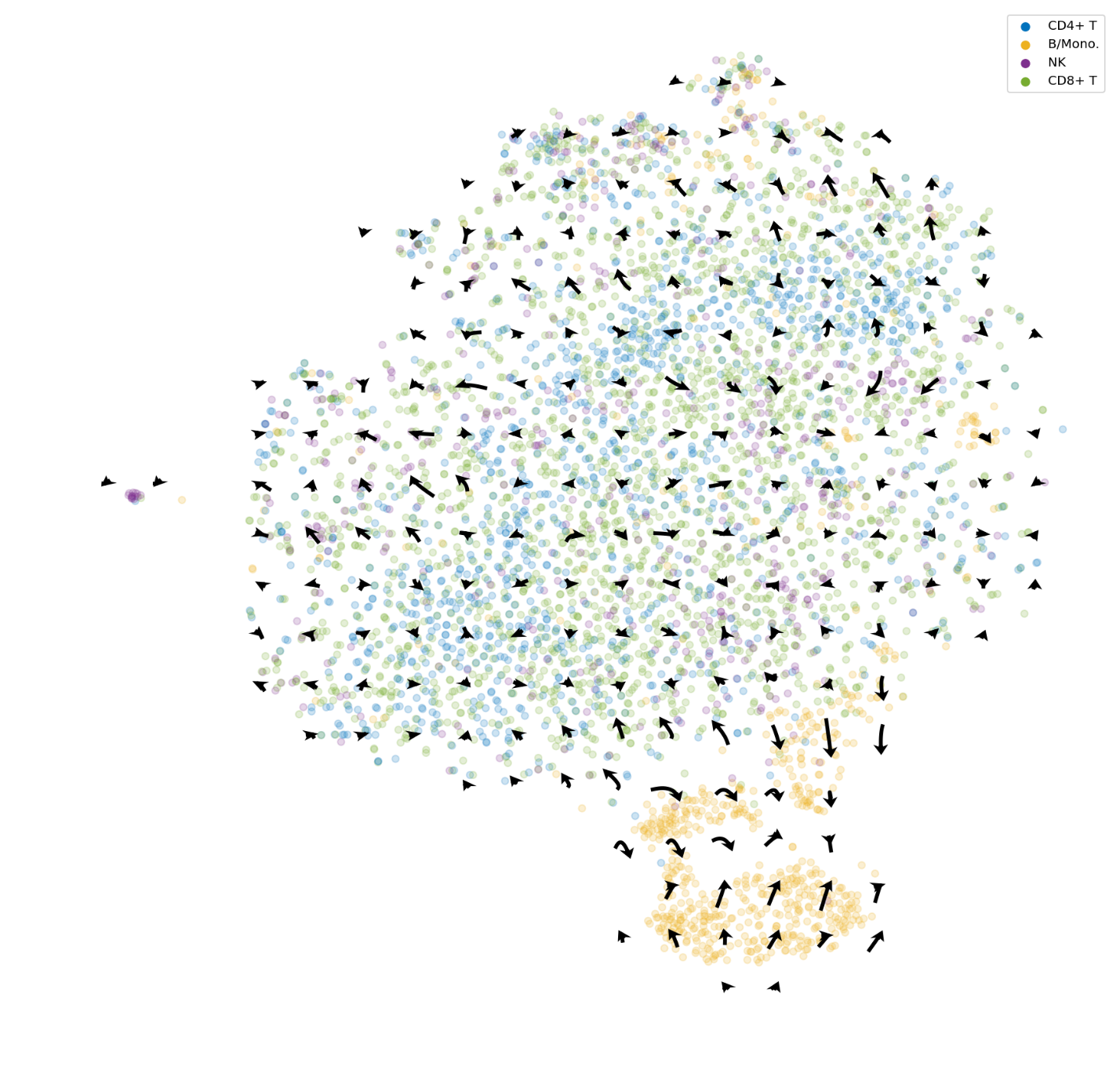


**Fig S33.** ECCITE-seq ctrl acceleration landscape. Dot color identifies cell type (blue: CD4+ T, yellow: monocytes, green: CD8+ T, purple: natural killer).


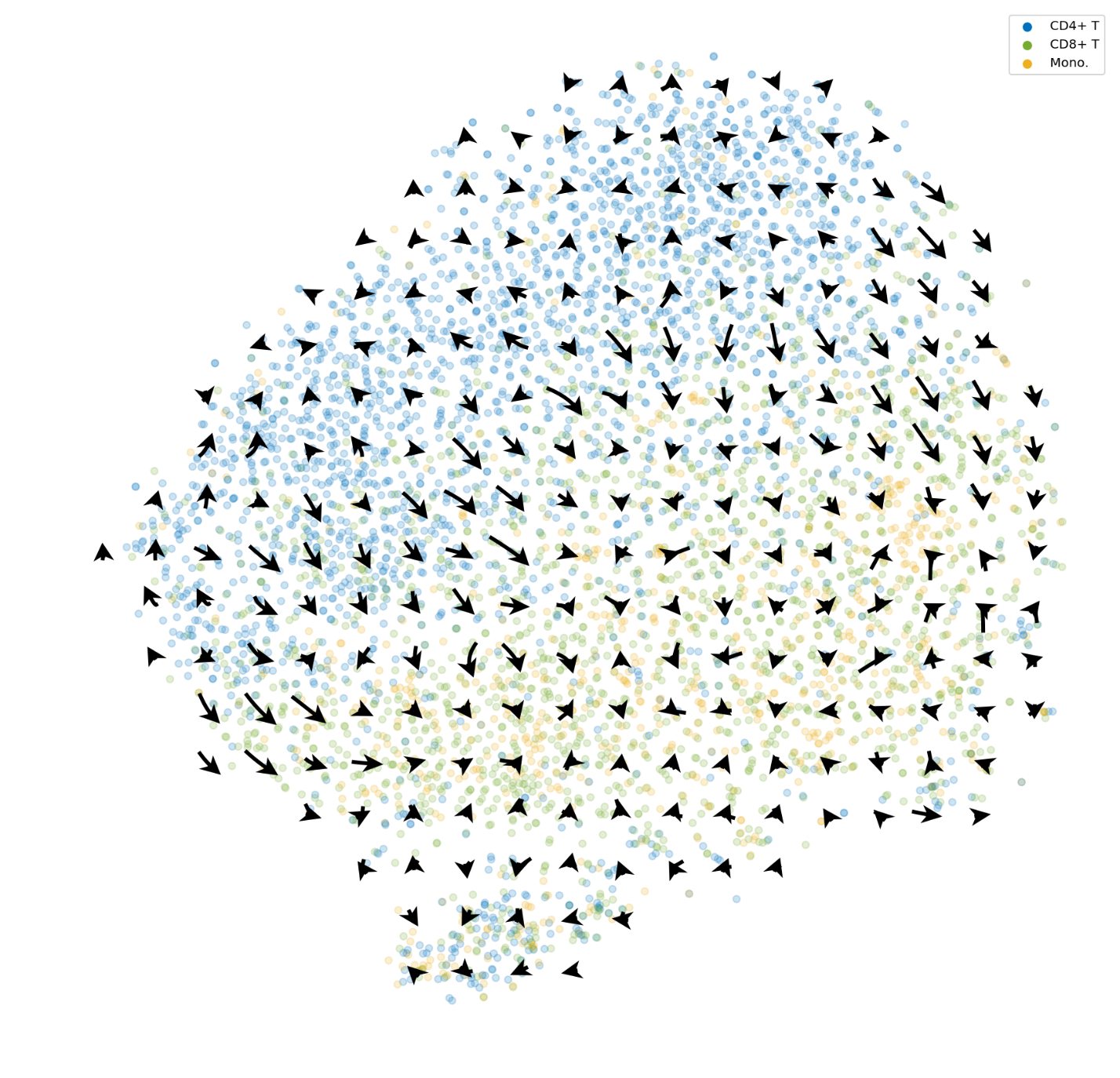


**Fig S34.** ECCITE-seq CTCL acceleration landscape. Dot color identifies cell type (blue: CD4+ T, yellow: monocytes, green: CD8+ T).


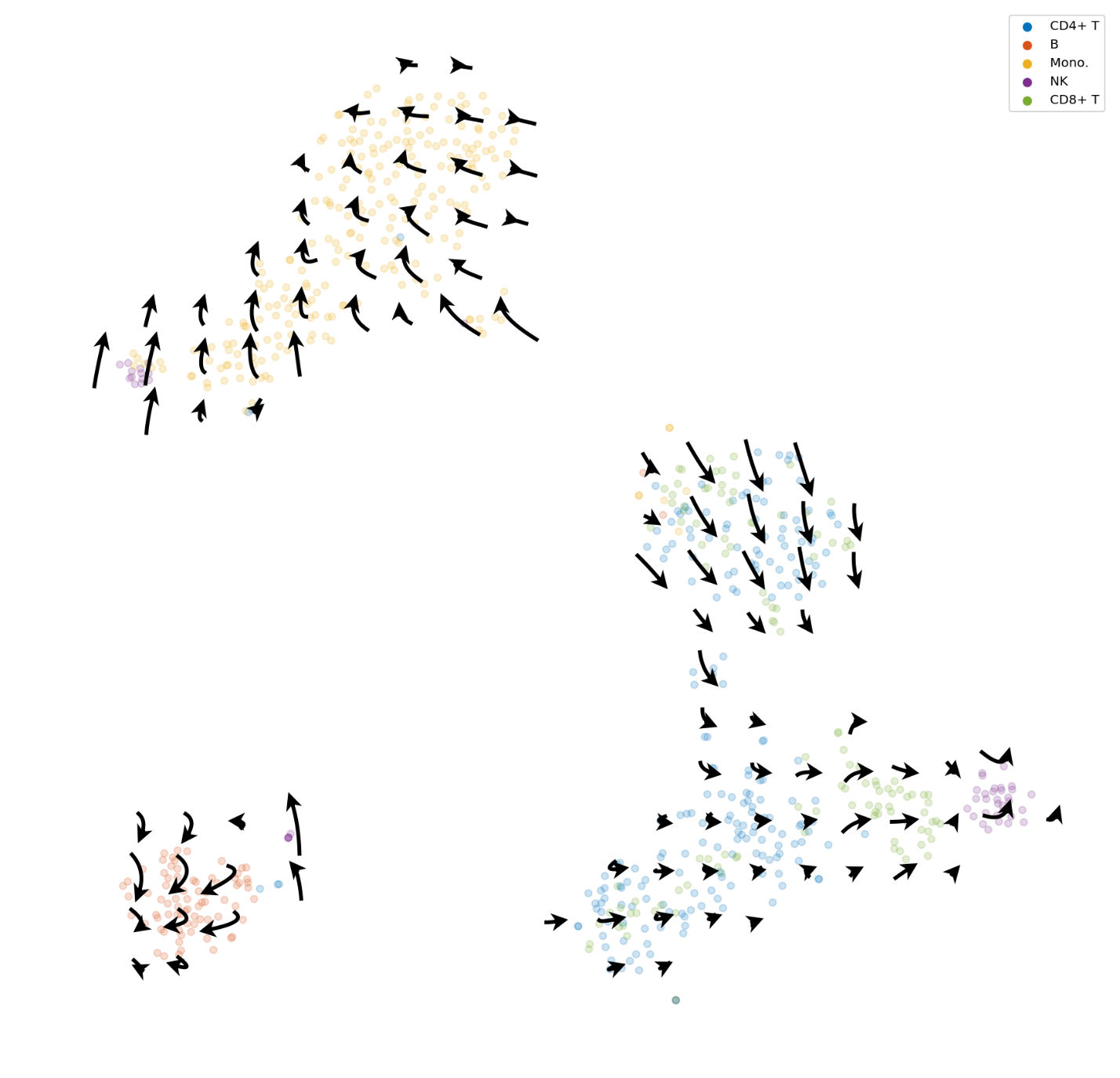


**Fig S35.** 10X 1k acceleration landscape. Dot color identifies cell type (blue: CD4+ T, red: B, yellow: monocytes, green: CD8+ T, purple: natural killer). Embedding: t-SNE.


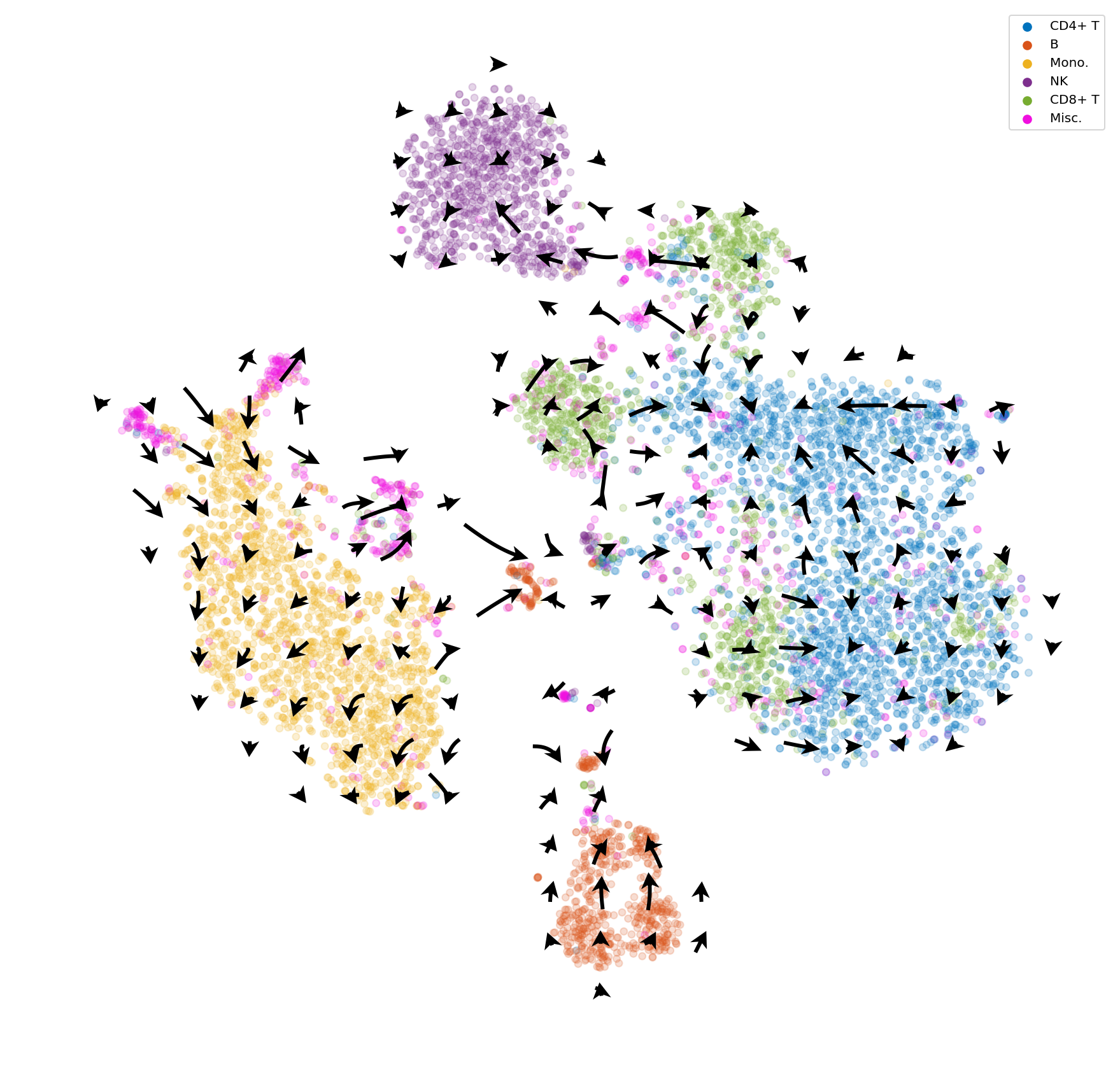


**Fig S36**. 10X 10k acceleration landscape. Dot color identifies cell type (blue: CD4+ T, red: B, yellow: monocytes, green: CD8+ T, purple: natural killer, pink: not identifiable unambiguously). Embedding: t-SNE.

## Sequencing quality comparison


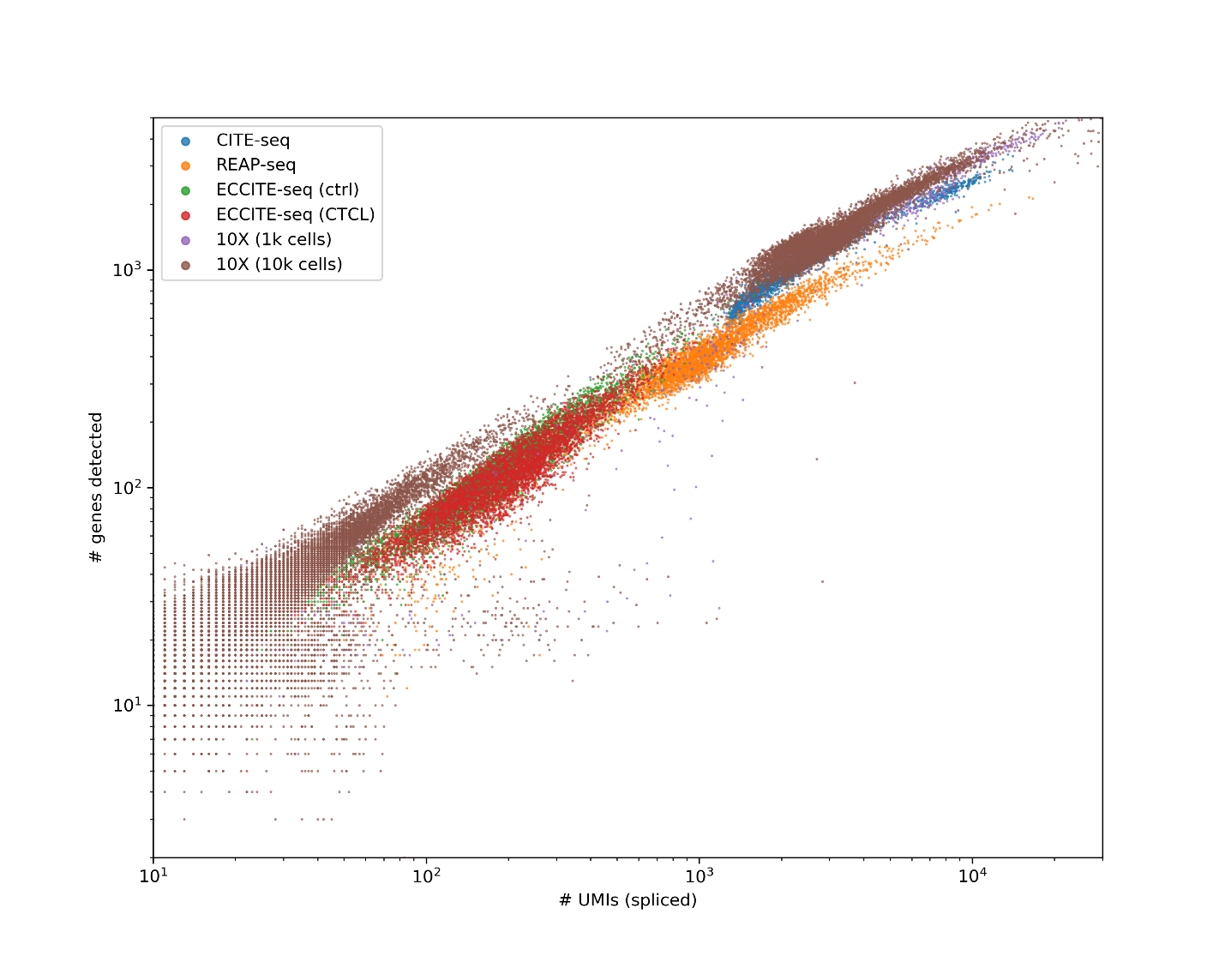


**Fig S37.** Depth comparison between analyzed datasets. Each dot represents one cell in the dataset. The cell’s number of spliced RNA molecules identified by the pre-processing pipeline is shown on the abscissa; the number of genes with non-zero spliced RNA counts is shown on the ordinate. The 10X data is pseudoaligned and unfiltered; the low-UMI population corresponds to empty droplets.

## Cell type identification


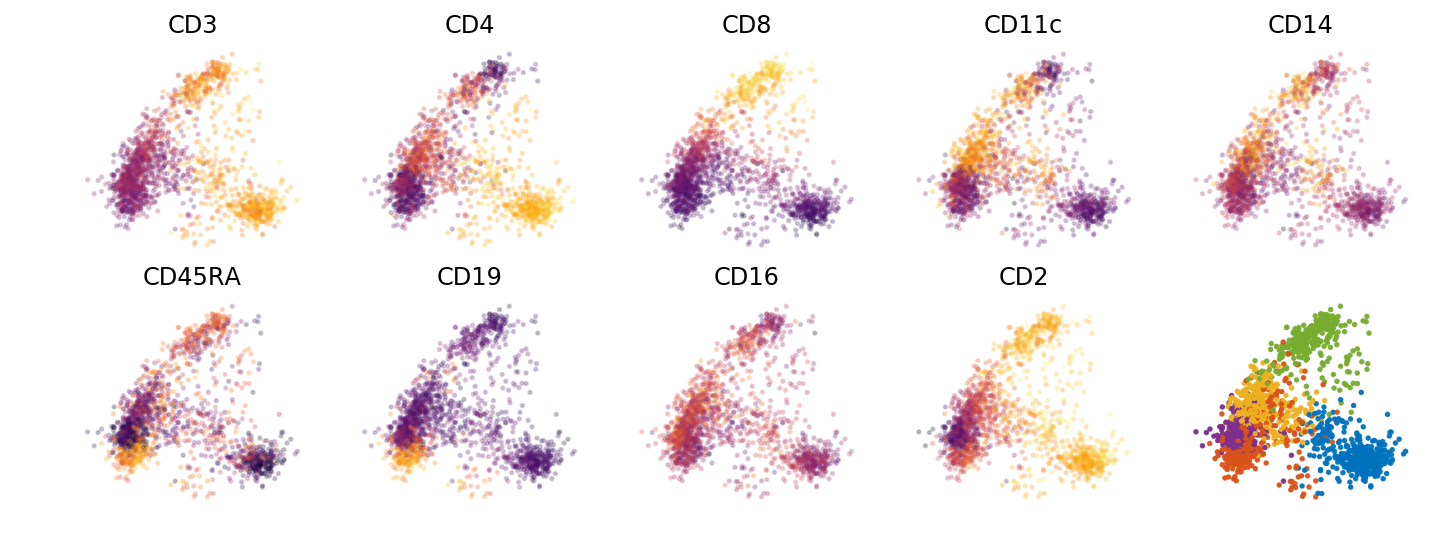


**Fig S38.** CITE-seq cell surface marker abundance and cluster definitions. Marker-specific plot dot color identifies marker abundance (yellow is high, black is low). Cluster plot dot color identifies cell type (blue: CD4+ T, red: B, yellow: monocytes, green: CD8+ T, purple: natural killer). Embedding: Protein PC2/3.


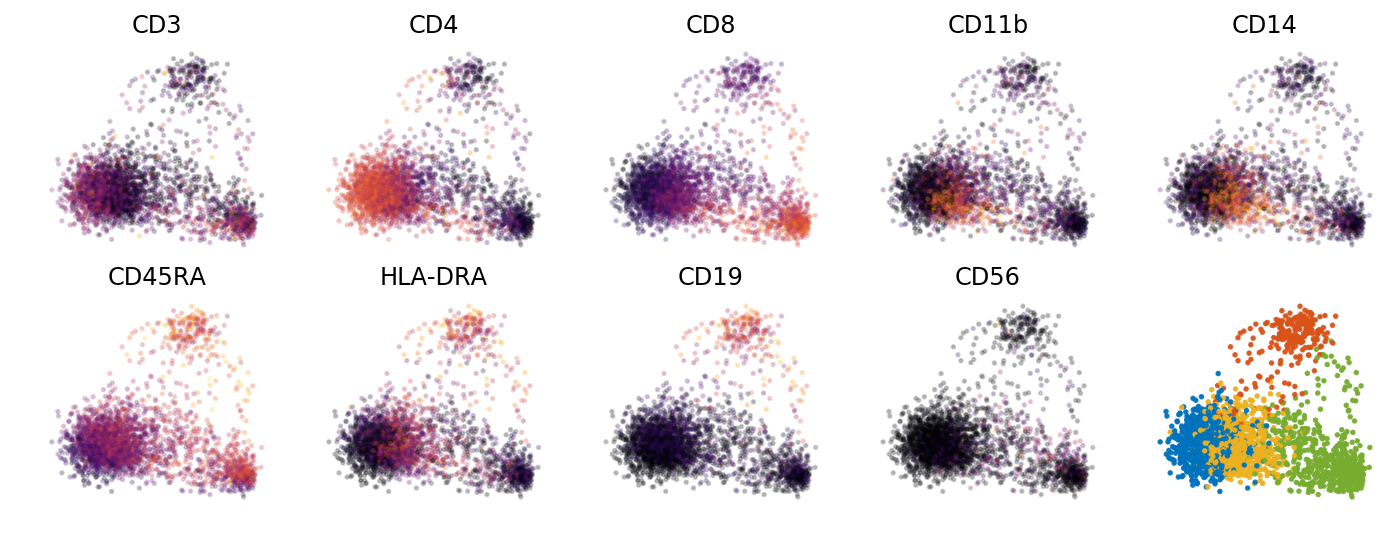


**Fig S39.** REAP-seq cell surface marker abundance and cluster definitions. Marker-specific plot dot color identifies marker abundance (yellow is high, black is low). Cluster plot dot color identifies cell type (blue: CD4+ T, red: B, yellow: monocytes, green: CD8+ T). Embedding: Protein PC2/3.


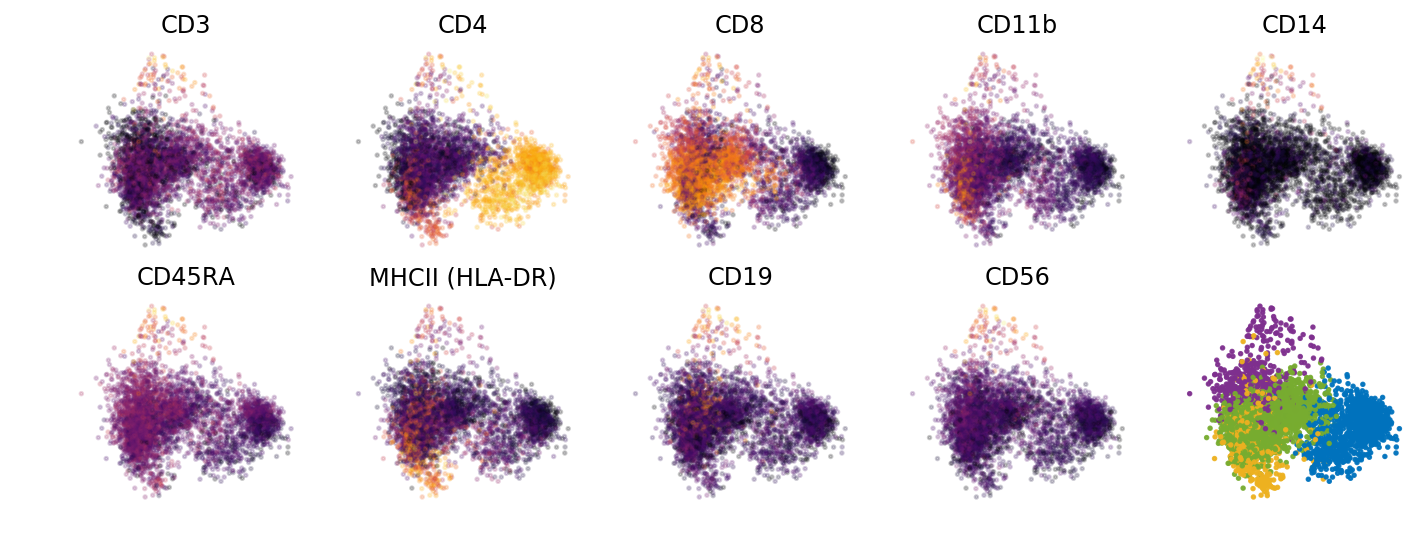


**Fig S40.** ECCITE-seq ctrl cell surface marker abundance and cluster definitions. Marker-specific plot dot color identifies marker abundance (yellow is high, black is low). Cluster plot dot color identifies cell type (blue: CD4+ T, yellow: monocytes, green: CD8+ T, purple: natural killer). Embedding: Protein PC2/3.


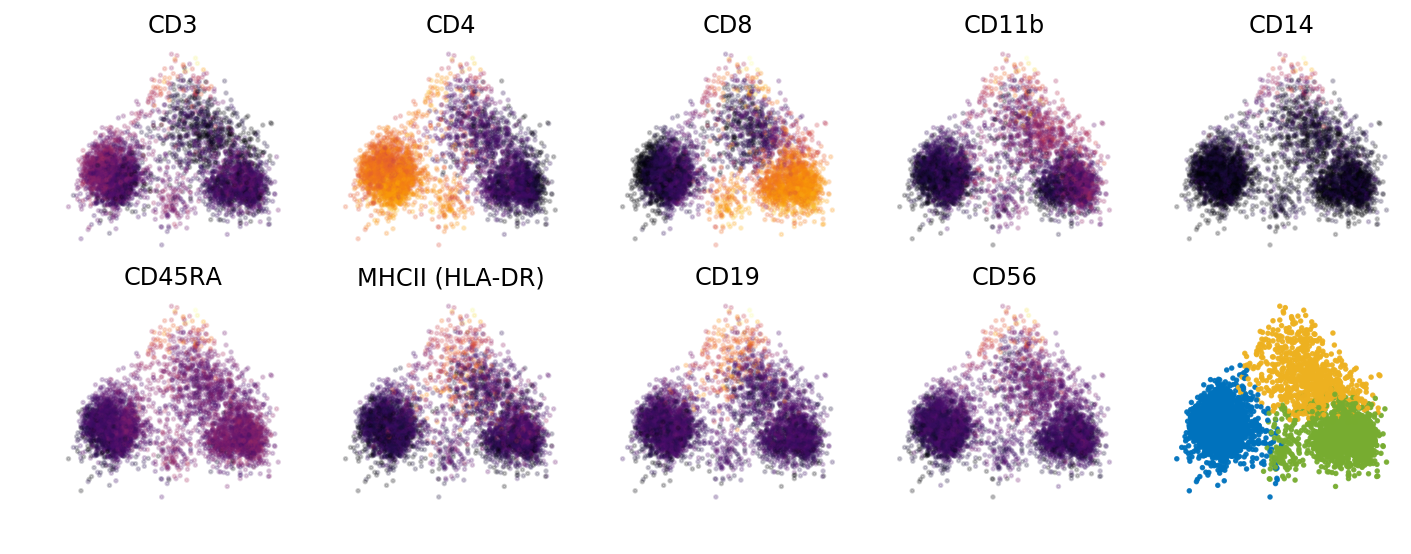


**Fig S41.** ECCITE-seq CTCL cell surface marker abundance and cluster definitions. Marker-specific plot dot color identifies marker abundance (yellow is high, black is low). Cluster plot dot color identifies cell type (blue: CD4+ T, red: B, yellow: monocytes, green: CD8+ T). Embedding: Protein PC1/2.


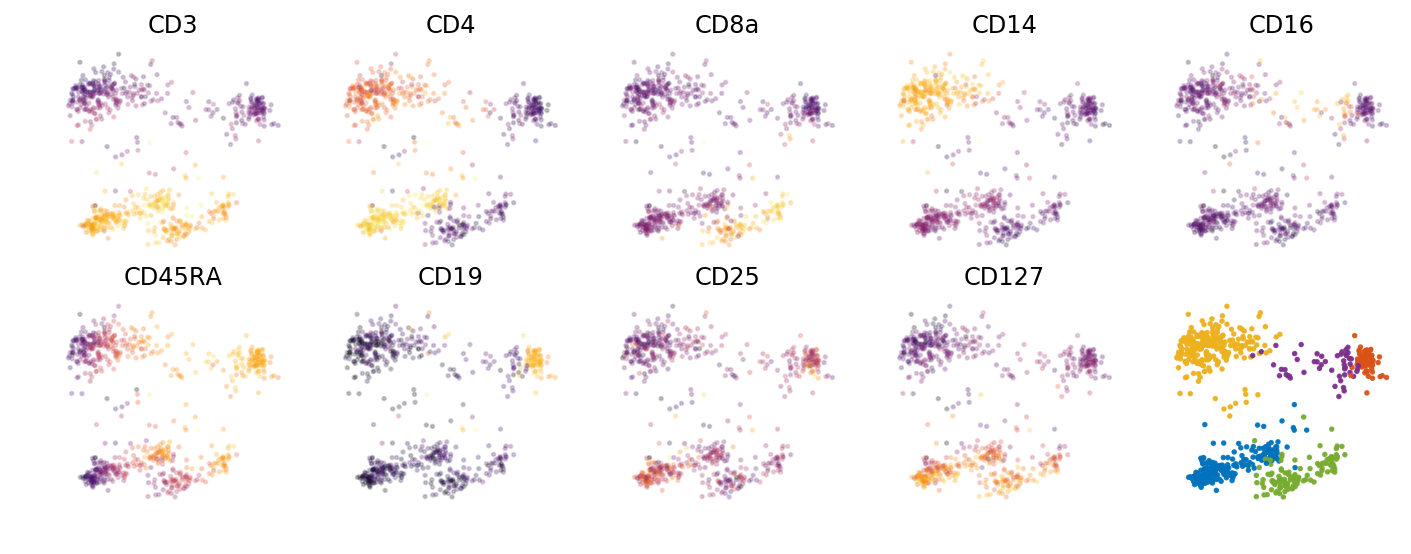


**Fig S42.** 10X 1k cell surface marker abundance and cluster definitions. Marker-specific plot dot color identifies marker abundance (yellow is high, black is low). Cluster plot dot color identifies cell type (blue: CD4+ T, red: B, yellow: monocytes, green: CD8+ T, purple: natural killer). Embedding: Protein PC1/2.


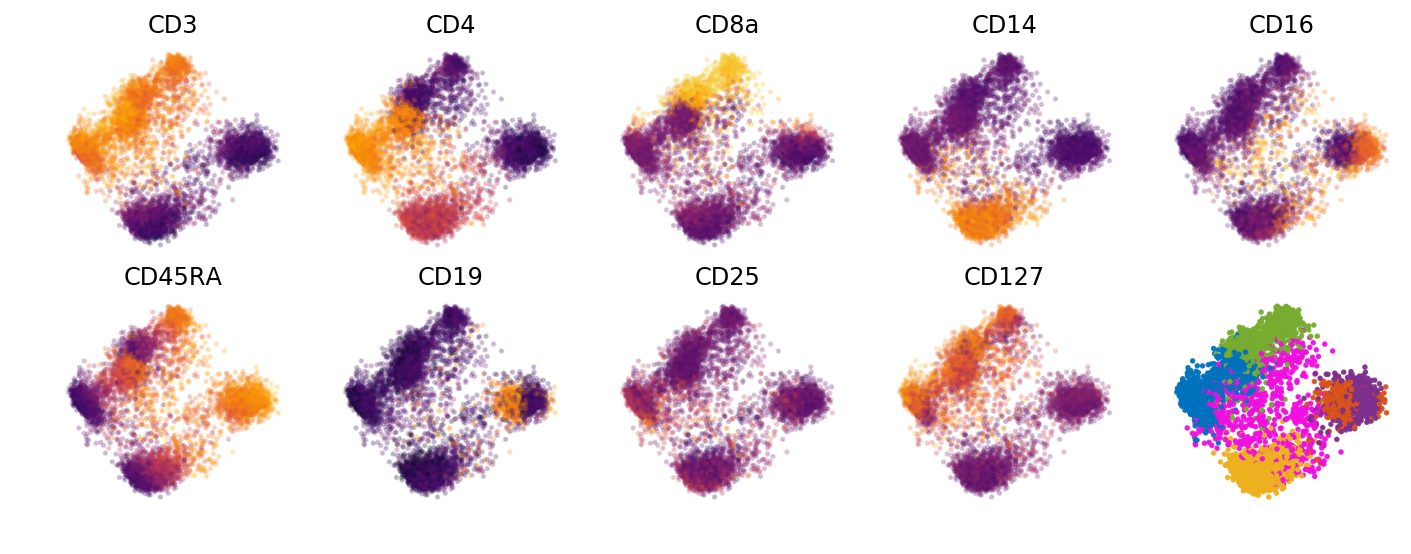


**Fig S43.** 10X 10k cell surface marker abundance and cluster definitions. Marker-specific plot dot color identifies marker abundance (yellow is high, black is low). Cluster plot dot color identifies cell type (blue: CD4+ T, red: B, yellow: monocytes, green: CD8+ T, purple: natural killer, pink: not identifiable unambiguously). Embedding: Protein PC1/2.

## Validation


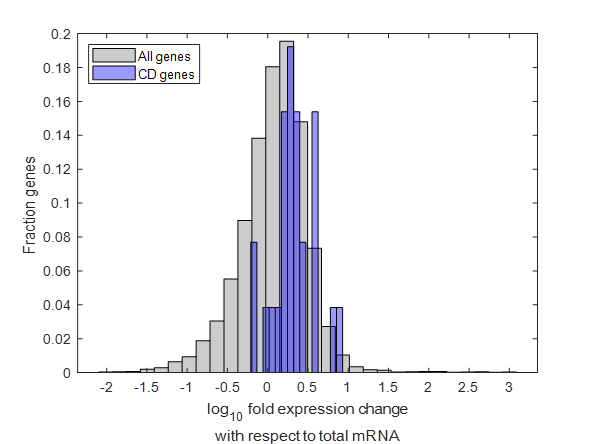


**Fig S44**. Distributions of log-fold expression changes across the entire transcriptome and across CD-coding genes (6).


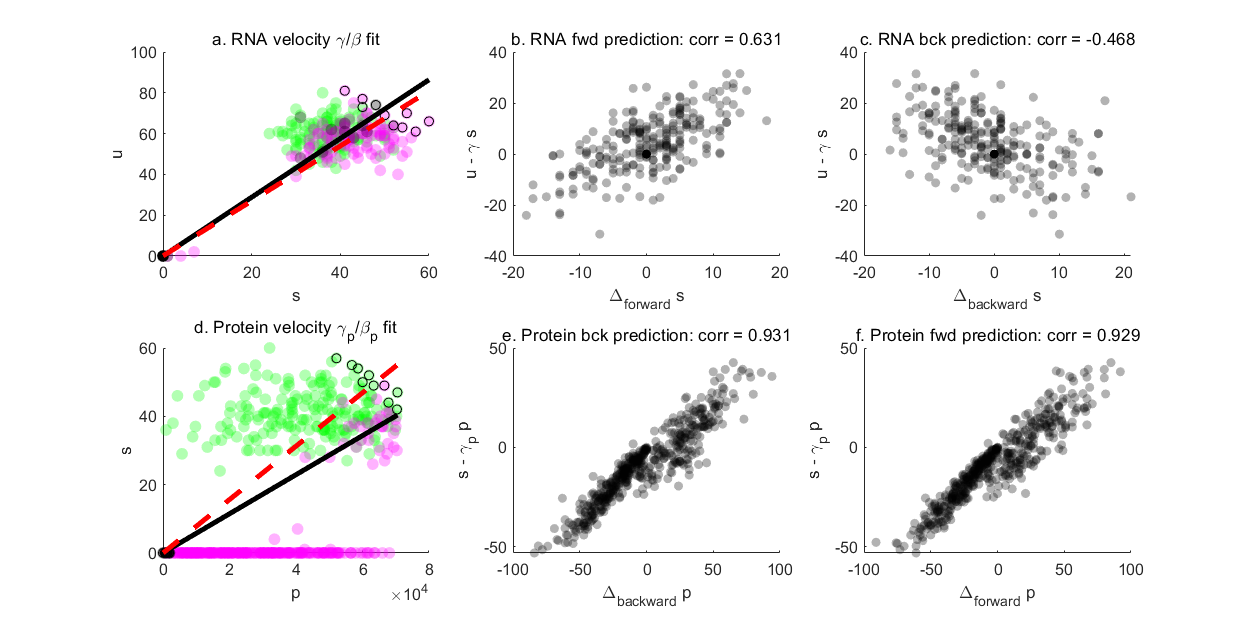


**Fig S45.** Inference and extrapolation performance for high $P_{on}$. Simulation parameters: $k_{ini}=59, k_{on}=k_{off}=1.8\times{10}^{-3},\beta=1,\gamma=1.44, \beta_{p}=2.58, \gamma_{p}= 1.5\times{10}^{-3}$. (a) RNA velocity fit results (green points: cells with higher spliced counts at next time point, magenta points: cells with lower spliced counts at next time point, points with black outline: cells used for linear fit, black line: ground truth $\gamma/\beta$, red dashed line: estimate of $\gamma/\beta$ from fit). (b) Comparison of ground truth forward difference in spliced counts and the RNA velocity prediction (points: cells). (c) Comparison of ground truth backward difference and the RNA velocity prediction (points: cells). (d) Protein velocity fit results (green points: cells with lower protein counts at previous time point, magenta points: cells with higher protein counts at previous time point, points with black outline: cells used for linear fit, black line: ground truth $\gamma_{p}/\beta_{p}$, red dashed line: estimate of $\gamma_{p}/\beta_{p}$ from fit). (e) Comparison of ground truth backward difference in protein counts and the protein velocity prediction (points: cells). (f) Comparison of ground truth forward difference and the protein velocity prediction (points: cells).


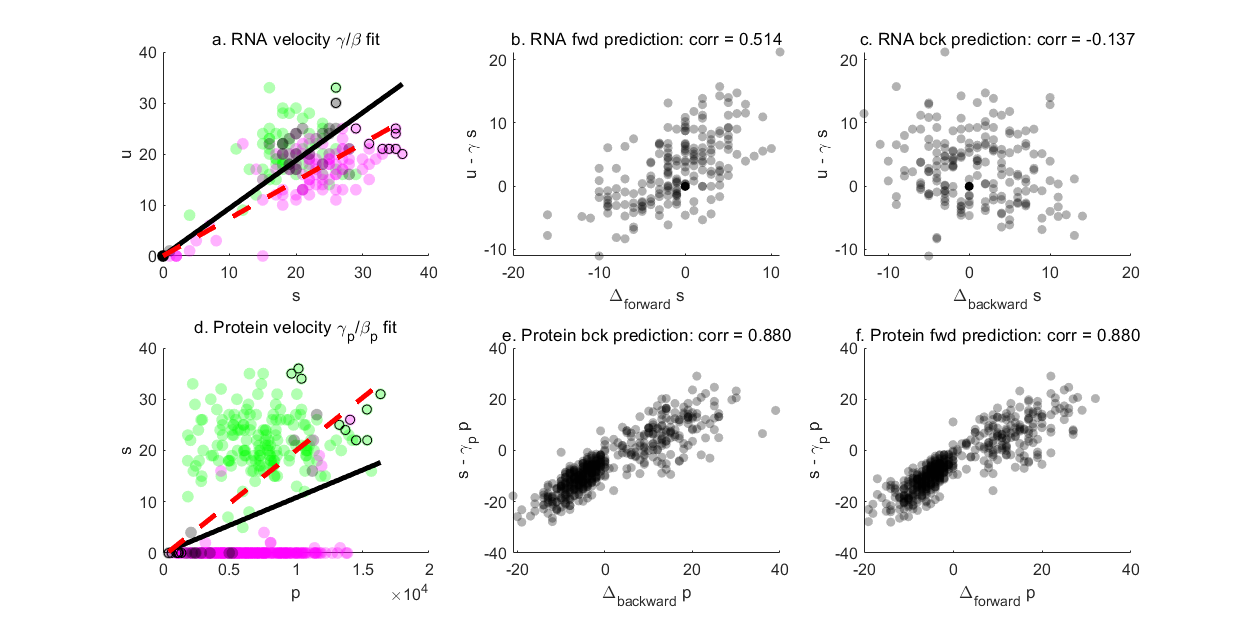


**Fig S46.** Inference and extrapolation performance for typical $P_{on}$. Simulation parameters: $k_{ini}=20, k_{on}=2.8\times{10}^{-3},k_{off}=5.8\times{10}^{-3},\beta=1,\gamma=0.94, \beta_{p}=0.92, \gamma_{p}= 1.0\times{10}^{-3}$. 500 cells per simulation. (a) RNA velocity fit results (green points: cells with higher spliced counts at next time point, magenta points: cells with lower spliced counts at next time point, points with black outline: cells used for linear fit, black line: ground truth $\gamma/\beta$, red dashed line: estimate of $\gamma/\beta$ from fit). (b) Comparison of ground truth forward difference in spliced counts and the RNA velocity prediction (points: cells). (c) Comparison of ground truth backward difference and the RNA velocity prediction (points: cells). (d) Protein velocity fit results (green points: cells with lower protein counts at previous time point, magenta points: cells with higher protein counts at previous time point, points with black outline: cells used for linear fit, black line: ground truth $\gamma_{p}/\beta_{p}$, red dashed line: estimate of $\gamma_{p}/\beta_{p}$ from fit). (e) Comparison of ground truth backward difference in protein counts and the protein velocity prediction (points: cells). (f) Comparison of ground truth forward difference and the protein velocity prediction (points: cells).


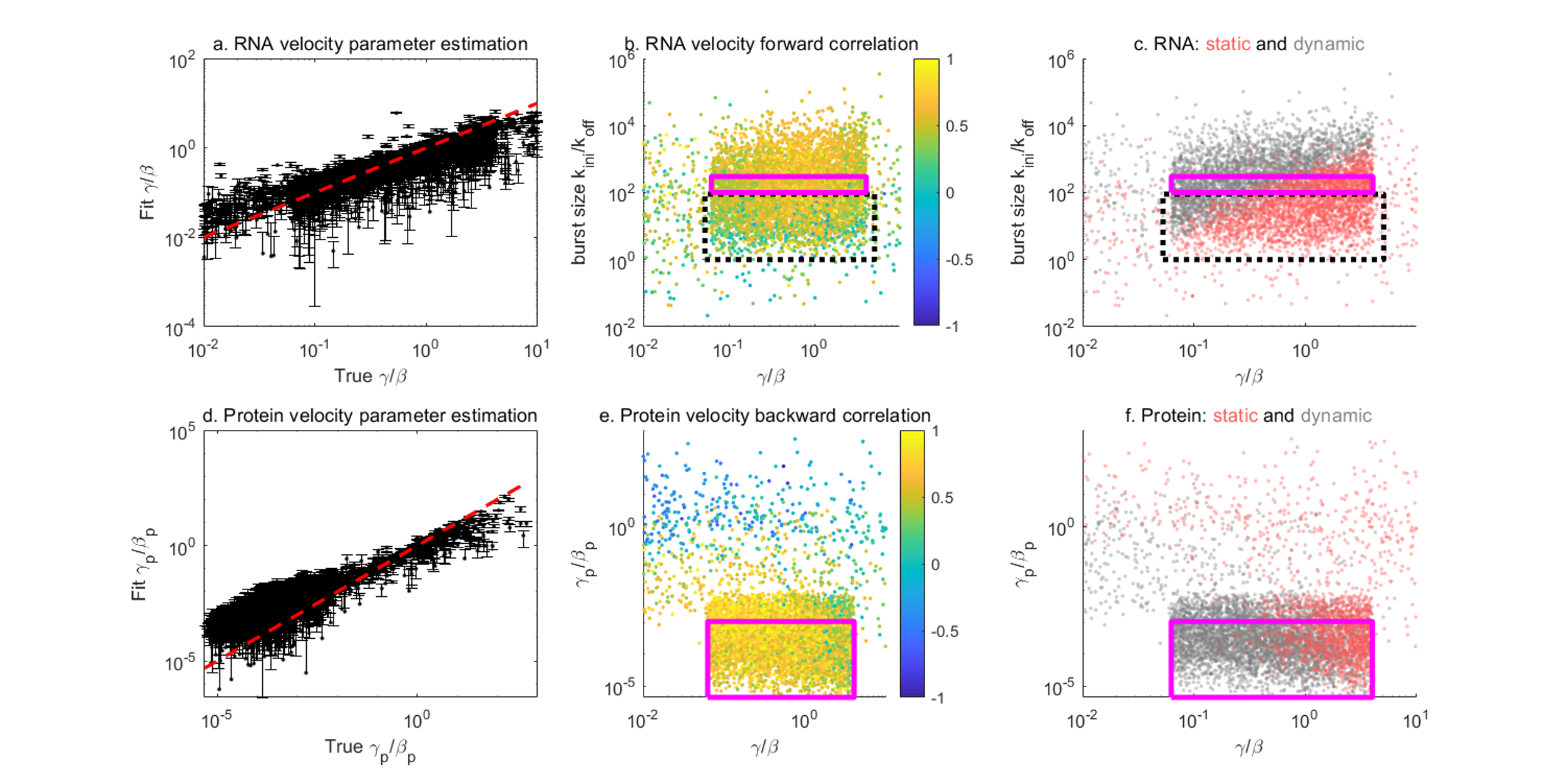


**Fig S47**. Inference and extrapolation performance throughout the parameter domain. 6,393 simulations, 500 cells per simulation. (a) Performance of RNA velocity $\gamma/\beta$ parameter inference (black points: parameter estimates for each simulation, error bars: 95% confidence interval for slope fit, red dashed line: ground truth). (b) Performance of RNA velocity forward extrapolation (points: parameter sets used in simulations, color: Pearson correlation given by color bar, magenta box: space of physiologically relevant parameters, black dotted box: space of parameters explored in RNA velocity validation [1]). (c) Parameter space regions qualitatively considered “static” and “dynamic” in RNA counts, classified based on $e=\left\langle\left( s_{t+1}-s_{t} \right)^{2} \right\rangle$ (red points: static, $e<1$, grey points: dynamic, $e>1$) (d) Performance of protein velocity $\gamma_{p}/\beta_{p}$ parameter inference (black points: parameter estimates for each simulation, error bars: 95% confidence interval for slope fit, red dashed line: ground truth). (e) Performance of protein velocity backward extrapolation (points: parameter sets used in simulations, color: Pearson correlation given by color bar, magenta box: space of physiologically relevant parameters). (f) Parameter space regions qualitatively considered “static” and “dynamic” in protein counts, classified based on $e=\left\langle\left( p_{t}-p_{t-1} \right)^{2} \right\rangle$ (red points: static, $e<1$, grey points: dynamic, $e>1$).


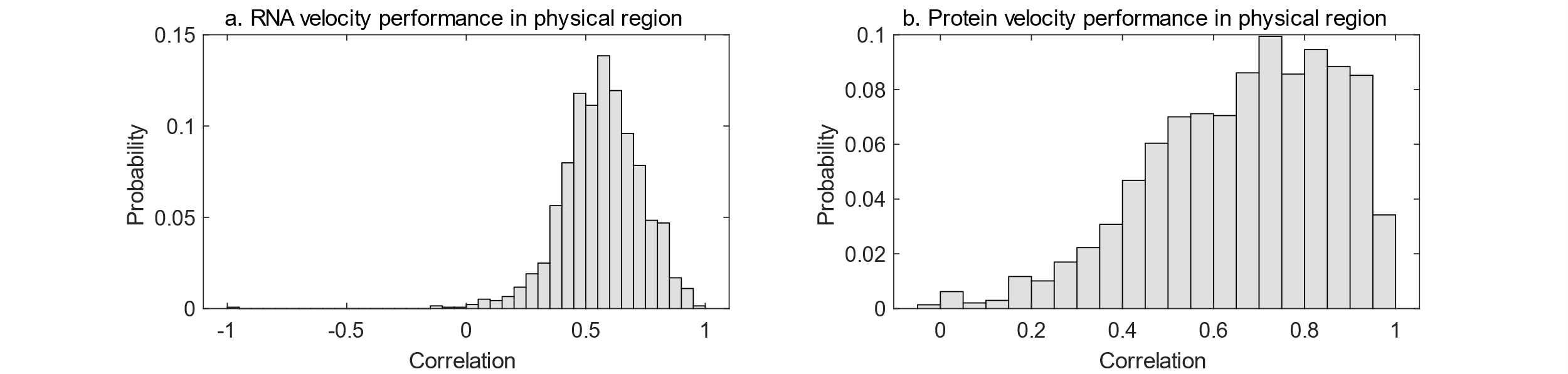


**Fig S48.** Extrapolation performance in the physiologically relevant parameter regions. 1,365 simulations for RNA velocity, 4,356 simulations for protein velocity, with 500 cells per simulation. (a) RNA velocity correlation distribution. (b) Protein velocity correlation distribution.


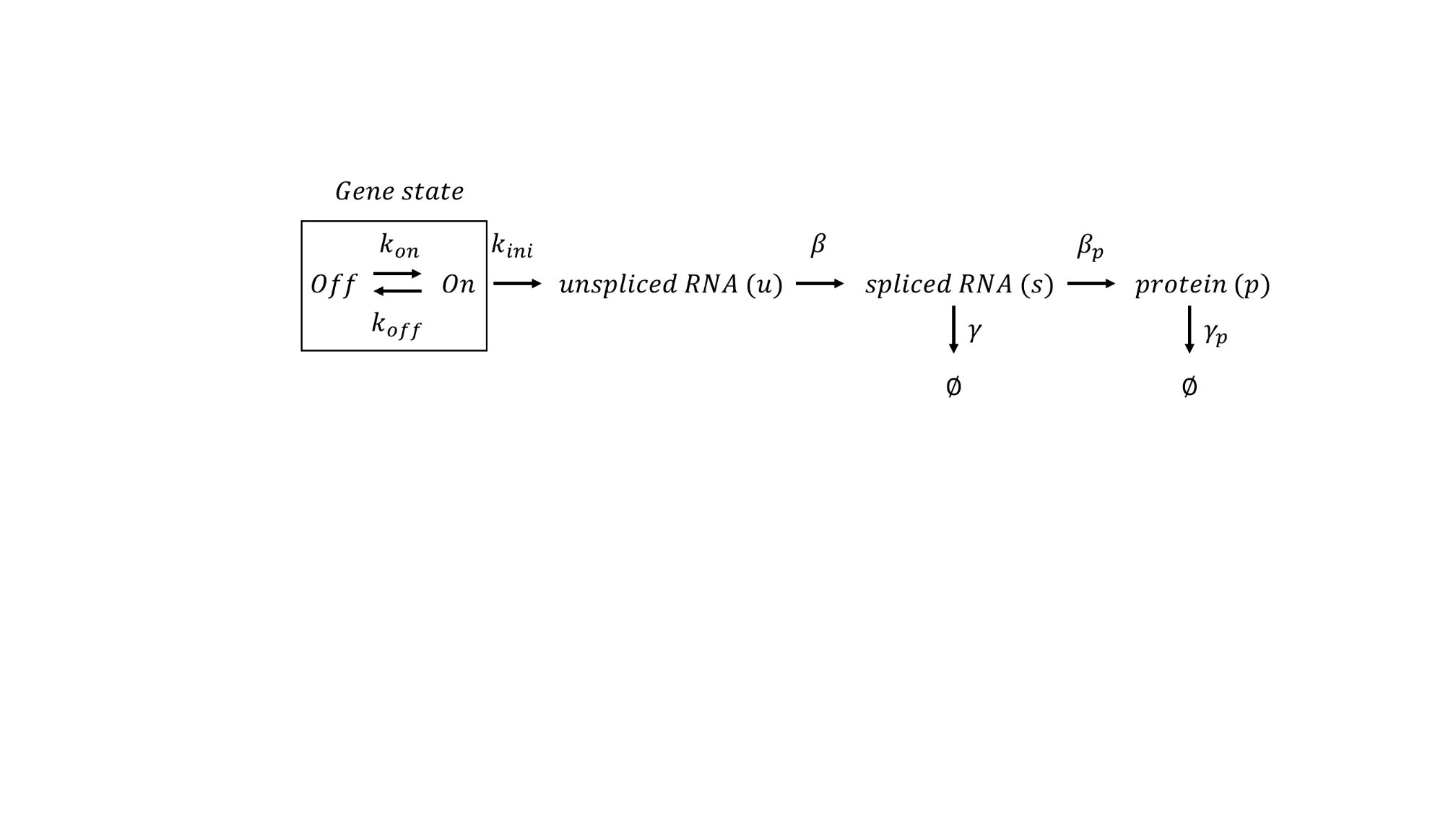


**Fig S49.** Stochastic simulation model and parametrization.


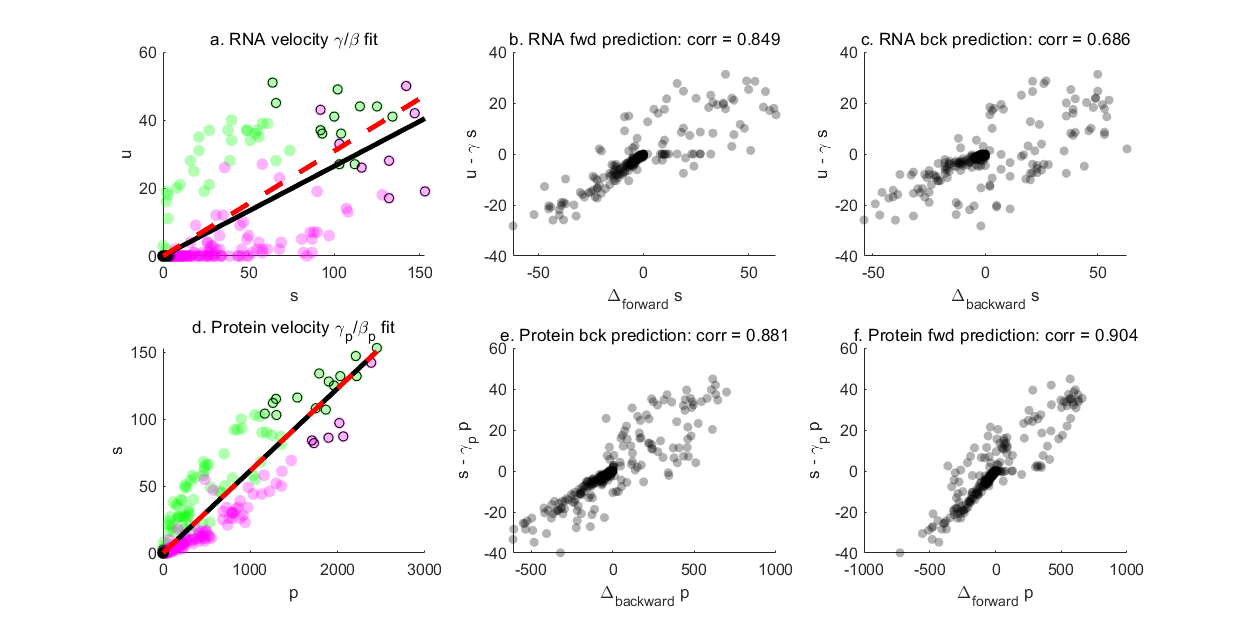


**Fig S50.** Inference and extrapolation performance for high $k_{on}$ and $\gamma_{p}$. Simulation parameters: $k_{ini}=39, k_{on}=1.2\times{10}^{-2},k_{off}=0.2,\beta=1,\gamma=0.26, \beta_{p}=6.8, \gamma_{p}= 0.41$. 1000 cells per simulation. (a) RNA velocity fit results (green points: cells with higher spliced counts at next time point, magenta points: cells with lower spliced counts at next time point, points with black outline: cells used for linear fit, black line: ground truth $\gamma/\beta$, red dashed line: estimate of $\gamma/\beta$ from fit). (b) Comparison of ground truth forward difference in spliced counts and the RNA velocity prediction (points: cells). (c) Comparison of ground truth backward difference and the RNA velocity prediction (points: cells). (d) Protein velocity fit results (green points: cells with lower protein counts at previous time point, magenta points: cells with higher protein counts at previous time point, points with black outline: cells used for linear fit, black line: ground truth $\gamma_{p}/\beta_{p}$, red dashed line: estimate of $\gamma_{p}/\beta_{p}$ from fit). (e) Comparison of ground truth backward difference in protein counts and the protein velocity prediction (points: cells). (f) Comparison of ground truth forward difference and the protein velocity prediction (points: cells).


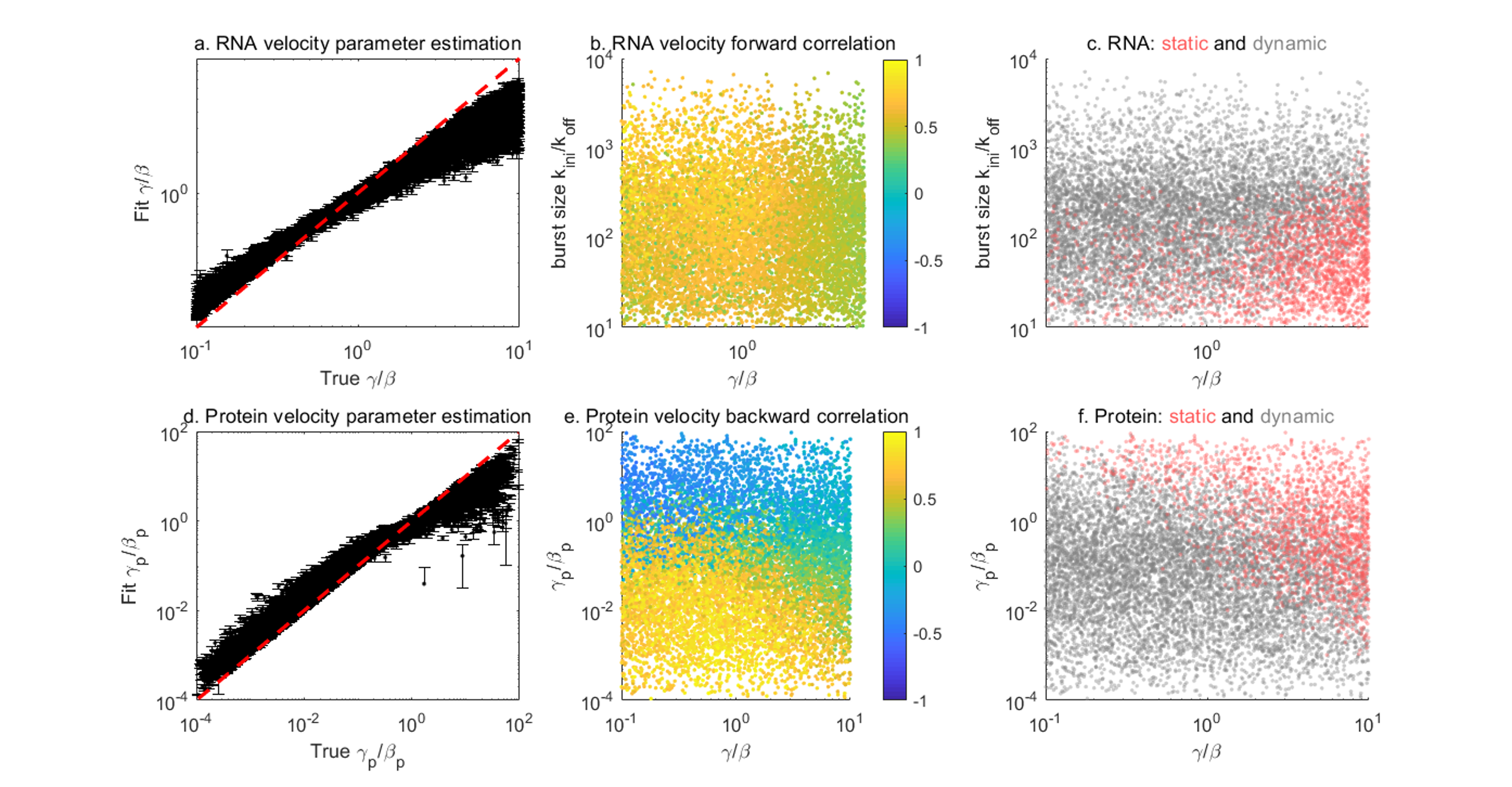


**Fig S51.** Inference and extrapolation performance throughout the parameter domain. 10,000 simulations, 2,000 cells per simulation. (a) Performance of RNA velocity $\gamma/\beta$ parameter inference (black points: parameter estimates for each simulation, error bars: 95% confidence interval for slope fit, red dashed line: ground truth). (b) Performance of RNA velocity forward extrapolation (points: parameter sets used in simulations, color: Pearson correlation given by color bar). (c) Parameter space regions qualitatively considered “static” and “dynamic” in RNA counts, classified based on $e=\left\langle\left( s_{t+1}-s_{t} \right)^{2} \right\rangle$ (red points: static, $e<1$, grey points: dynamic, $e>1$) (d) Performance of protein velocity $\gamma_{p}/\beta_{p}$ parameter inference (black points: parameter estimates for each simulation, error bars: 95% confidence interval for slope fit, red dashed line: ground truth). (e) Performance of protein velocity backward extrapolation (points: parameter sets used in simulations, color: Pearson correlation given by color bar). (f) Parameter space regions qualitatively considered “static” and “dynamic” in protein counts, classified based on $e=\left\langle\left( p_{t}-p_{t-1} \right)^{2} \right\rangle$ (red points: static, $e<1$, grey points: dynamic, $e>1$).


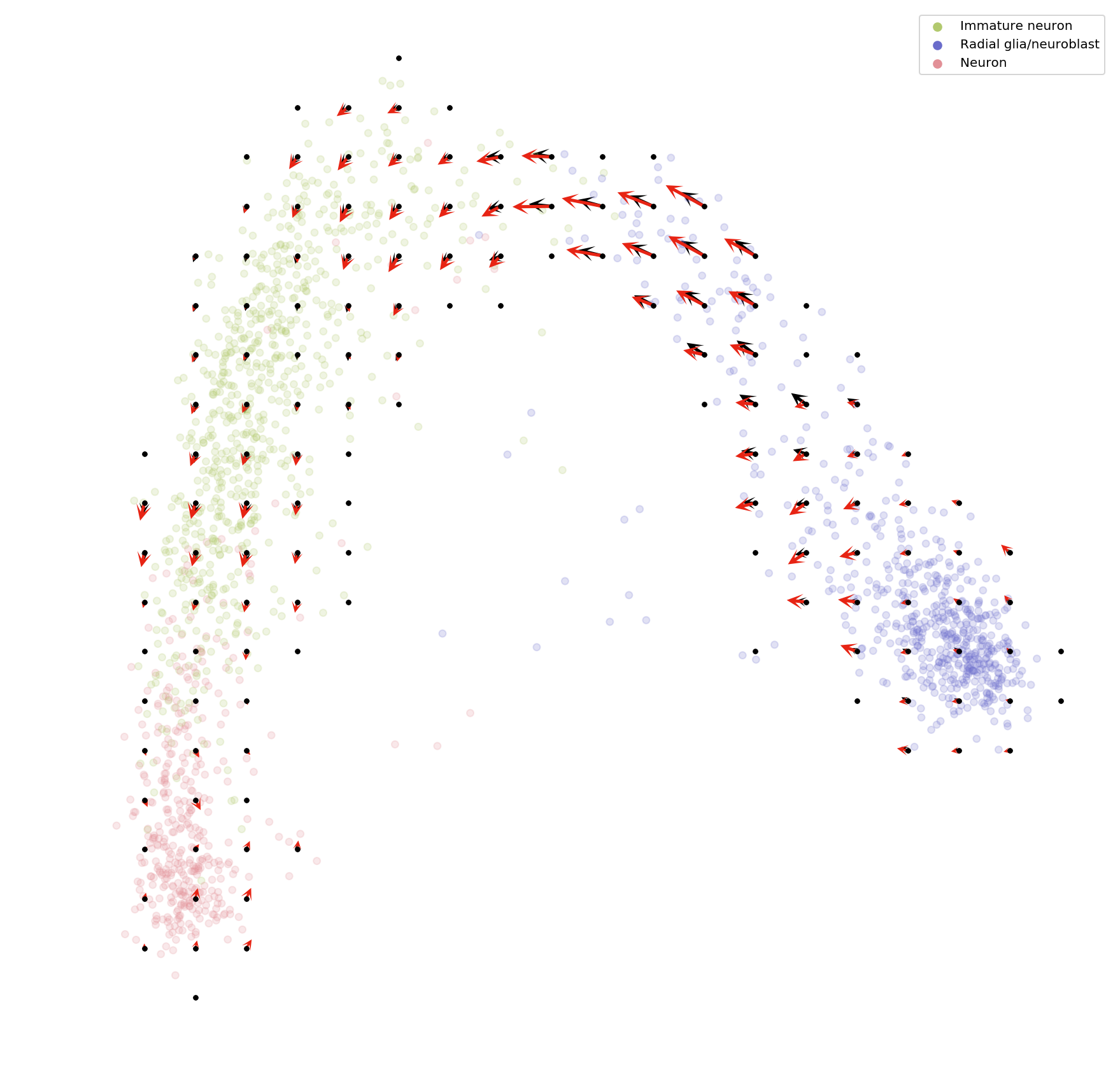


**Fig S52**. RNA velocity on the glutamatergic neurogenesis differentiation landscape (1), calculated using standard and Boolean methods. Arrow color identifies RNA velocity estimate (standard: black, Boolean: red). Dot color identifies cell type (blue: radial glia/neuroblast, green: immature neuron, red: neuron). Embedding: PC1/2.


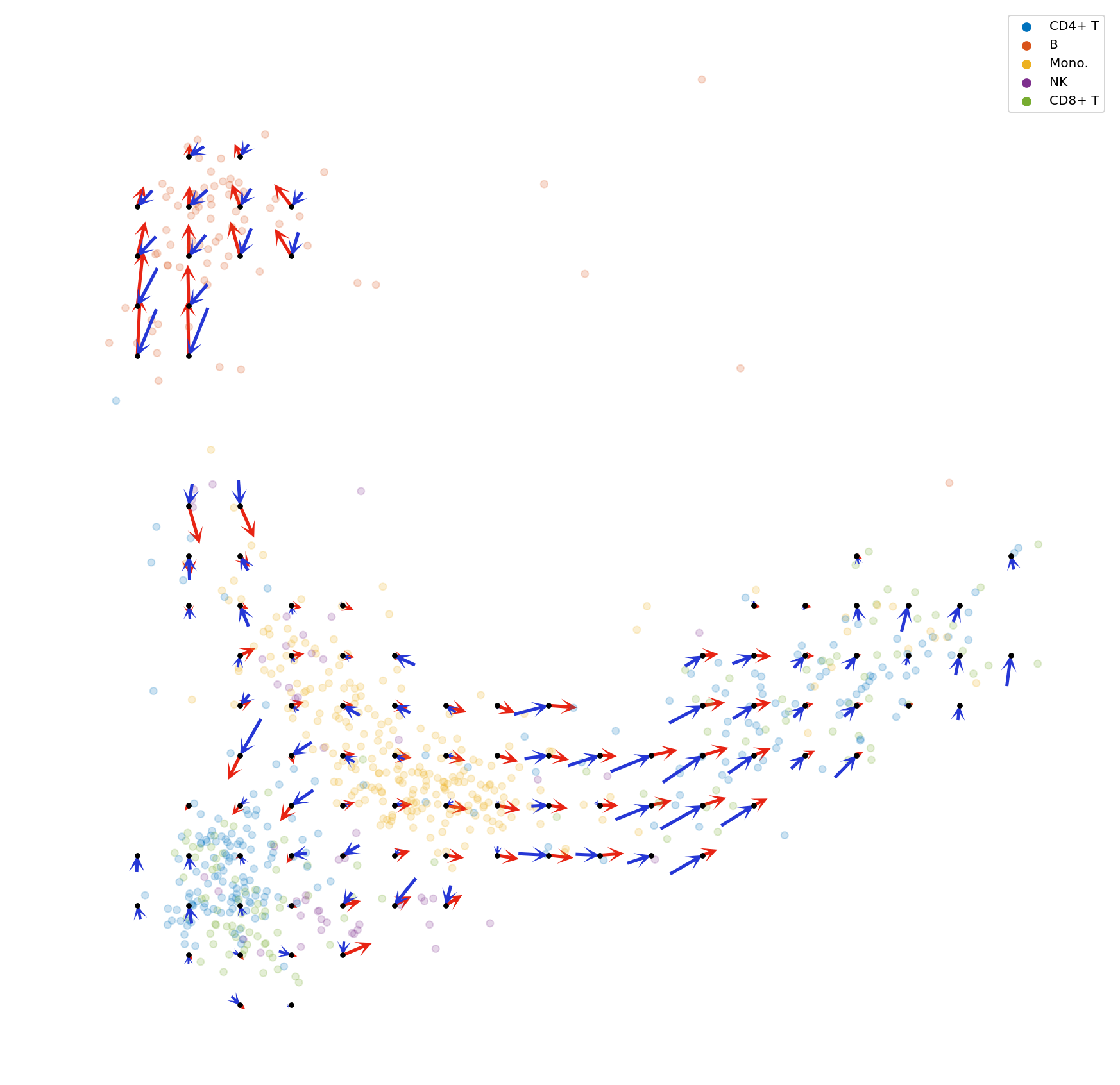


**Fig S53**. 10X 1k velocity fields visualized on a grid, calculated using standard method. Arrow color identifies velocity estimate (RNA: red, protein: blue). Dot color identifies cell type (blue: CD4+ T, red: B, yellow: monocytes, green: CD8+ T, purple: natural killer). Embedding: PC2/3.


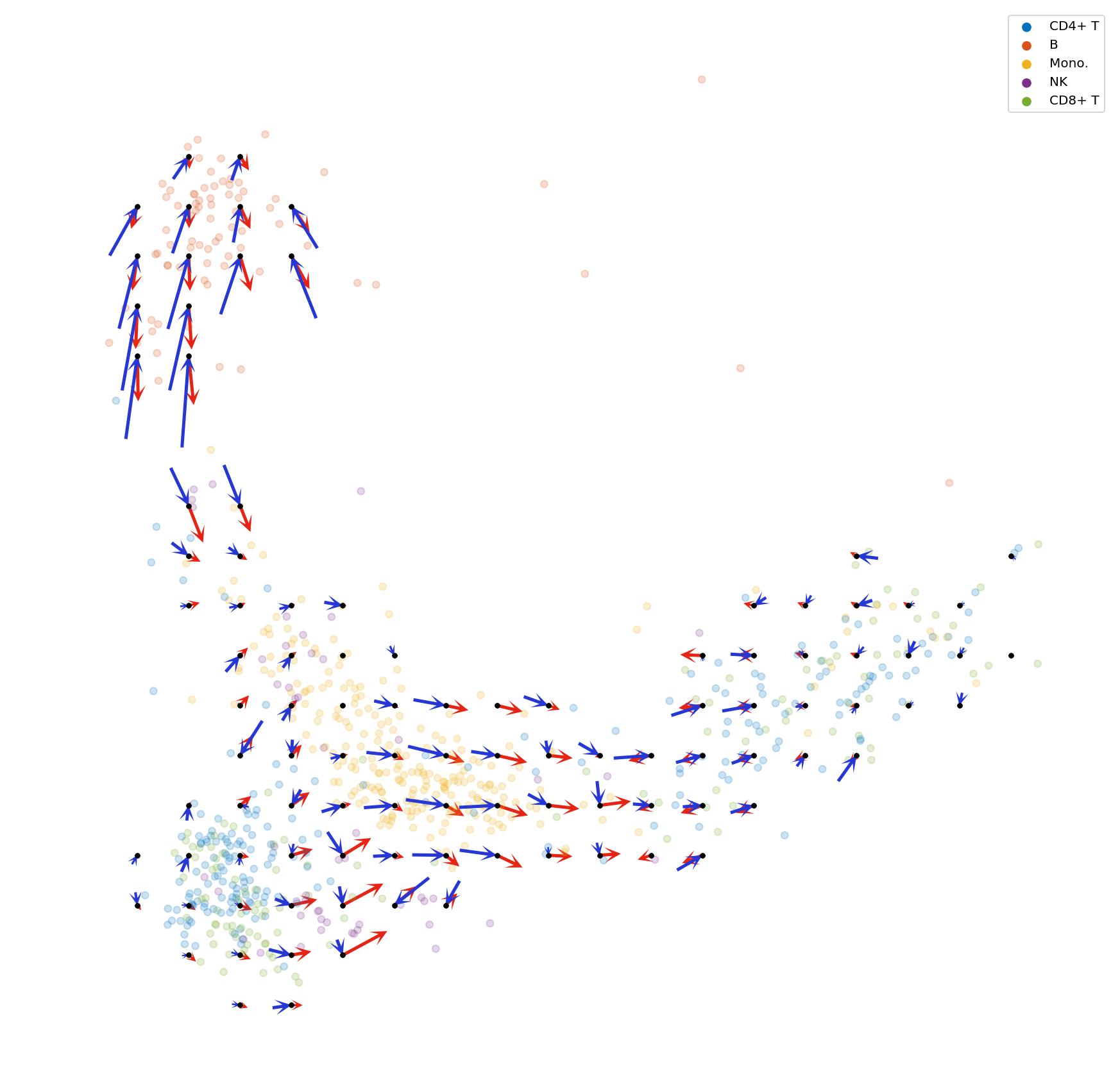


**Fig S54**. 10X 1k velocity fields visualized on a grid, calculated using Boolean method. Arrow color identifies velocity estimate (RNA: red, protein: blue). Dot color identifies cell type (blue: CD4+ T, red: B, yellow: monocytes, green: CD8+ T, purple: natural killer). Embedding: PC2/3.


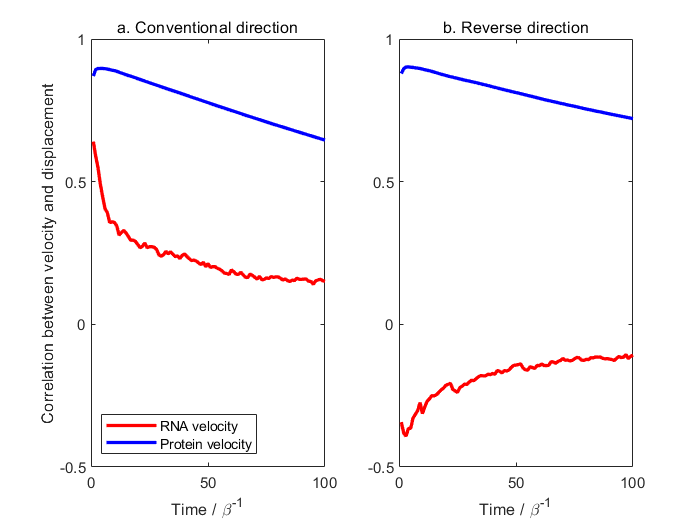


**Fig S55**. Extrapolation performance for typical $P_{on}$, as a function of time horizon. Simulation parameters: $k_{ini}=20, k_{on}=2.8\times{10}^{-3},k_{off}=5.8\times{10}^{-3},\beta=1,\gamma=0.94, \beta_{p}=0.92, \gamma_{p}= 1.0\times{10}^{-3}$. $5000$ cells, simulation until $4000\beta$. (a) Correlation between velocity estimate and the true displacement at a given time horizon; RNA velocity is compared to forward cell state and protein velocity is compared to backward cell state. (b) Correlation between velocity estimate and the true displacement at a given time horizon; RNA velocity is compared to *backward* cell state and protein velocity is compared to *forward* cell state, in a reversal of the standard use case.
