# Supplementary material for: Protein velocity and acceleration from single-cell multiomics experiments
Source: Genome Biol. 2020 Feb 18;21:39. doi: 10.1186/s13059-020-1945-3 (PMC7029606; doi:10.1186/s13059-020-1945-3)
Supplement: Supplementary file 2 — Additional file 2. Review history. [file 13059_2020_1945_MOESM2_ESM.docx]

Review History

**First round of review**

**Reviewer 1**

**Are you able to assess all statistics in the manuscript, including the appropriateness of statistical tests used?**

There are no statistics in the manuscript.

**Comments to author:**

Most current methods for single-cell measurements provide only static snapshots. Yet, estimating dynamics in single cells is very important. To some degree, such estimates may be derived by the RNA-velocity framework, and the authors suggest incorporating protein measurements into this framework. I like this idea. It appears very obvious to me, but it has not been realized, and thus I think it can serve as the basis of a powerful method and a great paper.

Despite liking the idea, I found its current implementation rather poor in terms of computational methodology, data used, and even description. The authors make unrealistic assumptions justified only by convenience for their approach. Specifically:

-- I am very uncomfortable assuming that "the translation rate is gene-independent." The protein / mRNA ratios for different genes span 3 - 4 orders of magnitude Wilhelm et al., doi: 10.1038/nature13319. Edfors et al., doi: 10.15252/msb.20167144 This means that \beta_p and \gamma_p in the supplemental equations can be different for different genes, with different values flipping the sign of dp/dt in the equation proposed by the authors. Thus without making this utterly unrealistic assumption, the equation proposed by the authors cannot predict even the direction of the protein derivative.

-- The paper is extremely terse and difficult to understand even for an expert. It needs to be expanded substantially for a broad audience journal. Assumptions are not mentioned, relevant scientific literature ignored, and the proposed new methods and are insufficiently explained. Also, the authors need to acknowledge many caveats, including but not limited to: 1) The protein estimates reflect only surface protein abundance (at best), not the total protein abundance. The mRNA levels reflect total mRNA abundance. 2) The effect of protein degradation rate -- many proteins are primarily regulated by protein degradation, not by protein synthesis Liu et al. doi: 10.1016/j.cell.2016.03.014. Thus, their protein levels are mostly independent from the RNA levels. This fact is very relevant to the author's model and should not be ignored. 3) Do the authors assume that antibodies bind their target proteins in 1:1 stoichiometry? What is the evidence for this assumption?

-- None of the results are validated. Also, they are not linked to plausible biological interpretation.

**Reviewer 2**

**Are you able to assess all statistics in the manuscript, including the appropriateness of statistical tests used?**

Yes, and I have assessed the statistics in my report.

**Comments to author:**

In this manuscript, Gorin and colleagues present a bioinformatic analysis framework under the name "protein acceleration" intended as a new tool to study single-cell multi-omics datasets that jointly measure gene expression and protein concentration.

The objective of this work is to extend the concepts presented by the Kharchenko group titled "RNA velocity" (La Manno et al., 2018). The idea behind "RNA velocity" is that by discriminating between nascent and processed transcripts in single-cell RNA-seq and considering a model of transcription, one can determine the rate of change of gene expression (i.e. velocity) in single cells. Note that, in a sense, this allows "extrapolation" of the future state of a cell. The authors effectively review this idea in a recent spotlight article (Svensson and Pachter, 2018).

Gorin and colleagues use the same line of arguments and propose that by collecting information on proteins and processed RNA, it is possible to fit a similar model and obtain insight into the dynamics of gene regulation. Note that, to make this possible, the authors need to assume that there is no separation of time scales for the processes of transcription and translation. This assumption, while a bit unorthodox, it is proved acceptable by at least some of the diagrams s-p.

The most useful contribution from the authors is to analyze, for the first time, RNA-protein diagrams with the explicit intent to derive information of single-cell dynamics on it. Another valuable take-home from the manuscript is the comparison between different techniques of joint RNA/protein quantification, including CITE-seq, REAP-seq, and ECCITE-seq. From this analysis, the authors identify the most promising technology.

The main output of the method proposed here is combining forward extrapolation with a backward extrapolation to allow a visualization of a curved cellular trajectory. The curve is representing the effect on the acceleration on changing the trajectory. Unfortunately, we think, in this fundamental step, there hides a fundamental mistake (Major concern 1) in the paper that invalidates the interpretation of those plots. This is only one of the concerns that we have on the paper.

We have profound esteem for the authors, and we were surprised to find both the presentation, accuracy of statements, and mathematical articulation of the manuscript underwhelming. We believe that the authors would have been capable of producing a high-quality manuscript, but that they probably missed out in the attempt to get this out quickly. I believe this work is far from mature, and the effort to fix it is far beyond the normal scope of a major revision.

Main concerns

1) The authors present a model that allows extrapolation of the protein abundance and RNA abundance by a first-order approximation either into the past or future. However, the extrapolation is only possible in the respective spaces. The authors seem very confused about this point. This is evident from the fact that they visualize both protein velocity and RNA velocity on the same manifold 2d representation and then completely misinterpret what the picture shows. Doing this is completely circular, and if the RNA and protein space overlap, then there is no protein velocity information and we are at steady-state!

2) The "RNA velocity" (first derivative of mRNA levels) in a sense IS the "protein acceleration," (second derivative of protein levels ~= first derivative of mRNA levels) and the authors are considering a system where they have access to both protein velocity and RNA velocity protein acceleration. While in some sections, the authors seem to get this right, there is a general confusion in the paper.

The confusion on (1) and (2) is highlighted by the four poorly qualified statements that end up being contradictory:

- "The combination of protein velocities reveals the curvature of the cell state landscape." It is the RNA velocity that is the curvature of the protein space!

- "With respect to the reference frame of unspliced transcripts, the protein inferences correspond to second-order protein acceleration of unspliced transcript counts." This is not true, it is instead the other way around.

- "We visualize cell movement in the spliced mRNA using a Bézier curve calculated from three points corresponding to past, present, and future." they represent the present and the future of the RNA levels but the past of the protein level!

- "Our method estimates protein translation dynamics from a single timepoint." This is an overstatement, to understand the translation dynamics one would need to estimate all the parameters from a cell population (a subset of which should be sampled at steady state).

3) At the implementation level, it seems that the authors are just adapting, with minimal changes, the code from the package velocyto (La Manno et al., 2018). From inspection, I noticed a trivial substitution of variables, and some of the code confirms that points (1-2) are a key interpretation problem. This is further apparent from the fact that the pipeline is implemented solely in a Jupyter notebook rather than as a package or scanpy or velocyto extension.

4) It is not clear to me what we learn from acceleration. The authors should illustrate an example where knowing the curvature of the manifold can be somehow related to useful biological conclusions. For example: considering a bifurcation of a progenitor cell type to two alternative linages, can the author's approach help exploring the possible regulatory causes of the bifurcation?

5) Unfortunately, the fact that the authors use publicly available datasets impairs their ability to select a system where a "protein acceleration" might be more relevant and allow them to validate what they observe. I believe that in this scenario there is no way to tell if the predictions are reasonable.

6) The paper does not present any sensitivity analysis or simulation based data. Note that having a situation with simulated data might also help to understand what is the parameter distribution and in which part of the distribution inference is accurate.

La Manno, G., Soldatov, R., Zeisel, A., Braun, E., Hochgerner, H., Petukhov, V., Lidschreiber, K., Kastriti, M.E., Lönnerberg, P., Furlan, A., et al. (2018). RNA velocity of single cells. Nature 560, 494-498.

Svensson, V., and Pachter, L. (2018). RNA Velocity: Molecular Kinetics from Single-Cell RNA-Seq. Mol. Cell 72, 7-9.

**Reviewer 3:**

**Are you able to assess all statistics in the manuscript, including the appropriateness of statistical tests used?**

There are no statistics in the manuscript.

**Comments to author:**

Gorin et al. build on previously established RNA velocity methods and develop a method that captures unspliced, spliced, and protein production kinetics from single-cell multi-omics data. The authors acquired GEO datasets from CITE-seq, REAP-seq, and ECCITE-seq experiments, to predict future states of cells by inferring kinetic trajectories of single cells from peripheral blood mononuclear cell (PBMC) datasets.

Overall, this study provides a novel analysis in the recently emerging RNA velocity field. Specifically, the authors expand on current methods incorporating protein abundance data from single-cell multi-omics data to be able to infer the past state of cells, in addition to the present and future state inferred from RNA transcript abundance. Secondly, the data acquired in this current study utilizes single-cell data, allowing one to capture heterogeneity in a population of cells. Finally, previous RNA velocity papers involved measuring RNA abundances at several time points in the experiment. The present analysis requires information from a single time point. For these reasons, I believe this study is suitable for publication, with minor revisions indicated below.

*	(Page 3) "for RNA velocity, we used a broad panel of genes with robust unspliced detection" - were all genes indicated in the supplemental figure considered 'robustly detected'? There are a few genes that have very little detection.

*	(Page 3) "A subset of high-abundance gene/protein pairs were used…" How were these genes specifically selected, or was it random? Additionally, "we used a broad panel of genes with robust unspliced detection" - a list of these genes should be present in the supplementary table. A table for protein velocity is presented, but not for the RNA velocity measurements.

*	(Figure 1) The diagram is intuitive and easy to read. However, the authors state that a "broad panel of genes" with "robust unspliced detection" were selected for study in this paper. Would readers benefit from one example gene being shown with actual data in the same manner as figure 1B?

*	(Page 5) "This behaviour may reflect recent findings [12] that describe mRNA transcript "pile-up" due to heavily suppressed translation…" - this might explain the strong protein acceleration in T lymphocytes. Can the authors speculate on B cells exhibiting high protein acceleration?

*	(Page 5) "The unidirectional monocyte velocity suggests response or plasticity" - Could this point be elaborated? Which response might be elicited? Or rather, what scenario could result in the observed monocyte velocity? The reference associated with this statement also refers to T-cell lineage commitment and plasticity.

*	(Page 5) "…partitioning into static and mobile populations…" - Would it benefit readers if the static and mobile populations were labeled in the supplementary figure?

*	(Page 6) "Future work may involve better measurements…" - of what? Could a specific example be given?

*	(Supplementary Fig. 19 & 20) The arrow and arrow colour are not easily distinguished from the velocity fields.

**Authors Response**

**Point-by-point responses to the reviewers’ comments:**

Responses to Reviewer #1

Most current methods for single-cell measurements provide only static snapshots. Yet, estimating dynamics in single cells is very important. To some degree, such estimates may be derived by the RNA-velocity framework, and the authors suggest incorporating protein measurements into this framework. I like this idea. It appears very obvious to me, but it has not been realized, and thus I think it can serve as the basis of a powerful method and a great paper.

Despite liking the idea, I found its current implementation rather poor in terms of computational methodology, data used, and even description. The authors make unrealistic assumptions justified only by convenience for their approach. Specifically:

-- I am very uncomfortable assuming that "the translation rate is gene-independent." The protein / mRNA ratios for different genes span 3 - 4 orders of magnitude Wilhelm et al., doi: 10.1038/nature13319. Edfors et al., doi: 10.15252/msb.20167144 This means that and in the supplemental equations can be different for different genes, with different values flipping the sign of dp/dt in the equation proposed by the authors.

*We expect to be different, and fit it on a per-gene basis. We assume to be unity. This assumption is analogous to the assumption of unity in the original RNA velocity publication. We provide an outline of significant assumptions within the estimation framework and explain why they are equivalent to or better than assumptions in the original publication in Supplementary Table 3. Furthermore, a study by Xiao and Wu (DOI: 10.1021/acs.analchem.7b02241) suggests that glycoprotein synthesis rates are fairly similar, so we posit that assuming unity translation rates for this protein panel is at least as valid as assuming unity splicing rates. As we discuss in the Quantitative considerations and assumptions section of the Supplementary Note:*

*“Since both splicing (2) and translation (3) are very tightly regulated in eukaryotes, the assumption of constant rates across all cells and genes means the extrapolation step is qualitative. Broadly speaking, assuming similar translation rates for the cell surface markers used in the feature barcoding technologies appears to be less problematic than assuming similar splicing rates for several thousand genes in the transcriptome, as suggested by the comparable synthesis rates determined for a panel of 847 glycoproteins in a recent study (4) and the widely dispersed splicing rates determined for a panel of 832 genes by La Manno et al. (1). Therefore, we expect the quality of the unity translation rate assumption to be roughly equivalent to or better than the unity splicing rate assumption crucial to the RNA velocity method.”*

Thus without making this utterly unrealistic assumption, the equation proposed by the authors cannot predict even the direction of the protein derivative.

*We fit , then estimate dp/dt. Since is positive, we can predict the direction of the protein derivative regardless of the assumption. The caveats and assumptions are described in detail in the Quantitative considerations and assumptions section of the Supplementary Note; as we summarize:*

*“Violations of the assumption of unity and make quantitative comparisons of velocity between different genes problematic. However, crucially, we can predict the direction of each velocity, since both and are positive in the physiology model.”*

-- The paper is extremely terse and difficult to understand even for an expert. It needs to be expanded substantially for a broad audience journal.

*We have included further methodological description and discussion in the Supplementary Note.*

Assumptions are not mentioned, relevant scientific literature ignored, and the proposed new methods and are insufficiently explained.

*The Quantitative considerations and assumptions section of the Supplementary Note, as well as Supplementary Table 3, enumerate the assumptions and describe their foundation in the assumptions of the original RNA velocity publication. As we describe:*

*“The inference of protein velocity requires a number of assumptions, some novel and some inherited from the RNA velocity workflow. We outline these assumptions and discuss their relative quality in Supplementary Table 3. The table is roughly divided to address assumptions about data quality, parameter values, the velocity calculation process, and the embedding process.”*

*We have supplemented the discussion of physiology, modeling, and simulation with substantial background information, as well as analyzed two additional datasets representing the widely relevant and recently commercialized method of feature barcoding. To select a single example, we highlight the new references in the physiological interpretation paragraph:*

*“We found that subsets of B cells and of T lymphocytes exhibit strong protein acceleration. We hypothesize that the B cell partitioning corresponds to the differences between cell subtypes, e.g. mature B cells, which are resting (12) and require dedicated T-cell activation (13), and plasma cells, which quickly respond to stimuli (14) and would be expected to have high acceleration on the relevant timescales. The T lymphocyte behavior may reflect recent findings that describe mRNA transcript “pile-up” due to heavily suppressed translation in naïve CD4+ T cells (15), or potential lymphocyte plasticity (16). The monocyte behavior may correspond to the steady-state partitioning between monocyte subtypes (17), such as the transition from classical to non-classical circulating monocytes (18). However, due to the imperfect separation of cell types in the embedding, we caution against over-interpretation of aggregated velocities.*

*The Velocity workflow and Embedding process sections of the Supplementary Note explain the method in extensive detail.*

Also, the authors need to acknowledge many caveats, including but not limited to: 1) The protein estimates reflect only surface protein abundance (at best), not the total protein abundance. The mRNA levels reflect total mRNA abundance.

*We describe the implications of this assumption in the Quantitative considerations and assumptions section of the Supplementary Note. Essentially, we assume that the fraction of surface proteins retained within the cell is negligible, especially considering the length of their lifetimes (on the order of days). However, our analysis of approximation error shows that even if a modest fraction remains in the cell, the estimate quality is not degraded. As we describe:*

*“By the same argument, we posit that any modest bias in the protein counts is insubstantial. For example, if fraction of proteins is retained in the cell and thus not observable, and must be expressed in units of . As long as is , the error of this estimate is still , no worse than the original estimate. For the purposes of our analysis, we assume .”*

2) The effect of protein degradation rate -- many proteins are primarily regulated by protein degradation, not by protein synthesis Liu et al. doi: 10.1016/j.cell.2016.03.014. Thus, their protein levels are mostly independent from the RNA levels. This fact is very relevant to the author's model and should not be ignored.

*We do see a number of proteins whose phase portraits do not match the model (all proteins without linear fits in Supplementary Figures 1-6; most prominently, CD4 in Figure 2b). We hypothesize that their behavior corresponds to the regulation of protein degradation, as in the discussion of Figure 2b. However, in this study, we select proteins with phase portraits that do match the model, analogously to the standard velocyto package selecting genes with linear spliced/unspliced phase portraits. The analysis of genes with more complex behavior is outside the scope of the current publication, although we hypothesize that they could be fruitfully used for inference in future investigations. The article by Liu et al. does state “At Steady State, mRNA Levels Primarily Explain Protein Levels;” our steady-state PBMC system, as well as model assumptions makes the assumption of dependence more appropriate. As we discuss in the Results and Discussion section of the manuscript:*

*“The approximately linear spliced RNA/protein phase plots (Supplementary Figs. 1-6) are qualitatively consistent with the first-order and constant-parameter model of protein production, although we do observe some deviations by cell type. A subset of linear gene/protein pairs (Supplementary Table 1), manually selected from the phase plots according to concordance with the model, was used to estimate the gene-specific protein velocities.”*

*“We hypothesize that the nonlinear behavior corresponds to regulatory differences due to cell type; in the context of our model, the data seem to suggest a unique, low degradation rate in CD4+ T lymphocytes and a different, high degradation rate in all other blood cell types.”*

3) Do the authors assume that antibodies bind their target proteins in 1:1 stoichiometry? What is the evidence for this assumption?

*We describe this assumption in the Quantitative considerations and assumptions section of the Supplementary Note. We assume 1:1 stoichiometry for our analysis; however, even if the stoichiometry is more complex, our analysis of approximation error shows that the estimate quality is not degraded. As we describe:*

*“For binding stoichiometry, assuming saturation, the observed number of observed ADT for proteins is . The protein velocity rate equation is:*

*Plugging in the observable :*

*Expressing in units of ,*

*, so this is an estimate no worse than the a priori that the translation rates are constant for all proteins. Crucially, the sign is preserved.”*

-- None of the results are validated.

*We have performed extensive validation using stochastic simulations. This process is described in the Validation section of the Supplementary Note and shows robust results for RNA and protein velocity extrapolation across a broad range of parameter values. We sampled a six-dimensional parameter space and performed 6,393 stochastic simulations in the parameter space corresponding to the physiology relevant to feature barcoding technologies and the previous work on RNA velocity, as well as 10,000 stochastic simulations corresponding to the domain that could be tractable with quantification of soluble proteins.*

Also, they are not linked to plausible biological interpretation.

*We have restructured the discussion of results to emphasize our findings are reproducible between different datasets, then explicitly address each similarity in the context of known blood cell physiology. As we describe in the Results and Discussion section of the manuscript:*

*“We found that subsets of B cells and of T lymphocytes exhibit strong protein acceleration. We hypothesize that the B cell partitioning corresponds to the differences between cell subtypes, e.g. mature B cells, which are resting (12) and require dedicated T-cell activation (13), and plasma cells, which quickly respond to stimuli (14) and would be expected to have high acceleration on the relevant timescales. The T lymphocyte behavior may reflect recent findings that describe mRNA transcript “pile-up” due to heavily suppressed translation in naïve CD4+ T cells (15), or potential lymphocyte plasticity (16). The monocyte behavior may correspond to the steady-state partitioning between monocyte subtypes (17), such as the transition from classical to non-classical circulating monocytes (18). However, due to the imperfect separation of cell types in the embedding, we caution against over-interpretation of aggregated velocities.”*

Responses to Reviewer #2

In this manuscript, Gorin and colleagues present a bioinformatic analysis framework under the name "protein acceleration" intended as a new tool to study single-cell multi-omics datasets that jointly measure gene expression and protein concentration.

The objective of this work is to extend the concepts presented by the Kharchenko group titled "RNA velocity" (La Manno et al., 2018). The idea behind "RNA velocity" is that by discriminating between nascent and processed transcripts in single-cell RNA-seq and considering a model of transcription, one can determine the rate of change of gene expression (i.e. velocity) in single cells. Note that, in a sense, this allows "extrapolation" of the future state of a cell. The authors effectively review this idea in a recent spotlight article (Svensson and Pachter, 2018).

Gorin and colleagues use the same line of arguments and propose that by collecting information on proteins and processed RNA, it is possible to fit a similar model and obtain insight into the dynamics of gene regulation. Note that, to make this possible, the authors need to assume that there is no separation of time scales for the processes of transcription and translation. This assumption, while a bit unorthodox, it is proved acceptable by at least some of the diagrams s-p.

The most useful contribution from the authors is to analyze, for the first time, RNA-protein diagrams with the explicit intent to derive information of single-cell dynamics on it. Another valuable take-home from the manuscript is the comparison between different techniques of joint RNA/protein quantification, including CITE-seq, REAP-seq, and ECCITE-seq. From this analysis, the authors identify the most promising technology.

The main output of the method proposed here is combining forward extrapolation with a backward extrapolation to allow a visualization of a curved cellular trajectory. The curve is representing the effect on the acceleration on changing the trajectory. Unfortunately, we think, in this fundamental step, there hides a fundamental mistake (Major concern 1) in the paper that invalidates the interpretation of those plots. This is only one of the concerns that we have on the paper.

We have profound esteem for the authors, and we were surprised to find both the presentation, accuracy of statements, and mathematical articulation of the manuscript underwhelming. We believe that the authors would have been capable of producing a high-quality manuscript, but that they probably missed out in the attempt to get this out quickly. I believe this work is far from mature, and the effort to fix it is far beyond the normal scope of a major revision.

Main concerns

1) The authors present a model that allows extrapolation of the protein abundance and RNA abundance by a first-order approximation either into the past or future. However, the extrapolation is only possible in the respective spaces. The authors seem very confused about this point. This is evident from the fact that they visualize both protein velocity and RNA velocity on the same manifold 2d representation and then completely misinterpret what the picture shows. Doing this is completely circular, and if the RNA and protein space overlap, then there is no protein velocity information and we are at steady-state!

*We appreciate this comment, as it made us realize we have not fully characterized the relationship between the different “spaces” Reviewer 2 has in mind. For each visualization, our primary reference frame is that of spliced mRNA (under a PCA/t-SNE transformation). Protein velocity is used to determine the past protein space state, which is transformed into a present spliced mRNA space state via the embedding process. This process is entirely analogous to the original RNA velocity workflow, where RNA velocity is used to determine the future spliced mRNA space state, and transformed into a present spliced mRNA space state. The direction of extrapolation can be mapped to a common manifold using the transition matrix approach introduced in the original RNA velocity publication. Generally, the mapping is performed regardless of the nature of the initial space and the target space, and only requires two things: a similarity measure in the embedding space, to identify neighbors, and a similarity measure in the high-dimensional space, to construct the transition probability. We discuss this process in extensive detail in the Embedding process section of the Supplementary Note:*

*“The estimation of the velocity directions in a low-dimensional embedding closely follows the procedure outlined in the original RNA velocity publication (1). … We postulate that the cell will transition in a direction representable by a weighted sum of directions to the neighbors. Further, we assume that the weights are interpretable as probabilities determined by the alignment between the RNA velocity vector and the directions to the nearest neighbors in high-dimensional space. Thus, for each , we find a transition probability vector … The protein velocity embedding process is identical save for one difference. The vectors and correspond to variance-stabilizing transformations of the protein velocity and the directions to nearest neighbors in high-dimensional protein space.”*

*Further, we explicitly enumerate this assumption in Supplementary Table 3. We now describe how the “reference frame” of mRNA relates to past and future in the background section of the manuscript:*

*“In brief, the current population of unspliced transcripts is slated to be processed (Fig. 1a), and thus contains information regarding the future population of spliced transcripts. We extend this logic as follows (8): the current population of proteins was translated from spliced RNA, and thus contains information regarding the past population of spliced transcripts (Fig. 1b). “*

*The original version of Figure 1 was potentially oversimplified in its depiction of the embedding process. We have revised Figure 1 and added panel 1c to more explicitly explain it.*

2) The "RNA velocity" (first derivative of mRNA levels) in a sense IS the "protein acceleration," (second derivative of protein levels ~= first derivative of mRNA levels) and the authors are considering a system where they have access to both protein velocity and RNA velocity protein acceleration. While in some sections, the authors seem to get this right, there is a general confusion in the paper.

*We agree completely that change in protein levels follows directly from change in mRNA levels; indeed, this is the basis for our analysis.*

The confusion on (1) and (2) is highlighted by the four poorly qualified statements that end up being contradictory:

- "The combination of protein velocities reveals the curvature of the cell state landscape." It is the RNA velocity that is the curvature of the protein space!

*It appears there might have been a formatting error in the manuscript. The sentence cited was supposed to read:*

*“The combination of RNA and protein velocities reveals the curvature of the cell state landscape.”*

*We have verified that the sentence is written correctly in this revision.*

*RNA velocity is the first derivative of RNA counts and related to the second derivative of protein counts. As RNA velocity is a vector with a unique direction and linear (first-order) information, we posit that second-order information is needed to infer curvature; in the current method, we approximate the second-order information by using two first-order vectors.*

- "With respect to the reference frame of unspliced transcripts, the protein inferences correspond to second-order protein acceleration of unspliced transcript counts." This is not true, it is instead the other way around.

*We appreciate the critique, and now recognize that this phrasing is ambiguous and may be interpreted in a fashion opposite of intended. We have rewritten it as follows.*

*“In a conceptual sense corresponding to Fig. 1b, the immediate protein velocity and the underlying RNA velocity yields a second-order estimate of protein acceleration driven by upstream unspliced mRNA modulation.”*

- "We visualize cell movement in the spliced mRNA using a Bézier curve calculated from three points corresponding to past, present, and future." they represent the present and the future of the RNA levels but the past of the protein level!

*This is correct, and we have revised Figure 1, Methods, and the Supplementary Note to emphasize the embedding process. We further emphasize the conversion to the cell state defined by the spliced RNA level, as described in the Results and Discussion section of the manuscript:*

*“We extrapolated the cell states, then embedded them in a projection calculated from the spliced mRNA space… We visualize cell movement in the embedding using a Bézier curve calculated from three points corresponding to past, present, and future cell states.”*

*- "Our method estimates protein translation dynamics from a single timepoint." This is an overstatement, to understand the translation dynamics one would need to estimate all the parameters from a cell population (a subset of which should be sampled at steady state).*

*We replace “dynamics” with “kinetics” to emphasize that this is a more limited statement that only attempts to infer a single kinetic degree of freedom, and includes assumptions about decay rates, translation rates, and structure of the solution, as described in Supplementary Table 3 and the Quantitative considerations and assumptions section of the Supplementary Note.*

3) At the implementation level, it seems that the authors are just adapting, with minimal changes, the code from the package velocyto (La Manno et al., 2018). From inspection, I noticed a trivial substitution of variables, and some of the code confirms that points (1-2) are a key interpretation problem. This is further apparent from the fact that the pipeline is implemented solely in a Jupyter notebook rather than as a package or scanpy or velocyto extension.

*We have released the code as the stand-alone protaccel Python package (available at https://pypi.org/project/protaccel/), and implemented it as a suite of tools integrated into the velocyto Python package (available at https://github.com/gennadygorin/velocyto.py, pull request at https://github.com/velocyto-team/velocyto.py/pull/220). protaccel can be installed by running pip install protaccel in terminal. All Jupyter notebooks have been updated to reflect the change.*

4) It is not clear to me what we learn from acceleration. The authors should illustrate an example where knowing the curvature of the manifold can be somehow related to useful biological conclusions. For example: considering a bifurcation of a progenitor cell type to two alternative linages, can the author's approach help exploring the possible regulatory causes of the bifurcation?

*To our knowledge, there are no such datasets available. In the current publication, we focus on introducing the method, evaluating its current performance across different technologies, validating it in the currently available parameter space, and discussing its applicability to the broader parameter range accessible by future technology (e.g. Supplementary Figures 49-50 and the accompanying discussion). We look forward to the availability of new datasets that will allow such investigations.*

5) Unfortunately, the fact that the authors use publicly available datasets impairs their ability to select a system where a "protein acceleration" might be more relevant and allow them to validate what they observe. I believe that in this scenario there is no way to tell if the predictions are reasonable.

*We agree that the data we used, consisting of terminally differentiated cells, is not expected to have the clear temporal dynamics expected of, e.g., differentiation data. However, the fact that six datasets using four independent technologies show reproducible results in the same system of PBMCs illustrates, to us, that the inferences are meaningful and reproducible, both from the raw data (e.g., the discussion of the CD4 phase portrait and the uniqueness of the CD4+ T cells in Figure 2b) and the downstream results (e.g., the cell type-specific discussion across the datasets in the Results and Discussion section, where we explicitly compare the results and discuss their implications). We consider the range of datasets to be a strength of the current article, and indeed selected them due to their coverage of the same types of cells with different technologies. Due to the nascence of the feature barcoding technology, we emphasize the methodological innovation, consistency between technologies, and the potential extensions. Further, the publicly available datasets already allow us to select the most promising technologies for performing protein acceleration analysis.*

6) The paper does not present any sensitivity analysis or simulation based data. Note that having a situation with simulated data might also help to understand what is the parameter distribution and in which part of the distribution inference is accurate.

*We have performed simulations using the Gillespie algorithm and found that the methods are robust throughout a broad range of parameter values. The process and results are described in the Validation section of the Supplementary Note. The physiologically plausible part of the parameter space yields protein velocity estimates that are as good as or better than RNA velocity estimates. We sampled a six-dimensional parameter space and performed 6,393 stochastic simulations in the parameter space corresponding to the physiology relevant to feature barcoding technologies and the previous work on RNA velocity, as well as 10,000 stochastic simulations corresponding to the domain that could be tractable with quantification of soluble proteins. We discuss the domain of applicability and non-triviality in the Supplementary Note:*

*“As may be expected, high degradation rates deplete molecule counts with information about past cell states and reduce the effectiveness of predictions. Finally, the region with joint high protein and RNA degradation rates exhibits static behavior. Qualitatively, the physiologically realistic parameter space, denoted by a magenta box, exhibits fairly good extrapolation performance for both RNA and protein velocities. The distributions of correlations within each respective region are shown in Supplementary Figure 47. The mean correlation between RNA velocity and the forward difference in spliced counts is 0.57, with an empirical 90% confidence interval of (0.29, 0.83). The mean correlation between protein velocity and the backward difference in protein counts is 0.66, with an empirical 90% confidence interval of (0.30, 0.94). This analysis demonstrates that the performance of protein velocity estimation is no worse, and often better, than that of RNA velocity within the space of parameters expected in living systems.”*

La Manno, G., Soldatov, R., Zeisel, A., Braun, E., Hochgerner, H., Petukhov, V., Lidschreiber, K., Kastriti, M.E., Lönnerberg, P., Furlan, A., et al. (2018). RNA velocity of single cells. Nature 560, 494-498.

Svensson, V., and Pachter, L. (2018). RNA Velocity: Molecular Kinetics from Single-Cell RNA-Seq. Mol. Cell 72, 7-9.

Responses to Reviewer #3

Gorin et al. build on previously established RNA velocity methods and develop a method that captures unspliced, spliced, and protein production kinetics from single-cell multi-omics data. The authors acquired GEO datasets from CITE-seq, REAP-seq, and ECCITE-seq experiments, to predict future states of cells by inferring kinetic trajectories of single cells from peripheral blood mononuclear cell (PBMC) datasets.

Overall, this study provides a novel analysis in the recently emerging RNA velocity field. Specifically, the authors expand on current methods incorporating protein abundance data from single-cell multi-omics data to be able to infer the past state of cells, in addition to the present and future state inferred from RNA transcript abundance. Secondly, the data acquired in this current study utilizes single-cell data, allowing one to capture heterogeneity in a population of cells. Finally, previous RNA velocity papers involved measuring RNA abundances at several time points in the experiment. The present analysis requires information from a single time point. For these reasons, I believe this study is suitable for publication, with minor revisions indicated below.

* (Page 3) "for RNA velocity, we used a broad panel of genes with robust unspliced detection" - were all genes indicated in the supplemental figure considered 'robustly detected'? There are a few genes that have very little detection.

*We clarify the filtering process in the Velocity calculation section of the Supplementary Note; this process is consistent with the velocyto package implementation. Further, Supplementary Figures 7-12 now display 24-gene phase portrait panels, selected from the entire set of “velocity genes.” The submission version of the RNA velocity figures only showed spliced/unspliced phase portraits for genes coding for the observed proteins. These genes were not generally robust enough to perform RNA velocity. Since these genes are not used in the calculation of RNA velocity, we omit their visualization. As we describe in the aforementioned section:*

*“Since not all genes are expected to express dynamics consistent with the simple ODE model, we filter the selection of genes used for the velocity calculations. For RNA velocity, we perform three rounds of filtering, omitting genes with a low coefficient of variability relative to mean, extremely low expression, and low of the phase plots relative to the linear fit produced to estimate .”*

* (Page 3) "A subset of high-abundance gene/protein pairs were used…" How were these genes specifically selected, or was it random?

*As discussed in the Velocity calculation section of the Supplementary Note, we manually selected gene/protein pairs based on qualitative concordance between their phase plot and the diagonal phase plot expected from the model:*

*“Due to the low dimensionality of the protein data, we manually select spliced RNA-protein pairs for protein velocity; we omit gene/protein pairs that have phase portraits without a self-evident linear component, and choose only a single subunit for analysis (e.g. CD3D/CD3 rather than all 3 subunits of CD3).”*

*We further make this selection process explicit in the Methods section of the manuscript:*

*“To calculate protein velocities, we followed the same process, albeit using protein/spliced RNA phase plots and manually selecting “velocity proteins” with qualitatively linear phase plot appearance.”*

*Additionally, "we used a broad panel of genes with robust unspliced detection" - a list of these genes should be present in the supplementary table. A table for protein velocity is presented, but not for the RNA velocity measurements.*

*Table 1 provides the number of “velocity genes” for each dataset, ranging from 591 for ECCITE-seq to 1338 for REAP-seq. For completeness and reproducibility, we provide the supplementary file gsp_2019_rna_velocity_gene_list.csv, which lists all of the “velocity genes” in each analysis.*

* (Figure 1) The diagram is intuitive and easy to read. However, the authors state that a "broad panel of genes" with "robust unspliced detection" were selected for study in this paper. Would readers benefit from one example gene being shown with actual data in the same manner as figure 1B?

*To facilitate reader evaluations of the quality of RNA velocity inference, Supplementary Figures 7-12 now display 24-gene phase portrait panels with fits for each dataset, Table 1 gives the total number of velocity genes, and the supplementary file gsp_2019_rna_velocity_gene_list.csv lists all velocity genes.*

* (Page 5) "This behaviour may reflect recent findings [12] that describe mRNA transcript "pile-up" due to heavily suppressed translation…" - this might explain the strong protein acceleration in T lymphocytes. Can the authors speculate on B cells exhibiting high protein acceleration?

*We posit that the effect in B cells is explained by B cell subtypes exhibiting different dynamics; e.g., low acceleration for mature B cells and high acceleration for Bregs. This is further discussed in the Results and Discussion section:*

*“We found that subsets of B cells and of T lymphocytes exhibit strong protein acceleration. We hypothesize that the B cell partitioning corresponds to the differences between cell subtypes, e.g. mature B cells, which are resting (12) and require dedicated T-cell activation (13), and plasma cells, which quickly respond to stimuli (14) and would be expected to have high acceleration on the relevant timescales.”*

* (Page 5) "The unidirectional monocyte velocity suggests response or plasticity" - Could this point be elaborated? Which response might be elicited? Or rather, what scenario could result in the observed monocyte velocity? The reference associated with this statement also refers to T-cell lineage commitment and plasticity.

*We appreciate the call for clarity, as we now realize the description may be made more concrete. We posit that the monocyte velocity may correspond to unidirectional transitions between monocyte subtypes at steady state, as we state in the Results and Discussion section of the manuscript:*

*“The monocyte behavior may correspond to the steady-state partitioning between monocyte subtypes (17), such as the transition from classical to non-classical circulating monocytes (18).”*

* (Page 5) "…partitioning into static and mobile populations…" - Would it benefit readers if the static and mobile populations were labeled in the supplementary figure?

*The identification of relatively static, mobile, and accelerated populations is one of the main uses of the embedding plots with explicitly drawn vector fields (Supplementary Figures 38-43). We believe the arrows, together with the cell type colors, communicate the partitioning without explicit labeling.*

* (Page 6) "Future work may involve better measurements…" - of what? Could a specific example be given?

*We have made the future work discussion more concrete, focusing on cell type-specific behaviors and the quantification of transcription factors by feature barcoding:*

*“Our qualitative protein acceleration framework does not attempt to account for regulatory differences between cell types. Future work may involve more granular models to enable inference of local rather than global parameters, e.g. the determination of separate parameters for the CD4+ T lymphocytes and other cell types for the CD4 gene (Fig. 2b). Current protein quantification protocols are adapted for histological markers on the cell surface; technology that can quantify cytosolic protein could aid in more extensive studies of cell dynamics and open a broader range of investigations tractable by protein acceleration, as discussed in the Supplementary Note. Finally, the simultaneous quantification of mRNA and regulatory cytosolic proteins would greatly aid in the implementation of physically realistic models of gene expression which explicitly account for regulation by observed transcription factors.”*

* (Supplementary Fig. 19 & 20) The arrow and arrow colour are not easily distinguished from the velocity fields.

*We have decreased the opacity of the cells to make the arrows more distinct. Further, we have provided a Python package that allows the user to easily tune the visualization parameters.*

**Second round of review**

**Reviewer 1**

The authors have discussed some of their assumptions, in particular they added Supplementary Table 3 with assumptions. The clarity of the figures and the description in the main text are improved. These are certainly steps in the right direction but my fundamental concerns are not addressed:

The authors wrote in their response that "We provide an outline of significant assumptions within the estimation framework and explain why they are equivalent to or better than assumptions in the original publication in Supplementary Table 3." Yet, Table S3 does not explain why synthesis rates are unity. It simply asserts they are equivalent. The table generally does not explain or substantiate assumptions -- it just asserts them, mostly in comparison to RNA velocity rather than on the basis of fact and merit.

They authors also referenced Xiao and Wu (DOI: 10.1021/acs.analchem.7b02241) as evidence for unit synthesis rates. Xiao and Wu did measure synthesis rates of 847 N-glycoproteins and found those to vary over about 10-fold. Hardly a unity synthesis rate. More importantly these were normalized rates / per hour rather than molecules per hour that would reflect protein abundances. Also, Xiao and Wu did not measure RNA and their estimates have nothing to say about proteins synthesized per RNA, which is the quantity pertinent to this discussion.

The authors report that stochastic simulations from models that conform to the assumptions of the authors confirm their method and inferences. The key question is how this relates to data generated from biological systems that do not conform to their assumptions.

**Reviewer 2**

1) I am pleased that the comment has allowed the authors to reflect more about the relation between velocity and different spaces. This issue is now resolved.

2) Resolved. Now there are no confusing statements left in the text regarding velocity/acceleration.

Still, the title maintains traces of the original ambiguity: RNA velocity and protein acceleration are both are estimated from unspliced, and they are very closely related. I believe the main novelty of the paper is to compute protein velocity (first derivative of protein abundance) and its combination with RNA velocity to get a protein acceleration.

All this to say that the author should consider changing the paper title to “Protein velocity and acceleration from …” or “RNA velocity and protein velocity from …".

(3) Resolved, very good response.

(4) & (5) Not resolved. Method motivation is still missing.

I agree with the authors that the scope of this work does not need to deviate from the one originally indented. That is: it is ok to develop a method with the data available without performing further experiments. However, note that, with comments 4 and 5, I was not demanding a biologically relevant proof through further experiments or the analysis of an existing dataset.

Instead, I was strongly suggesting that the authors showcase which kind of biological insights acceleration could offer, for example, providing a proof of principle scenario (note: explicitly crafting a simulated data for the purpose is perfectly ok).

I think that doing so would be very important to clarify what kind of biological question the method is intended answering. At the moment, this point is not clearly addressed.

It remains unclear to me what would motivate a researcher to perform CITE-seq, or similar protocols, to get protein velocity and acceleration. What biology could one discover? Does one get a better extrapolation of cell state not accessible otherwise? Could one predict cell fate commitment earlier than with RNA velocity? Could one capture some correlation between protein acceleration and another feature that is suggestive of interesting biology? Could one interpret cell with high curved trajectory as undergoing some specific process? These are just a list of hypothesis I am proposing to authors, just to make my request concrete. Addressing one or two points in the spirit of the questions above (not all, nor specifically the ones I point out) would be valuable to frame the benefit of computing acceleration.

(6) Resolved, excellent response.

Note:

I think the responses of the authors to Reviewer #1’s comments 1 and 2 make perfect sense. I think the authors have very reasonable claims in response to that criticism, just pointing it out in case Reviewer 1 would not result satisfied.

**Authors Response**

**Point-by-point responses to the reviewers’ comments:**

Responses to Reviewer #1

The authors have discussed some of their assumptions, in particular they added Supplementary Table 3 with assumptions. The clarity of the figures and the description in the main text are improved. These are certainly steps in the right direction but my fundamental concerns are not addressed:

The authors wrote in their response that "We provide an outline of significant assumptions within the estimation framework and explain why they are equivalent to or better than assumptions in the original publication in Supplementary Table 3." Yet, Table S3 does not explain why synthesis rates are unity. It simply asserts they are equivalent. The table generally does not explain or substantiate assumptions -- it just asserts them, mostly in comparison to RNA velocity rather than on the basis of fact and merit.

*We have expanded the Quantitative considerations and assumptions section of the Supplementary Note to clarify the assumptions and discuss their physiological basis. We compare our assumptions to those of RNA velocity as a useful baseline. The substantiation of assumptions behind RNA velocity is outside the scope of the current article.*

They authors also referenced Xiao and Wu (DOI: 10.1021/acs.analchem.7b02241) as evidence for unit synthesis rates. Xiao and Wu did measure synthesis rates of 847 N-glycoproteins and found those to vary over about 10-fold. Hardly a unity synthesis rate. More importantly these were normalized rates / per hour rather than molecules per hour that would reflect protein abundances. Also, Xiao and Wu did not measure RNA and their estimates have nothing to say about proteins synthesized per RNA, which is the quantity pertinent to this discussion.

*We appreciate you bringing this to our attention. We have estimated transcriptome-wide and CD protein-specific translation rates using orthogonal ribosome profiling data. As we discuss in the Quantitative considerations and assumptions section of the Supplementary Note:*

*“Transcriptome-wide measurements of per-mRNA eukaryotic initiation rates … may be estimated from auxiliary data. … we use the ribosome density as a proxy for the distribution of initiation rates. Specifically, we calculate the differences in expression between ribosome-protected and control samples. The ribosome profiling expression per unit length is determined by underlying expression per unit length and the ribosome density; by normalizing the ribosome-protected expression by the total expression, we estimate the ribosome density. We follow Siwiak and Zielenkiewicz (5) in using HeLa ribosome profiling expression data from Guo et al. (6), and follow Guo et al. in the interpretation of normalized expression as a reflection of ribosome density.*

*The resulting estimates, shown in Supplementary Figure 44, suggest that the expression change for the cluster of differentiation genes is distributed within approximately one order of magnitude (std of log fold change = 0.251), compared to three orders of magnitude (std of log fold change = 0.428) for the entire transcriptome. Therefore, assuming similar translation rates for the cell surface markers used in the feature barcoding technologies appears to be no more problematic than assuming similar splicing rates for several thousand genes in the transcriptome.”*

*We suggest several approaches to accounting for the differences in protein translation rates and provide them as options in the Python implementation of the method. As we discuss in the Methodological directions, quantitative and qualitative section of the Supplementary Note:*

*“The first approach requires estimates of splicing and translation rates from an orthogonal technology … Although currently available datasets do not have translation rates for the protein targets accessible by feature barcoding technologies – for example, the CD proteins quantified in recent studies (13,16) have essentially no overlap with the surface proteins investigated by CITE-seq, REAP-seq, ECCITE-seq, or 10X Feature Barcoding – we anticipate that the future compilation of estimates for these rates will improve the embedding process. …*

*The second approach does not require any external information, and simply uses the signs of velocities and displacements to estimate the transition matrix. … We have found that the RNA velocity landscapes are fairly well reproduced through this method (Supplementary Figure 53, cf. La Manno 2018, Fig. 4a). Our preliminary investigations have been able to recapitulate significant qualitative features of the protein acceleration landscape (Supplementary Figure 54, cf. Supplementary Figure 55). However, the sparsity of available data – at most thousands of cells with at most dozens of protein markers – makes this method rather sensitive. … The further development of binary correlation metrics is particularly valuable because of its ability to sidestep the O(1) corrections necessary to quantitatively account for stoichiometry, localization, and binding saturation. …”*

The authors report that stochastic simulations from models that conform to the assumptions of the authors confirm their method and inferences. The key question is how this relates to data generated from biological systems that do not conform to their assumptions.

*The model is necessarily limited by its assumptions. However, we use a combination of internal (e.g. phase plot appearance) and external (e.g. ribosome density) information to select genes that do appear to conform to assumptions, as well as to increase confidence in the assumptions. The development of more general classes of models, as well as more robust approaches to embedding, is a natural extension of the current work and we plan to pursue it in the future.*

Responses to Reviewer #2

1) I am pleased that the comment has allowed the authors to reflect more about the relation between velocity and different spaces. This issue is now resolved.

2) Resolved. Now there are no confusing statements left in the text regarding velocity/acceleration.

Still, the title maintains traces of the original ambiguity: RNA velocity and protein acceleration are both are estimated from unspliced, and they are very closely related. I believe the main novelty of the paper is to compute protein velocity (first derivative of protein abundance) and its combination with RNA velocity to get a protein acceleration.

All this to say that the author should consider changing the paper title to “Protein velocity and acceleration from …” or “RNA velocity and protein velocity from …".

*We have changed title of the article to “Protein velocity and acceleration from single-cell multiomics experiments.”*

(3) Resolved, very good response.

(4) & (5) Not resolved. Method motivation is still missing.

I agree with the authors that the scope of this work does not need to deviate from the one originally indented. That is: it is ok to develop a method with the data available without performing further experiments. However, note that, with comments 4 and 5, I was not demanding a biologically relevant proof through further experiments or the analysis of an existing dataset.

Instead, I was strongly suggesting that the authors showcase which kind of biological insights acceleration could offer, for example, providing a proof of principle scenario (note: explicitly crafting a simulated data for the purpose is perfectly ok).

I think that doing so would be very important to clarify what kind of biological question the method is intended answering. At the moment, this point is not clearly addressed.

It remains unclear to me what would motivate a researcher to perform CITE-seq, or similar protocols, to get protein velocity and acceleration. What biology could one discover? Does one get a better extrapolation of cell state not accessible otherwise? Could one predict cell fate commitment earlier than with RNA velocity? Could one capture some correlation between protein acceleration and another feature that is suggestive of interesting biology? Could one interpret cell with high curved trajectory as undergoing some specific process? These are just a list of hypothesis I am proposing to authors, just to make my request concrete. Addressing one or two points in the spirit of the questions above (not all, nor specifically the ones I point out) would be valuable to frame the benefit of computing acceleration.

*We appreciate this comment, as it prompted us to contextualize the velocity estimation methods, both with respect to one another and with respect to the problems currently pursued by the scRNA-seq field. Protein velocity has a technical advantage of enabling backward and long-time horizon extrapolation, as well as broader applications to the estimation of vector fields from sparse single-cell data. As we discuss in the Directions for applications section of the Supplementary Note:*

*Firstly, the significantly longer timescale of protein degradation allows extrapolation across longer timescales than permitted by RNA velocity... in the current state, protein velocity is the sole method suited to hour-scale and backward extrapolation from single-time point data without chemical labeling. Protein velocity invites natural comparisons to chemical labeling data with multi-hour sampling or treatment times. A recent preprint by Cao et al. (18), investigating cell cycle dynamics by thiol labeling, observed fairly substantial differences between trajectories inferred from RNA velocity and from the experiment timing. We expect that part of this discrepancy is attributable to RNA velocity reflecting short-time horizon, “high-frequency” dynamics and the labeling reflecting long-time horizon, “low-frequency” dynamics, and suggest that analysis based on protein abundance may be more comparable. More broadly, the extended timescale may be useful for datasets with a sparsely observed cellular landscape, such that the high-frequency RNA velocity and low-frequency neighborhood cease to correlate.*

*Secondly, in the short-time limit, the availability of a single observed and two extrapolated points lends itself to the description of non-linear behavior. RNA velocity corresponds to a single extrapolation ray, and can most effectively describe linear traversal of a manifold. We postulate that the additional "curvature" information can provide a route toward description and inference of more sophisticated regulation mechanisms and developmental landscapes. This approach has, for example, been adopted by Qiu et al. in a recent preprint using metabolic labeling for cell state vector field reconstruction (19). Qiu et al. use dynamic information from single cells to learn vector fields underlying differentiation processes, and we anticipate that protein acceleration will provide a useful data source for such methodologies. The applications range from quantitative, e.g., directly using the protein velocity information to reconstruct vector fields, to qualitative, e.g., using the sign of the protein velocity to validate findings from RNA-based reconstruction methods.*

(6) Resolved, excellent response.

**Third round of review**

**Reviewer 2**

The new discussion added in the supplementary material can be considered a sufficient response to my review.
